# Novel Bifunctional [16]aneS_4_-Derived Chelators for Soft Radiometals

**DOI:** 10.3390/molecules26154603

**Published:** 2021-07-29

**Authors:** Natan J. W. Straathof, Charlotte B. Magnus, Fedor Zhuravlev, Andreas I. Jensen

**Affiliations:** Department of Health Technology (DTU Health Tech), Technical University of Denmark (DTU), Frederiksborgvej 399, 4000 Roskilde, Denmark; njwst@dtu.dk (N.J.W.S.); chbusk@dtu.dk (C.B.M.); fezh@dtu.dk (F.Z.)

**Keywords:** sulfur containing macrocycles, bifunctional chelators, bio-conjugation, radiation therapy

## Abstract

The field of targeted radionuclide therapy is rapidly growing, highlighting the need for wider radionuclide availability. Soft Lewis acid ions, such as radioisotopes of platinum, rhodium and palladium, are particularly underdeveloped. This is due in part to a lack of compatible bifunctional chelators. These allow for the practical bioconjugation to targeting vectors, in turn enabling radiolabeling. The [16]andS_4_ macrocycle has been reported to chelate a number of relevant soft metal ions. In this work, we present a procedure for synthesizing [16]andS_4_ in 45% yield (five steps, 12% overall yield), together with a selection of strategies for preparing bifunctional derivatives. An ester-linked N-hydroxysuccimide ester (NHS, seven steps, 4% overall yield), an ether-linked isothiocyanate (NCS, eight steps, 5% overall yield) and an azide derivative were prepared. In addition, a new route to a carbon-carbon linked carboxylic acid functionalized derivative is presented. Finally, a general method for conjugating the NHS and NCS derivatives to a polar peptide (octreotide) is presented, by dissolution in water:acetonitrile (1:1), buffered to pH 9.4 using borate. The reported compounds will be readily applicable in radiopharmaceutical chemistry, by facilitating the labeling of a range of molecules, including peptides, with relevant soft radiometal ions.

## 1. Introduction

The field of nuclear medicine has seen remarkable growth in recent years, in particular within oncology. Here, radionuclides emitting ionizing radiation are used for either diagnosis or therapy, the two modalities in turn combined under the umbrella of theranostics [1]. New theranostic platforms, such as PSMA targeted ligands for prostate cancer, have driven the surge in interest [2], with the therapeutic ^177^Lu-PSMA-617 just completing clinical phase 3 with a positive outcome [3].

**Figure 1:**
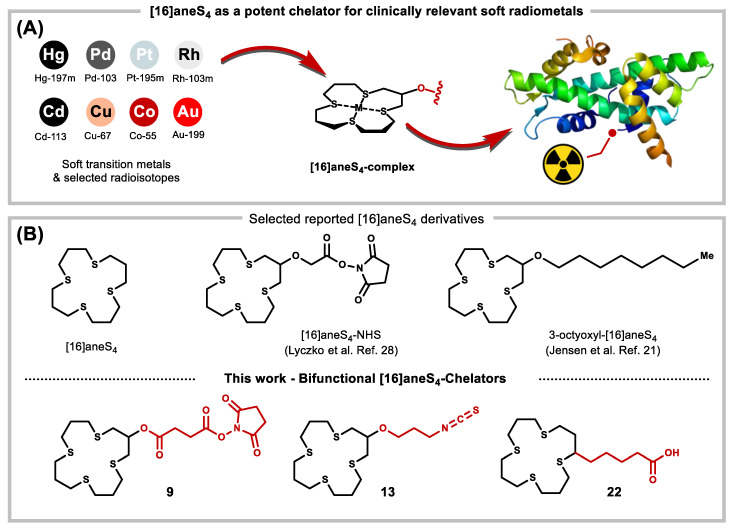
(**A**) Bifunctional [16]aneS_4_ chelators as a general strategy for soft radiometal complexes for bio-conjugation, with application in targeted radiotherapy and radiodiagnostics. (**B**) Examples of reported [16]aneS_4_ chelator derivatives and the novel [16]aneS_4_ bifunctional chelators reported in this manuscript.

In nuclear medicine, radiopharmaceuticals are typically constructed as conjugates of receptor-specific vectors with radionuclides. For radiometals, this requires the use of chelators, capable of trapping the radiometal ions as stable complexes (Figure 1A). This strategy can effectively deliver radionuclides to target tissues such as tumors. Diagnostic imaging by positron emission tomography (PET) is most commonly performed using radionuclides such as ^15^O, ^13^N, ^11^C, ^68^Ga and ^18^F [4,5]. Other PET radiometals of more limited use include ^64^Cu, ^55^Co and ^86^Y. Concomitantly, targeted radionuclide therapy (TRT) typically focuses on eradicating cancer metastases via alpha, beta or Auger electron radiotherapy (AeRT) [6,7,8]. Metastasis is a major reason for cancer recurrence and is considered responsible for up to 90% of cancer-related deaths [9], making TRT highly relevant. AeRT is considered particularly promising for eradicating micro-metastases, due to the unique short range and potency of Auger electron emissions [10,11,12,13,14].

Current therapeutic radionuclides of high interest include the beta particle emitters ^177^Lu, ^131^I, ^90^Y and ^67^Cu, and the alpha particle emitters ^225^Ac, ^227^Th and ^211^At. The most common Auger electron emitters used in current research are ^123/125^I, ^67^Ga, ^111^In and ^99m^Tc, largely as a result of their availability and established radiochemistry [13,15,16,17,18,19,20]. Radiometals that form soft Lewis acid cations are also available. These soft radiometals have a particularly high binding affinity to sulfur-containing ligands (vide supra). However, the range of soft radiometals relevant to theranostics is rarely investigated, due to a lack of well-suited bifunctional chelators with active chemical handles that can be practically conjugated to targeting vectors (Figure 1A). Suitable soft radiometals for AeRT include isotopes of platinum (^193m^Pt and ^195m^Pt) and rhodium (^103m^Rh) [21,22]. Additionally, ^103^Pd and ^105^Rh, an X-ray emitter and a beta particle emitter, respectively, also have potential use in TRT.

To address this deficiency, we identified [16]aneS4 (1,5,9,13-tetra thiacyclohexadecane) as a potential chelator for late transition radiometals. [16]aneS4 belongs to a larger class of crown ethers, which includes widely used oxygen donor macrocycles, such as 18-crown-6 [23]. While oxygen-based crown ethers strongly bind alkali and alkaline earth metal ions, their thioether analogues show distinct preference for the much softer late transition metals [24]. The complexation of [16]aneS4 (and derivatives thereof) with various transition metals has been studied intensively (Table 1) [25,26,27,28,29,30,31,32,33,34,35,36,37,38,39,40]. Its application in radiopharmaceutical chemistry remains limited, however, and to date only a few examples have demonstrated complexation between a soft radiometal and [16]aneS_4_. Lyczko et al. successfully demonstrated the formation of a stable Rh(III) complex formed via the use of 1,5,9,13-tetrathiacyclohexadecane-3,11-diol and 1,5,9,13-tetrathiacyclohexadecane-3-ol (respectively, [16]aneS_4_-diol and [16]aneS_4_-ol) [28]. This complex was used for binding radioiodine and ^211^At using the chelated Rh as coordination center. Moreover, [16]aneS_4_ has been studied in relation to ^105^Rh for in vivo bio-distribution [26,30]. To highlight the potential application of such sulfur-containing macrocycles, an overview of the reported metal-[16]aneS_4_ complexes is provided in Table 1. Accordingly, expanding the chemical space of bifunctional [16]aneS_4_ derived chelators would be beneficial, especially in the context of soft radiometal delivery. In this report we present a synthetic framework for the preparation of bifunctional [16]aneS_4_ chelators (Figure 1B, bottom).

## 2. Results and Discussion

The synthesis of [16]aneS_4_ was initially reported by Meadow and Reid in 1934, who identified the difficulty of the formation of rings containing more than 12 members (e.g., [16]aneS_4_ was obtained in 1% yield under the reported conditions) [31]. As mentioned above, the synthesis of [16]aneS_4_-diol and [16]aneS_4_-ol, was disclosed later, using a multistep approach to prepare the asymmetric analogues of [16]aneS_4_ [28,29]. This strategy originated from the seminal report on an alternative synthetic strategy for sulfur-containing macrocycles by Ochrymowycz, in 1973 [32]. While the overall yield was slightly improved (5% yield), the effects observed during ring formation were of particular importance, suggesting that, for example, a chloro leaving groups and lower solvent polarity favored larger ring structures.

**Figure 2:**
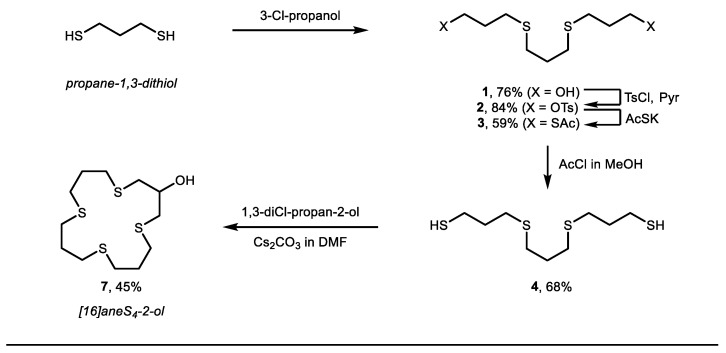
Synthesis of [16]aneS_4_-ol (see SI for further details).

On this backdrop, several different strategies were considered, accounting for synthetic practicality and compound stability. To broaden the application of the new macrocyclic chelator derivatives, three different linker types were prepared (Figure 1B, bottom), all with the potential to further the use of soft radiometals in nuclear medicine. Our synthetic approach started with the multistep synthesis to [16]aneS_4_-ol **7**, using a method adapted from Lyczko and Li [28,29], and the crucial findings from the reports of Meadow, Reid and Ochrymowycz [31,32]. Starting with 1,3-dithiopropane and 3-chloropropanol, gave 1,11-dihydroxy-4,8-dithiaundecane **1** in moderate yield (76%, Figure 2). Transformation of the hydroxyls to thiols was accomplished with consecutive tosylation of the hydroxyl groups, thioacetylation and hydrolysis, which give linear product 1,11-dithio-4,8-dithiaundecane **4** in 34% yield over 3 steps. The final macrocyclization was performed with 1,3-dichloropropan-2-ol and 1,11-dithio-4,8-dithiaundecane **4**, at high dilution and slow addition (multiple hours) of a mixture of both reactants in DMF and with Cs_2_CO_3_ as base (see SM for full details). Gratifyingly, this procedure gave the desired macrocycle [16]aneS_4_-ol **7** (45% yield, 12% overall yield).

**Figure 3:**
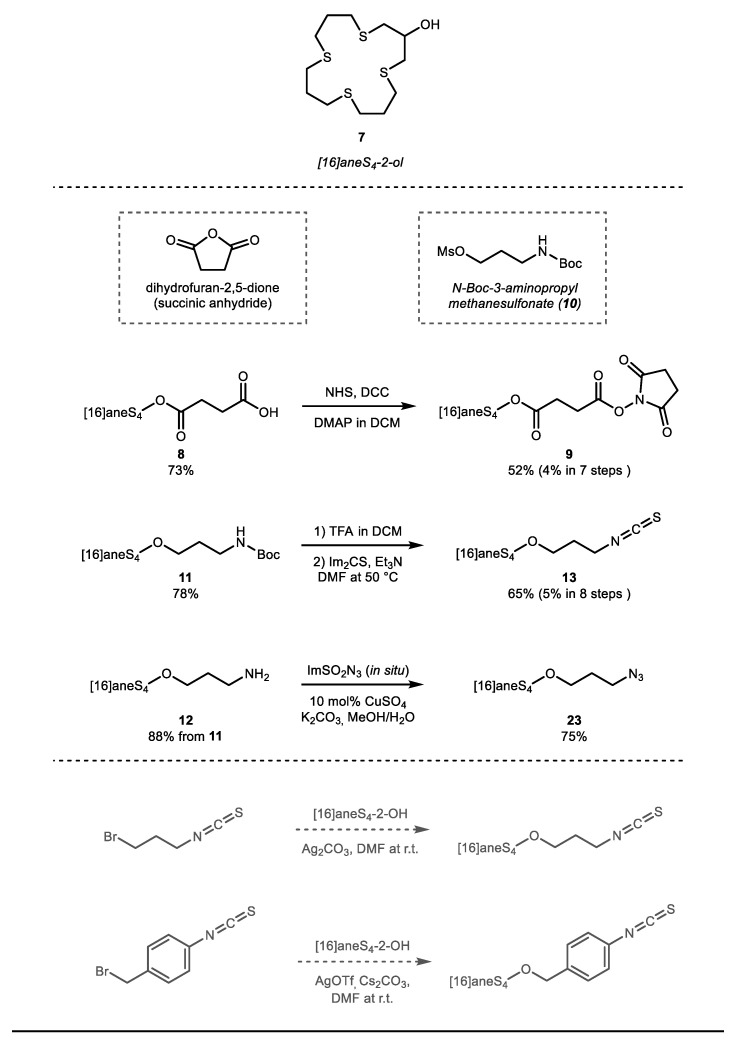
Synthesis of bifunctional [16]aneS4 chelator derivatives, including the NHS ester, isothiocyanate and azide derivative.

With macrocycle [16]aneS_4_-ol **7** in hand, we began the construction of the functional conjugation sites (Figure 3). Previously, [16]aneS_4_-ol **7** was functionalized with α-bromo ethylacetate by Lyczko [28]. In order to construct an ester-linked analogue, [16]aneS_4_-ol **7** was conjugated with succinic anhydride to give carboxylic acid **8** (73% yield). Ester-linked chelators could have a potentially faster catabolism in vivo, which has been recognized as enabling a faster radionuclide clearance, which is potentially desirable for long-lived therapeutic radionuclides [41]. The carboxylic acid intermediate **8** was then activated and transformed into the corresponding NHS ester as compound **9** (52% yield, 4% overall yield in 7 steps). In addition, [16]aneS_4_-ol **7** was decorated with N-Boc-3-aminopropyl methane sulfonate to give the N-protected intermediate **11** (78% yield). This compound was then deprotected under acidic conditions and transformed into the corresponding isothiocyanate via consecutive reaction of the amine with N,N′-thiocarbonyldiimidazole under alkaline conditions (see SM for more details). Via this route, isothiocyanate **13** was obtained in a relative straightforward fashion (65% yield, 5% yield in 8 steps). Notably, the amino intermediate **12** was also transformed directly into the corresponding azido compound **23** using a diazo transfer reagent (75% yield). Azides are extremely useful due to their practical bioconjugation via azide–alkyne cycloadditions under various conditions in vitro and in vivo [42].

The route towards [16]aneS_4_-isothiocyanate **13** proved to be sufficient to be a versatile synthetic framework for alternative strategies. While the overall yield of [16]aneS_4_-ol **7** could be improved further by using the suggestions from Meadow, Reid and Ochrymowycz (vide infra) [31,32], we questioned whether it was possible to directly prepare **13** from **7** in a single step reaction with isothiocyanatobenzyl bromide or 1-bromo-3-isothiocyanatopropane. Both isothiocyanatobenzyl bromide or 1-bromo-3-isothiocyanatopropane are commercially available [43], and combining these with Ag_2_CO_3_ as a concomitant base and halogen abstraction reagent, we questioned whether it would be possible to transform [16]aneS_4_-ol **7**, directly into the corresponding [16]aneS_4_-isothiocyanate **13** (Figure 3, bottom section). Notably, direct alkylation of [16]aneS_4_-ol **7** with 3-bromo propaneisothiocyanate or 4-(bromomethyl) phenyl-isothiocyanate, resulted in only trace amounts of desired products.

**Figure 4:**
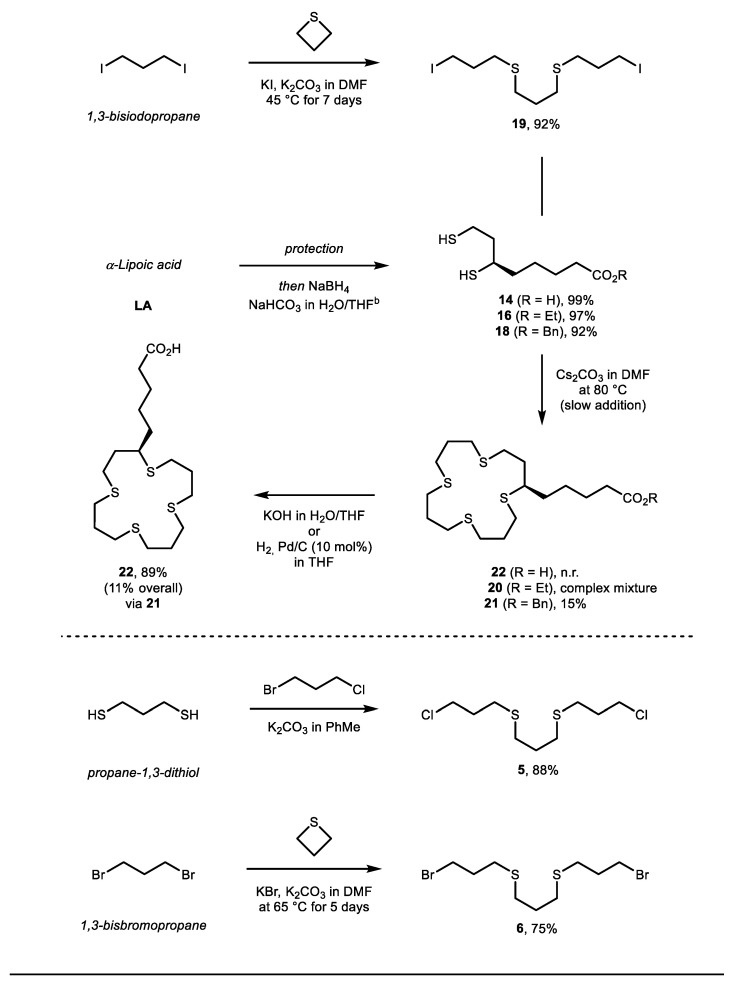
Alternative synthesis of bifunctional [16]aneS4 chelator.

In a further attempt to shorten the synthetic route, and to improve the overall product yield, an alternative route was considered (Figure 4). Inspired by Bagchi et al. [34], 1,3-diiodopropane and two equivalents of thiacyclobutane (thietane) were mixed and stirred with KI, K_2_CO_3_ in DMF for several days at 45 °C. This gave 1,11-diiodo-4,8-dithiaundecane **6** in good yield (87–95% yield, 1.5 gram scale). At this point we wanted to evaluate whether we could use dihydrolipoic acid (DHLA) as the 1,3-dithio coupling partner in the final macrocyclization. DHLA can be prepared quantitatively from α-lipoic acid under mild reducing conditions; NaBH_4_, NaHCO_3_ in H_2_O (see SI for details) [44]. The fact that LA is commercial, inexpensive and relatively facile to transform into its protected and reduced analogues (compounds **13** and **14**, 75% and 82% yield, respectively) is very attractive. Despite numerous efforts, macrocyclication with DHLA or **13** were unsuccessful, and resulted in no reaction or complex inseparable mixtures. Cyclization with benzyl protected DHLA analogue **14** gave the desired macrocycle **17** in modest macrocyclization yield (15%). However, in light of the low overall yields of the formations of bifunctional chelators derived directly from [16]aneS_4_-ol **7**, macrocyclization with DHLA to form **22** appears to be an attractive gateway to functionalizable derivatives. Further attempts to improve the yield of this step were unsuccessful, and it was observed that 1,11-diiodo-4,8-dithiaundecane **6** was unstable and could not be stored for several days, even under inert atmosphere (argon) at −20 °C (see SM for more details and discussion). Based on these observations, we hypothesized that **6** rapidly decomposes due to the presence of iodide, via a reversed sulfonylation degradation pathway or via polymerization. The instability of these compounds was also attributed to external factors such as light and heat, including the presence of heavy halogens (such as iodine), which was also observed by Meadow and Reid [31]. To study the effects of different (halogen) leaving groups, the chloro and bromo analogues of 1,11-dihalogen-4,8-dithiaundecane were prepared. 1,11-dichloro-4,8-dithiaundecane **19** and 1,11-dibromo-4,8-dithiaundecane **20** were also prepared via different routes (Figure 4 bottom, see SI for more details) [45,46]. Unfortunately, these alternative coupling electrophiles, with a bromo, chloro or tosyl leaving group, respectively, did not yield any significant improvement over the previous obtained macro cyclization with **18** and **19**.

**Figure 5:**
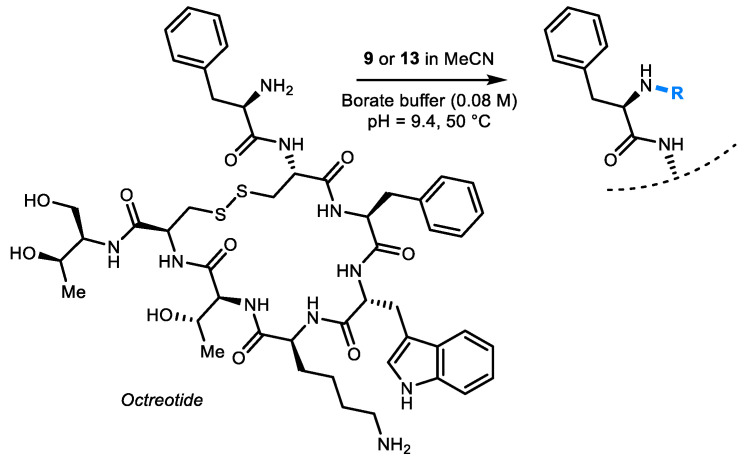
Bioconjugation of **9** and **13** with octreotide (hypothetical selective N-terminal functionalization shown for clarity).

With the successful preparation of four novel [16]aneS4 chelator derivatives, of which three with a reactive functional chemical handle, specifically, [16]aneS_4_-NHS **9**, [16]aneS_4_-NCS **13**, [16]aneS_4_-N_3_ **23**, we elected **9** and **13**, to be conjugated to octreotide, as a proof-of-concept for further conjugation studies. Octreotide is a mimic of natural somatostatin, which is a growth hormone-inhibiting hormone, which regulates the endocrine system, affecting neurotransmission and cell proliferation [47]. Octreotide is used in various therapeutic settings, such as carcinoid tumor care, treating hepatic metastases and palliation. Further, it represents the most widely used peptide vector in current radionuclide theranostics, together with its close analogue octreotate. Octreotide is therefore a relevant candidate for bioconjugation. Briefly, octreotide was dissolved in aqueous borate buffer (80 mM) at pH = 9.4, and mixed in a 1-to-7 molar ratio with **9** or **13** in MeCN at 50 °C (400 µL, aq. buffer:MeCN, 1:1, *v*/*v*, see SM for more details). These reactions’ conditions resulted in clear solutions with no precipitation of either chelator derivatives or peptide. Both conjugates were successfully grafted to octreotide as judged by MALDI-TOF mass spectrometry analysis (Figure 5). This preliminary conjugation study indeed demonstrates that the newly prepared [16]aneS_4_ analogues can be conjugated to compounds of high interest under relevant reaction conditions, with optimization of these conditions being a subject for future research.

## 3. Materials and Methods

Materials, methods and copies of NMR spectra of all compounds are attached in the Supplementary Materials (SM).

## 4. Conclusions

In conclusion, a number of novel bifunctional chelators were synthetically prepared for application in soft radiometal theranostics. The chelator, a [16]aneS_4_ crown thioether macrocycle, has the potential to chelate a large number of soft radiometals, including isotopes of rhodium, palladium and platinum, which are of significant interest in targeted radionuclide therapy. A number of functional handles were attached to the chelator, including an NHS ester, isothiocyanate (NCS), azide and carboxylic acid. For all of which the overall yield was moderately improved in respect to previous procedures, 4% in seven steps, 5% in eight steps, 6% in eight steps and 11% in five steps, respectively. In addition, attempts were made to shorten the synthetic preparation of these bifunctional chelators, resulting in a novel carbon–carbon linked derivative. Both the [16]aneS_4_-NHS and the [16]aneS_4_-NCS were successfully conjugated to octreotide, a mimic of natural somatostatin, as an initial proof-of-concept for further bio-conjugation studies.

## Figures and Tables

**Table 1:** Overview of selected reported metal [16]aneS_4_–ligand complexes (MLC) and their potential application in radionuclide diagnostics or therapy, see SM for a more detailed overview. Both reported radionuclides and relevant unreported radioisotopes of the same element are included.

Entry	Chelator	Metal	Isotopes	(Potential) Application ^a^	Ref.
1	[16]aneS_4_, [16]aneS_4_-ol, [16]aneS_4_-diol	Rh	^103^Rh, ^103m^Rh, ^105^Rh	MLC, TRT	[26,27,29]
2	[16]aneS_4_-ol	RhX	X = ^211^At, ^131^I, ^123^I, ^124^I	MLC, TRT, SPECT, PET	[28]
3	[16]aneS_4_	Cu	^60^Cu, ^64^Cu, ^67^Cu	MLC, PET, TRT, SPECT	[19,32,34,35]
4	[16]aneS_4_	Pt	^191^Pt, ^193m^Pt, ^195m^Pt	TRT	[20,37,38]
5	[16]aneS_4_-3-octyoxyl	Pb	^103^Pd	MLC, TRT	[21]
6	[16]aneS_4_	Sb	^119^Sb	TRT	[20,39]
7	[16]aneS_4_	Tc	^94m^Tc, ^99m^Tc	TRT, PET, SPECT	[19,20,40]

^a^ MLC = metal–ligand complex, TRT = targeted radiotherapy, PET = positron emission tomography, SPECT = single-photon emission computed tomography.

## Supplementary Materials

The following are available online, Table S1: Overview of reported and potential applications of [16]aneS4 chelator-metal complexes and derivatives. Figure S1: Structures of commonly used [16]aneS4 chelator derivatives., Figure S2: Compound overview used in the macrocyclization screening between DHLA-derivatives (14, 16 and 18) and electrophiles (2, 5, 6 and 19), Table S2: Overview of screening.

## Sample Availability

Samples of the compounds are available from the authors.
