# Supplementary material for: Novel Bifunctional [16]aneS4-Derived Chelators for Soft Radiometals
Source: Molecules. 2021 Jul 29;26(15):4603. doi: 10.3390/molecules26154603 (PMC8348053; doi:10.3390/molecules26154603)
Supplement: Supplementary file 1 [file molecules-26-04603-s001.zip › molecules-1309093-supplementary.pdf]

Supporting information

Novel Bifunctional [16]aneS_4-derived Chelators for Soft Radiometals

Natan J.W. Straathof,[1] Charlotte B. Magnus,[1] Fedor Zhuravlev,[1] Andreas I. Jensen[1]*

[1]Center for Nuclear Technologies (DTU Nutech), Technical University of Denmark, Frederiksborgvej 399, DK-4000 Roskilde, Denmark.

Table of Contents

General considerations....................3

Literature overview of [16]aneS_4-metal complexes....................4

Compound preparation....................7

Ring-closure screening....................29

Bio-conjugation of 9 and 13 with Octreotide....................34

References....................38

NMR spectra....................39

General considerations

All reagents and solvents were bought from commercial suppliers; Sigma-Aldrich, ABCR Chemicals, FluoroChem or TCI Chemicals, and were used as received. Technical solvents were bought from VWR International and used as received. Solvents were distilled when necessary, following the procedures according to the method of Grubbs. Organic solutions were concentrated under reduced pressure on a Büchi rotary evaporator using a water bath. Flash column chromatography was performed by using Geduran Silica gel Si 60 (40-63 μm). Thin layer chromatography (TLC) was carried out on commercially pre-coated aluminum sheets (Merck Silica gel 60) and visualized using fluorescence quenching or relevant stain (e.g. $KMnO_4$, or iodine). ^{1}H-NMR or ^{13}C-NMR spectra were recorded on a 400 MHz Bruker instrument using relevant deuterated solvents (e.g. $CDCl_3$ or DMSO-d_6). Coupling constants are given in Hz. ^{1}H-NMR signals are reported in chemical shift (δ ppm), multiplicities are reported as; s = singlet, d = doublet, t = triplet, q = quartet, p = pentet, h = hextet, hept = heptet, m = multiplet, b = broad. ^{13}C-NMR signals are reported in chemical shift (δ ppm). Low-resolution mass spectrometry was measured on a Shimadzu MS-2020 with an electro spray ionization spectrometer (LC-ESI) or matrix-assisted laser desorption/ionization, coupled to a time-of-flight mass spectrometer (MALDI-TOF).

Literature overview of [16]aneS_4-metal complexes

The table below shows which (radio)metals were chelated – metal-ligand complex (MLC) – with a [16]aneS4 chelator or derivative thereof. In addition, the known radioisotopes are grouped together, showing which has been utilized, or which have potential utilization in conjunction with a bifunctional [16]aneS4 chelator, e.g. within therapy or imaging. Unstable (radio)isotopes are indicated with an * for extra clarity. Abbreviations used in the table: MLC = metal-ligand complex, TRT = targeted radionuclide therapy (e.g. alpha, beta or Auger electron radiotherapy), PET = positron emission tomography, SPECT = single-photon emission computed tomography.

Table S1. Overview of reported and potential applications of [16]aneS_4 chelator-metal complexes and derivatives.

Entry	Isotope[a]	Metal salt[b]	Chelator[c]	Reported	Potential	Ref.
1	^{103}Rh	$RhCl_2$	[16]aneS_4	MLC	-	24,25
2	^{123}I*	Rh(Cl)I	-	-	TRT, SPECT	20
3	^{124}I	Rh(Cl)I	-	-	PET	20
4	^{125}I*	Rh(Cl)I	-	-	TRT, µSPECT	20
5	^{131}I*	Rh(Cl)I	[16]aneS_4-ol	MLC	TRT, SPECT	26
6	^{211}At*	Rh(Cl)At	[16]aneS_4-ol	MLC	TRT	26
7	^{103}Rh	$RhCl_3$	[16]aneS_4	MLC	-	24,25
8	^{103m}Rh*	$RhCl_2$	[16]aneS_4-ol	MLC	TRT	27
9	^{105}Rh*	$RhCl_2$	[16]aneS_4-diol	MLC	TRT	27
10	^{59}Co	$CoCl_4$	[16]aneS_4	MLC		24
11	^{55}Co*	$CoCl_2$	-	-	PET	19
12	^{63}Cu	$Cu(ClO_4)_2$	[16]aneS_4	MLC	-	31
13	^{63}Cu	$Cu(MeCN)_4PF_6$	[16]aneS_4-2,2,8,8-tetrakis	MLC	-	32
14	^{60}Cu*	$CuCl_2$	-	-	PET	19

15	^{61}Cu*	$CuCl_2$	-	-	PET	19
16	^{64}Cu*	$CuCl_2$	-	-	PET, TRT	19
17	^{67}Cu*	$CuCl_2$	-	-	TRT, SPECT	33
18	^{69}Ga	$GaCl_3$	[16]aneS_4	MLC	-	34
19	^{66}Ga*	$GaCl_3$	-	-	PET	19
20	^{67}Ga*	$GaCl_3$	-	-	TRT	20
21	Pt§	$PtCl_2$	[16]aneS_4	MLC	-	35,36
22	^{191}Pt*	$PtCl_2$	-	-	TRT	20
23	^{193m}Pt*	$PtCl_2$	-	-	TRT	20
24	^{195m}Pt*	$PtCl_2$	-	-	TRT	20
25	^{103}Pd*	$PdCl_2$	[16]aneS_4	MLC	TRT	21
26	^{121}Sb, ^{123}Sb	$SbCl_3$	[16]aneS_4	MLC	-	37
27	^{119}Sb*	$SbCl_3$	-	-	TRT	20
28	^{99}Tc*	$TcNCl_4$	[16]aneS_4-diol	MLC	-	38
29	^{94m}Tc*	(TcO_4^-)	-	-	PET, SPECT	19
30	^{99m}Tc*	(TcO_4^-)	-	-	TRT, SPECT	20
31	^{58}Ni†	$NiCl_2$, $NiBr_2$, NiI_2, $Ni(OH_2)_6(BF_4)_2$	[16]aneS_4	MLC	-	1,2††
32	^{52}Cr	$CrCl_3$, $CrCl_3$, $Cr(BF_4)_3$	[16]aneS_4	MLC	-	3††
33	^{56}Fe	$FeBr_2$, FeI_2	[16]aneS_4	MLC	-	4††
34	Mo‡	$[MoCl_2(CO)_4]_2$	Me_8-[16]aneS_4 & [16]aneS_4	MLC	-	5††
35	Ru⸸	$RuCl_2$	Me_8-[16]aneS_4	MLC	-	6,7††
36	Hg¥	$Hg(ClO_4)_2$	[16]aneS_4	MLC	-	8††
37	^{199}Hg	$Hg(ClO_4)_2$	[16]aneS_4	MLC	-	8††
38	Cd$	$Cd(ClO_4)_2$	[16]aneS_4	MLC	-	9††
39	^{113}Cd	$Cd(ClO_4)_2$	[16]aneS_4	MLC	-	9††

[a]Main stable isotopes listed (>50% abundancy), unless stated otherwise. Unstable (radio)isotopes are indicated with an * for extra clarity. [b]Most common production salt. [c]See Figure S1 below for structures. §Platinum stable isotopes: ^{192}Pt, ^{194}Pt, ^{195}Pt, ^{196}Pt, ^{198}Pt. †Nickel stable isotopes: ^{58}Ni, ^{60}Ni, ^{61}Ni, ^{62}Ni, ^{64}Ni. ‡Molybdenum stable isotopes: ^{92}Mo, ^{94}Mo, ^{95}Mo, ^{96}Mo, ^{97}Mo, ^{98}Mo, ^{100}Mo. ⸸Ruthenium stable isotopes: ^{96}Ru, ^{98}Ru, ^{99}Ru, ^{100}Ru, ^{101}Ru, ^{102}Ru, ^{104}Ru. ¥Mercury stable isotopes: ^{198}Hg, ^{199}Hg, ^{200}Hg, ^{201}Hg, ^{202}Hg, ^{204}Hg. $Cadmium stable isotopes: ^{106}Cd, ^{108}Cd, ^{110}Cd, ^{111}Cd, ^{112}Cd, ^{114}Cd. ††References in Supporting Information.

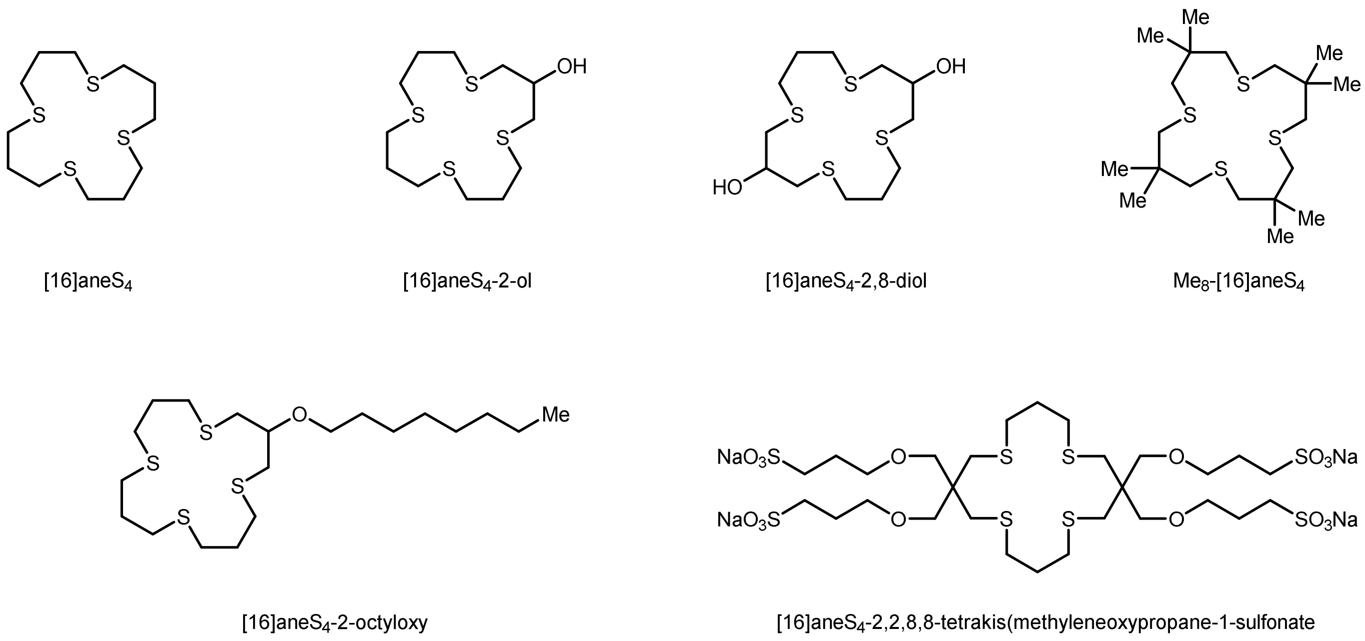

Figure S1. Structures of commonly used [16]aneS_4 chelator derivatives.

Compound preparation

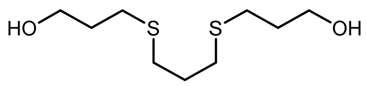

3,3'-(propane-1,3-diylbis(sulfanediyl))bis(propan-1-ol) (1). Compound **1** was prepared by a modified version of the synthesis proposed by Edema *et al.*[1]. Propane-1,3-dithiol (5.4 g, 108 mmol), 3-chloro-1-propanol (9.5 mg, 94.5 mmol, 2 eq.) and Cs_2CO_3 (33.4 g, 325.8 mmol, 2 eq.) and DMF (100 mL) was added to a flame dried flask. The resulting mixture was stirred under argon at 60 °C for 3 hours, then diluted with EtOAc, filtered and concentrated. The crude was transferred to a separation funnel containing ethyl acetate (200 mL) and water (200 mL). The aqueous phase was discarded and the organic phase was washed twice with water (200 mL), dried over Na_2SO_4 and rotary evaporated to dryness. The crude product was further purified by flash chromatography with hexane/ethyl acetate (2:8) to yield an off-white wax (8.5 g, 76%).

TLC (80% EtOAc in Hexane) Rf = 0.36.

^{1}H NMR (400 MHz, Chloroform-d) δ 3.75 (t, *J* = 6.1 Hz, 4H), 2.64 (t, *J* = 7.1 Hz, 8H), 1.86 (dt, *J* = 13.0, 7.1 Hz, 8H).

^{13}C NMR (101 MHz, Chloroform-d) δ 61.90, 32.11, 31.04, 29.28, 28.96..

MS (LC-ESI) calculated for $C_9H_{21}O_2S_2$ [M+H] 225.092, found 247.230 [M+Na].

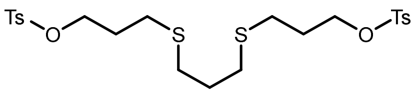

(propane-1,3-diylbis(sulfanediyl))bis(propane-3,1-diyl) bis(4-methylbenzenesulfonate) (2). Compound **1** (8.5 g, 38 mmol), tosylchloride (18.3 g, 114 mmol, 3 eq.) pyridine (21 g, 266 mmol, 7 eq.) and DCM (100 mL) was added to a flame dried flask. The resulting mixture was stirred at room temperature for 3 hours and followed by TLC then 100 mL saturated aq. NH_4Cl was added and vigorously stirred for 15 minutes. The crude was added to a separation funnel containing hexane (200 mL) and ethyl acetate (200 mL) and water (400 mL). The aqueous phase was discarded and the organic phase was washed twice with aq. NaOH (2 M, 150 mL), twice with aq. HCl (1 M, 150 mL), once with water (200 mL), dried over Na_2SO_4 and rotary evaporated to dryness to yield the titled compound as a clear yellow oil (17 g, 84%).

TLC (50% EtOAc in Hexane) Rf = 0.77.

^{1}H NMR (400 MHz, $CDCl_3$) δ 7.79 (d, J = 8.4 Hz, 4H), 7.35 (d, J = 8.1 Hz, 4H), 4.13 (t, J = 6.0 Hz, 4H), 2.52 (td, J = 7.1, 1.3 Hz, 8H), 2.45 (s, 6H), 1.97 – 1.86 (m, 4H), 1.77 (p, J = 7.1 Hz, 2H).

^{13}C NMR (101 MHz, $CDCl_3$) δ 144.99, 133.17, 130.04, 128.05, 68.96, 30.92, 29.18, 29.05, 27.98, 21.79.

MS (LC-ESI) calculated for $C_{23}H_{33}O_6S_4$ [M+H] 533.109, found 555.047 [M+Na].

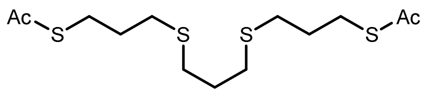

S,S'-((propane-1,3-diylbis(sulfanediyl))bis(propane-3,1-diyl)) diethanethioate (3). Compound **2** (17 g, 32 mmol) was dissolved in DMF (200 mL) and CH_3COSK (22 g, 191 mmol, 6 equiv.) was added. The mixture was stirred under argon atmosphere and analyzed by TLC (7:3, Hex:EtOAc, Rf = 0.85). The crude was added to a separation funnel containing 400 mL Hex:EtOAc and 400 mL water. The organic phase was washed twice with water (400 mL) dried over Na_2SO_4 and rotary evaporated to dryness. The crude was purified by chromatography (Hexane/EtOAc, 9:1, Rf = 0.26) to yield the product as a brown oil (6.4 g, 59%).

TLC (10% EtOAc in Hexane) Rf = 0.26.

^{1}H NMR (400 MHz, $CDCl_3$) δ 2.97 (t, J = 7.1 Hz, 4H), 2.58 (dt, J = 17.4, 7.2 Hz, 8H), 2.33 (s, 6H), 1.85 (pd, J = 7.1, 5.5 Hz, 6H).

^{13}C NMR (101 MHz, $CDCl_3$) δ 195.74, 31.02, 30.95, 30.78, 29.52, 29.35, 28.16.

MS (LC-ESI) calculated for $C_{13}H_{25}O_2S_4$ [M+H] 341.074, found 363.138 [M+Na].

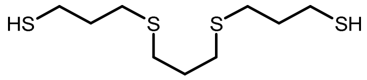

3,3'-(propane-1,3-diylbis(sulfanediyl))bis(propane-1-thiol) (4). Compound **3** (6.4 g, 18.8 mmol) was dissolved in degassed methanol (80 mL). Acetyl chloride (1.25 mL, 16 mmol, 0.85 eq.) was added dropwise. The mixture was stirred under argon at 40 °C and followed by TLC (100% Hexane, Rf = 0.68) until completion. The crude was added to a separation funnel containing Hex:EtOAc (150 mL, 1:1) and washed with water and brine. The organic phase was separated and dried with Na_2SO_4, filtered and concentrated under reduced pressure to yield the desired product as a clear oil (4.3 g, 68%).

TLC (100% Hexane) Rf = 0.68.

^{1}H NMR (400 MHz, $CDCl_3$) δ 2.63 (pd, *J* = 7.0, 4.2 Hz, 12H), 1.88 (h, *J* = 7.0 Hz, 6H), 1.37 (t, *J* = 8.0 Hz, 2H).

^{13}C NMR (101 MHz, $CDCl_3$) δ 33.46, 31.02, 30.54, 29.42, 23.56.

MS (LC-ESI) calculated for $C_9H_{21}S_4$ [M+H] 257.053, found 279.035 [M+Na].

Note: discoloration to dark pinkish can occur if not stored under inert atmosphere (e.g. Argon) and in the dark.

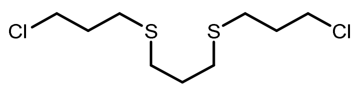

1,3-bis((3-chloropropyl)thio)propane (5). Prepared following according to an adapted literature procedure.[11] **WARNING: 1,3-bis((3-chloropropyl)thio)propane (5) is a mustard analogue and should be handled with extreme care and kept from moisture as much as possible.** To a stirring solution of 3,3'-(propane-1,3-diylbis(sulfanediyl))bis(propan-1-ol) (**1**) (450 mg, 2.1 mmol), in DCM (20 mL, 0.1 M) at 0 °C under argon, was added a solution of diluted SO_2Cl_2 (665 mg, 5.0 mmol, 2.5 equiv.) in DCM (5 mL) dropwise within 5 minutes. The solution was then stirred for 6 hours at room temperature. After completion, the mixture was concentrated *in vacuo* to remove excess SO_2Cl_2 and solvent. The title compound was obtained as a yellow liquid with a strong sulfuric odor and used without further purification (88% yield).

TLC (100% Hexane) Rf = 0.75.

^{1}H NMR (400 MHz, $CDCl_3$) δ 3.65 (t, J = 6.3 Hz, 1H), 2.64 (dt, J = 17.5, 7.1 Hz, 2H), 2.03 (p, J = 6.6 Hz, 1H), 1.87 (p, J = 7.1 Hz, 1H).

^{13}C NMR (101 MHz, $CDCl_3$) δ 43.62, 32.30, 30.99, 29.30, 29.13.

Note: discoloration can occur after prolonged storage (2 months) under ambient conditions.

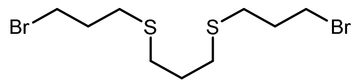

1,3-bis((3-bromopropyl)thio)propane (6). Procedure was adapted from a literature procedure.[12] **WARNING: 1,3-bis((3-bromopropyl)thio)propane (6) is a mustard analogue and should be handled with extreme care and kept from moisture as much as possible.** A mixture of trimethylene sulfide (10.7 g, 61 mmol), 1,3-dibromopropane (6.2 g, 31 mmol), potassium bromide (5.1 g, 1 mmol), and powdered potassium carbonate (200 mg, 1.5 mmol), in MeCN (35 mL) was stirred under argon in a sealed, foil-wrapped flask for 5 days at 65 ºC (additional MeCN was added if the solvent level went low). After this, the crude was concentrated to dryness and further purified with SiO_2 chromatography (gradient; 100% Hexane to 10% EtOAc in Hexane). Title compound was obtained as a white liquid with slight sulfuric odor (75% yield).

TLC (10% EtOAc in Hexane) Rf = 0.84.

^{1}H NMR (400 MHz, $CDCl_3$) δ 3.52 (t, *J* = 6.4 Hz, 2H), 2.67 (t, *J* = 7.0 Hz, 2H), 2.12 (p, *J* = 6.7 Hz, 2H).

^{13}C NMR (101 MHz, $CDCl_3$) δ 32.35, 32.19, 32.16, 32.14, 30.15.

Note: discoloration can occur after prolonged storage (2 weeks) under ambient conditions.

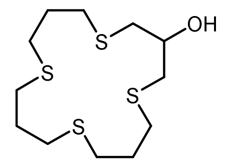

1,5,9,13-tetrathiacyclohexadecan-3-ol (7). Compound **7** was prepared as described by Li *et al.* [2]. A three horizontal-necked round-bottomed flask was filled with dry DMF (450 mL) and metal free Cs_2CO_3 (12.2 g, 37 mmol, 2.2 eq.). The solution was heated to 80 °C and kept under argon. Compound **4** (4.3 g, 17mmol) and 1,3-dichloro-2-propanol (21.9 g, 17 mmol, 1 eq.) was dissolved in DMF and placed in an addition funnel. This mixture was added slowly over 10 hours under vigorously stirring (2-3 drops per minute). The reaction was followed by TLC (15% EtOAc in toluene, Rf = 0.39). The crude was filtered and concentrated under reduced pressure to a yield a turbid, orange oil. The crude was purified by chromatography (Hexane/EtOAc, 75:25, Rf= 0.33) to yield product **5** as a white wax in excellent purity (2.4 g, 45%).

TLC (15% EtOAc in Toluene) Rf = 0.39

^{1}H NMR (400 MHz, $CDCl_3$) δ 3.87 (ttd, J = 7.2, 5.1, 3.0 Hz, 1H), 2.88 (d, J = 3.0 Hz, 1H, OH), 2.84 (dd, J = 13.5, 5.1 Hz, 2H), 2.75 (dt, J = 13.5, 7.2 Hz, 2H), 2.71 – 2.61 (m, 12H), 1.91 (dp, J = 9.7, 7.0 Hz, 6H).

^{13}C NMR (101 MHz, $CDCl_3$) δ 68.94, 38.31, 31.06, 30.97, 30.91, 29.81, 29.52.

MS (LC-ESI) calculated for $C_{12}H_{25}OS_4$ [M+H] 313.0788, found 335.155 [M+Na].

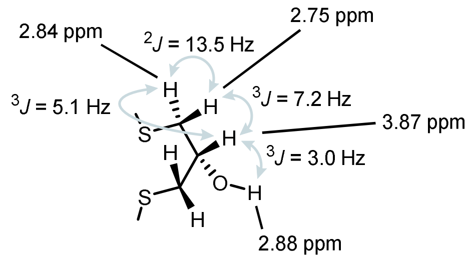

Observed ^{1}H-NMR of 1,5,9,13-tetrathiacyclohexadecan-3-ol 7 (zoom at C^1, C^2, C^3 and OH).

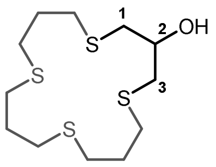

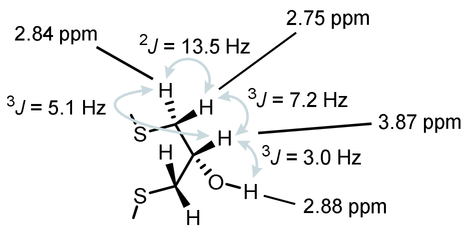

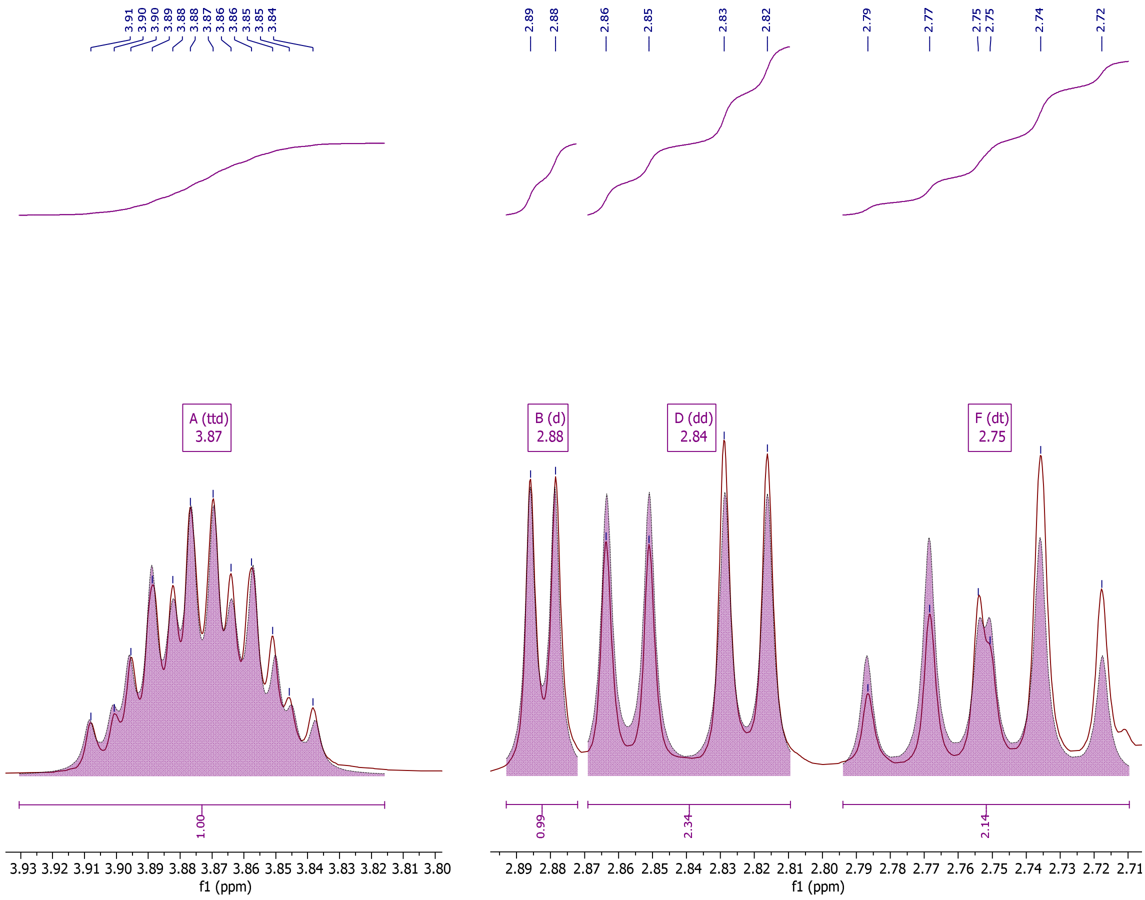

Reference ^{1}H-NMR of 1,5,9,13-tetrathiacyclohexadecane-3,11-diol (zoom at C^{1}, C^{2}, C^{3} and OH).

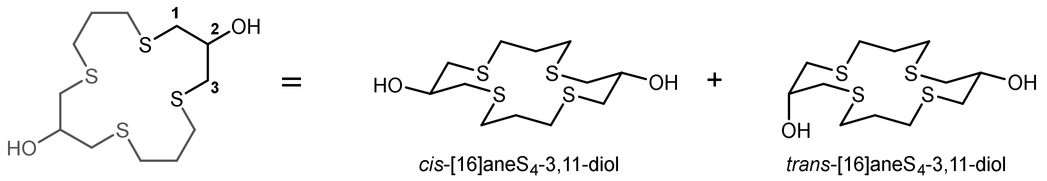

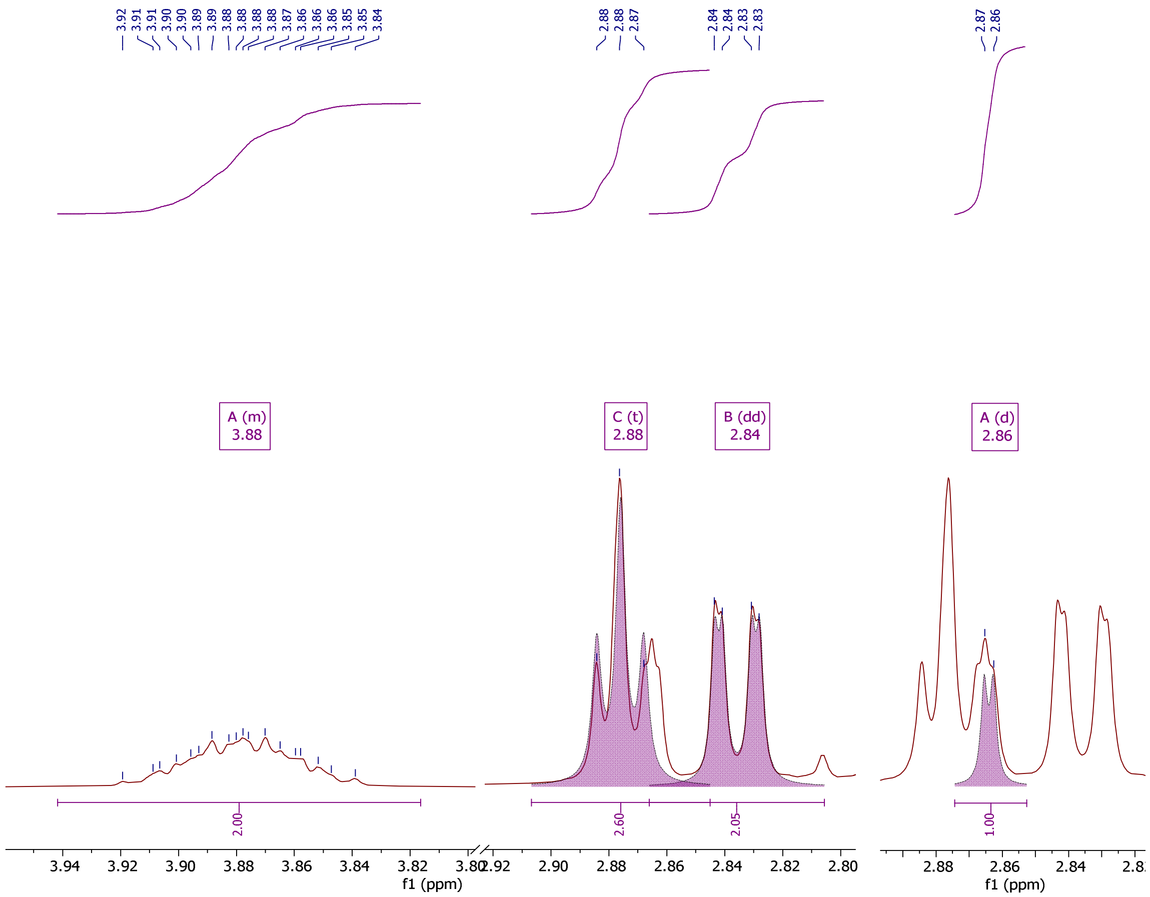

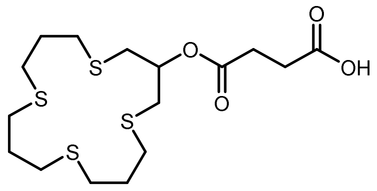

S,S'-((propane-1,3-diylbis(sulfanediyl))bis(ethane-2,1-diyl)) diethanethioate (16aneS$_4$-oxobutanoic acid) (8). 16aneS$_4$-3-oxo-butanoic acid was synthesized inspired by the approach by Li *et al.*[3]. Compound **7** (200 mg, 0.64 mmol), succinic anhydride (131 mg, 1.31 mmol, 2.05 equiv.) and triethylamine (0.45 mL, 3.20 mmol, 5 equiv.) was dissolved in 5 mL DMF and heated to 80°C. The reaction was followed by TLC (6:4:0.1, hexane/EtOAc/AcOH). After completion, the mixture was concentrated by removing DMF under vacuum. The remaining crude oil was dissolved and separated in ethyl acetate (50 mL) and 0.1M HCl (50mL). The organic phase was washed with 0.1 M HCl three times (50 mL) before being dried over Na_2SO_4, filtered and rotary evaporated to dryness to yield a brown oil. The crude was purified by chromatography (30% EtOAc in Hexane + 0.1% AcOH) to yield the desired product (195 mg, 73%) as an off-white semi solid.

TLC (40% EtOAc in Hexane + 0.1% AcOH) Rf = 0.33

^{1}H NMR (400 MHz, Chloroform-d) δ 5.09 (p, J = 6.3 Hz, 1H), 2.90 (dd, J = 13.9, 6.0 Hz, 2H), 2.79 – 2.63 (m, 19H), 1.92 (dp, J = 8.1, 7.0 Hz, 6H).

^{13}C NMR (101 MHz, $CDCl_3$) δ 177.89, 171.71, 72.77, 34.89, 31.43, 30.90, 30.74, 29.74, 29.66, 29.11, 29.04.

MS (LC-ESI) calculated for $C_{16}H_{29}O_4S_4$ [M+H] 413.09487. Found 435.155 [M+Na].

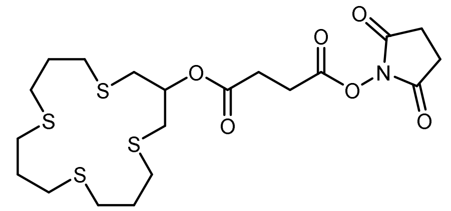

1,5,9,13-tetrathiacyclohexadecan-3-yl (2,5-dioxopyrrolidin-1-yl) succinate (9). Compound **8** (42 mg, 0.1 mmol) and DMAP (14 mg, 0.12 mmol, 1.2 equiv.) was dissolved in 1.0 mL dry DCM. The solution was cooled in an ice bath and dicyclohexylcarbodiimine (DCC) (29.4 mg, 0.18 mmol, 1.8 equiv.) was added portion wise. N-hydroxysuccinimide (15.7 mg, 0.15 mmol, 1.5 equiv.) was added portion wise, and the solution turned milky white immediately. The reaction mixture was allowed to stir to room temperature and followed by TLC until full consumption of the starting material. The crude was filtered and concentrated under reduced pressure to give a colorless oil. The crude was further purified by column chromatography (40% EtOAc in Hexane + 0.1% AcOH) to yield the titled compound as an off-white wax (30 mg, 52%).

TLC (40% EtOAc in Hexane + 0.1% AcOH) Rf = 0.38

^{1}H NMR (400 MHz, $CDCl_3$) δ 5.03 (q, J = 6.3 Hz, 1H), 2.91 (t, J = 7.0 Hz, 2H), 2.83 (dd, J = 14.0, 6.0 Hz, 2H), 2.77 (s, 4H), 2.75 – 2.55 (m, 17H), 1.85 (h, J = 7.1 Hz, 7H).

^{13}C NMR (101 MHz, $CDCl_3$) δ 169.99, 168.95, 167.08, 77.48, 77.16, 76.84, 73.01, 34.87, 31.40, 30.96, 30.77, 29.79, 29.67, 29.03, 26.52, 25.73.

MS (LC-ESI) calculated for $C_{20}H_{32}NO_6S_4$ [M+H] 510,11, found 532.18 [M+Na].

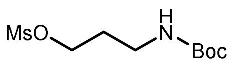

3-((tert-butoxycarbonyl)amino)propyl methanesulfonate (10). N-Boc-propanol (1.5 g, 8.6 mmol) and triethylamine (1.7 g, 17 mmol, 2 equiv.) was dissolved in anhydrous DCM (10 mL) and cooled in an ice bath, before adding mesylate chloride (2.9 g, 26 mmol, 3 eq.). The reaction mixture was then stirred under argon at 40 °C. The reaction was followed by TLC (6:4, Hex:EtOAc, Rf = 0.28). The crude was extracted in 50 mL EtOAc and 50 mL sat. NH_4Cl. The organic layer was washed once with NH_4Cl (50 mL) and twice with brine (50 mL), dried with Na_2SO_4, filtered and concentrated to yield a brown solid. The crude was purified by chromatography (40% EtOAc in hexane) to yield an off-white solid (1.3 g, 60%).

TLC (40% EtOAc in hexane) Rf = 0.25.

^{1}H NMR (400 MHz, $CDCl_3$) δ 4.88 (s, 1H), 4.28 (t, *J* = 6.0 Hz, 2H), 3.25 (t, *J* = 6.5 Hz, 2H), 3.02 (s, 3H), 1.93 (p, *J* = 6.2 Hz, 2H), 1.43 (s, 9H).

^{13}C NMR (101 MHz, $CDCl_3$) δ 156.21, 67.50, 67.17, 37.50, 29.78, 28.49.

MS (LC-ESI) calculated for $C_9H_{20}NO_5S$ [M+H] 254.106, found 276.219 [M+Na].

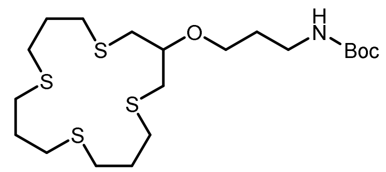

Tert-butyl (3-((1,5,9,13-tetrathiacyclo hexadecan-3-yl)oxy)propyl)carbamate (11). Compound **6** (250 mg, 0.8 mmol) and NaH (48 mg, 1.2 mmol, 1.5 equiv.) was dissolved in DMF:toluene (6:2, v/v, 1.0 mL). The mixture was stirred under argon at room temperature for 30 minutes. Compound **7** was added and the mixture was heated to 60 ºC. The reaction was followed by TLC. The crude was separated between EtOAc and water. The organic phase was washed twice with water, dried with Na_2SO_4, filtered and concentrated to yield an orange oil. The crude was further purified by chromatography (gradient; 5% EtOAc in Hexane to 25% EtOAc in Hexane) to give the title compound as an off-white solid (78%).

TLC (50% DCM in Hexane) Rf = 0.56, (10% MeOH in DCM) Rf = 0.88.

^{1}H NMR (400 MHz, $CDCl_3$) δ 5.05 (bs, 1H, NH), 3.86 (ttd, *J* = 7.5, 4.9, 2.3 Hz, 1H), 3.57 (dt, *J* = 16.2, 5.8 Hz, 2H), 3.23 (q, *J* = 6.3 Hz, 1H), 2.94 – 2.58 (m, 16H), 1.90 (dp, *J* = 9.7, 7.1 Hz, 6H), 1.74 (p, *J* = 6.1 Hz, 2H), 1.43 (s, 9H).

^{13}C NMR (101 MHz, $CDCl_3$) δ 156.20, 79.08, 68.94, 68.03, 38.28, 35.54, 31.67, 31.03, 30.95, 30.88, 29.78, 29.69, 29.49, 28.59.

MS (MALDI-TOF) calculated for $C_{20}H_{40}NO_3S_4$ [M+H] 470.189, found 492.204 [M+Na].

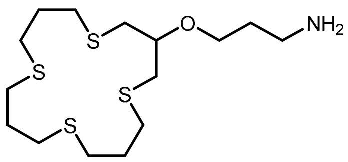

3-((1,5,9,13-tetrathiacyclohexadecan-3-yl)oxy)propan-1-amine (12). Compound **11**, tert-butyl (3-((1,5,9,13-tetrathiacyclohexadecan-3-yl)oxy)propyl)carbamate (235 mg, 0.5 mmol), was dissolved in DCM (10 mL) and then treated with TFA (5 mL) at room temperature and stirred for 1 hour. After complete consumption of the starting material, the reaction mixture was concentrated *in vacuo* to remove excess TFA, and further purified with silica gel chromatography (10% MeOH in DCM). The title compound was obtained as an off-white solid (88% yield).

TLC (10% MeOH in DCM) Rf = 0.22 – 0.15, (10% MeOH in DCM + 0.1 % TEA) Rf = 0.30.

^{1}H NMR (400 MHz, $CDCl_3$) δ 3.70 (t, *J* = 5.4 Hz, 2H), 3.58 (p, *J* = 6.5, 6.1 Hz, 1H), 3.11 (t, *J* = 6.2 Hz, 2H), 2.85 – 2.70 (m, 5H), 2.66 (dt, *J* = 23.0, 7.4 Hz, 14H), 1.98 – 1.86 (m, 6H), 1.85 (d, *J* = 6.8 Hz, 2H), 1.21 (bs, 2H, NH_2).

^{13}C NMR (101 MHz, $CDCl_3$) δ 78.99, 68.58, 39.13, 35.05, 31.23, 30.72, 30.21, 29.45, 29.39, 26.90.

MS (LC-ESI) calculated for $C_{15}H_{32}NOS_4$ [M+H] 370,137, found 370.450 [M+H].

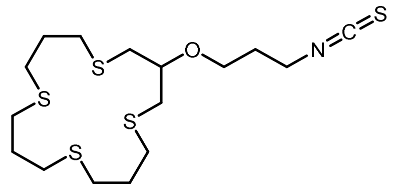

3-(3-isothiocyanatopropoxy)-1,5,9,13-tetrathiacyclohexadecane (13). Following a literature procedure.[13] 1, 1-thiocarbonyldiimidazole (213 mg, 1.2 mmol, 1.2 equiv.) was dissolved in dry DMF (5 mL) at 50 ºC. To this mixture, a solution of 3-((1,5,9,13-tetrathiacyclohexadecan-3-yl)oxy)propan-1-amine (369 mg, 1 mmol, 1 equiv.) and Et_3N (1.37 mL, 1 mmol, 1 equiv.) in DMF (5 mL) was added dropwise. The mixture was stirred at 50 ºC, and the progress monitored by TLC (1% MeOH in DCM). After complete consumption of the starting material, the reaction was cooled down, aqueous LiCl was added, and extracted with EtOAc three times. The combined organic phases were collected and dried over $MgSO_4$, filtered, and concentrated *in vacuo*. Purification by flash column chromatography (15% EtOAc in Hexane) was performed to obtain the title compound as an off-white grease (65% yield).

TLC (50% EtOAc in Hexane) Rf = 0.66.

^{1}H NMR (400 MHz, $CDCl_3$) δ 3.74 – 3.69 (m, 2H), 3.67 (t, *J* = 5.9 Hz, 2H), 3.59 (p, *J* = 5.9 Hz, 1H), 2.79 (dd, *J* = 14.4, 5.9 Hz, 2H), 2.75 – 2.62 (m, 11H), 2.01 – 1.85 (m, 8H).

^{13}C NMR (101 MHz, $CDCl_3$) δ 129.76, 79.44, 65.82, 42.13, 35.58, 31.70, 30.88, 30.84, 30.51, 29.84, 29.70.

MS (LC-ESI) calculated for $C_{16}H_{30}NOS_5$ [M+H] 412.093. Found 443.150 [M+H+MeOH], 465.170 [M+Na+MeOH].

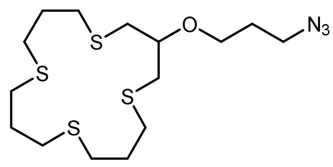

3-(3-azidopropoxy)-1,5,9,13-tetrathiacyclohexadecane (23). Following a two-step-one-pot procedure adapted from literature.[14] NaN_3 (5.0 g, 77 mmol) was placed in a flask with a stirring bar. The flask was evacuated with nitrogen, dry EtOAc (77 mL) was added, and cooled down to 0 ºC. Sulfuryl chloride (6.2 mL, 77 mmol) was added dropwise slowly under rigorous stirring, and the mixture was slowly warmed to room temperature and then stirred for 18 h. The suspension was cooled down to 0 °C. Imidazole (10.0 g, 146 mmol) was added portion-wise. The suspension was stirred for 3 h at 0 ºC and then basified with sat. $NaHCO_3$ in H_2O (150 mL). The organic layer was washed with water, separated and dried over $MgSO_4$. This solution was filtered and cooled to 0 ºC and placed under a nitrogen atmosphere, and conc. H_2SO_4 (4.1 mL, 77 mmol) was added dropwise slowly. The mixture was gradually warmed up to room temperature under vigorous stirring. After 30 min, a precipitate had formed. This was collected, washed with EtOAc and dried briefly under high vacuum and used directly without further purification. A second flask was prepared with a stirring bar, 3-((1,5,9,13-tetrathiacyclohexadecan-3-yl)oxy)propan-1-amine **12** (37 mg, 0.1 mmol) in MeOH and H_2O (5:1, v/v, 10 mL) and cooled down to 0 ºC. To this reaction mixture was added, N-imidazole-1-sulfonyl azide (as prepared above, 24 mg, 0.15 mmol, 1.5 equiv.), Cu(II)SO_4 (10 mol%) and K_2CO_3 (27 mg, 2 mmol, 2 equiv.). The mixture was allowed to stir at 0 ºC for 1 hour, and then warmed up to room temperature and stirred for another 5 hours. The reaction was monitored by TLC analysis (50% EtOAc in hexane). After completion, the reaction mixture was diluted with 1 M HCl, and the mixture was extracted with EtOAc three times. The combined organic layers were washed with sat. $NaHCO_3$ in H_2O and brine, dried over MgSO4, and concentrated *in vacuo*. The product was further purified with a short plug of SiO2 to afford the title compound as an off-white wax (75% yield).

TLC (50% EtOAc in Hexane) Rf = 0.45.

^{1}H NMR (400 MHz, $CDCl_3$) δ. 3.58 (p, J = 6.5, 6.1 Hz, 1H), 3.11 (t, J = 6.2 Hz, 2H), 2.77 (dd, J = 16.9, 6.0 Hz, 4H), 2.65 (m, 15H), 1.87 (q, J = 6.8 Hz, 5H), 1.22 (m, 2H).

^{13}C NMR (101 MHz, $CDCl_3$) δ 79.54, 65.92, 42.23, 35.68, 31.80, 30.98, 30.61, 29.94, 29.80.

MS (LC-ESI) calculated for $C_{15}H_{30}N_3OS_4$ [M+H] 396.127. Found 418.150.

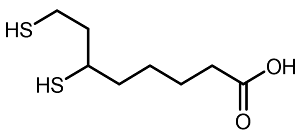

6,8-dimercaptooctanoic acid, *di*-hydrolipoic acid (DHLA) (14). Following a literature procedure.[10] In a round bottom flask, equipped with a stirring bar was added, lipoic acid (1 g, 5 mmol) in EtOH (50 mL) and $NaHCO_3$ in H_2O (0.25 M, 50 mL). To this mixture was added, $NaBH_4$ (0.4 g, 10 mmol, 2 equiv.) portion wise and the mixture was stirred for 3 hour at room temperature. The crude was then taken up into EtOAc and extracted with 1 M HCl. The combined organic layers were dried over MgSO4 and concentrated *in vacuo* to give a colorless oil as the title compound (0.99 g, 99% yield).

Obtained spectral data were consistent with known literature values.

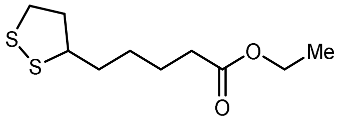

Ethyl 4-(1,2-dithiolan-3-yl)butanoate (15). A flask equipped with a stirring bar was charged with lipoic acid (1.03 g, 5 mmol, 1 equiv.) in DCM (20). The solution was cooled down to 0 °C and added was dicyclohexylcarbodiimine (DCC) (2.1 g, 10 mmol, 2 equiv.) and ethanol (10 mL). The mixture was stirred at 0 ºC for 15 min, then allowed to warm up to room temperature and stirred for another 18 hours. Hereafter, the reaction mixture was quenched with 1 M HCl and extracted with EtOAc. The combined organic layers were dried over $MgSO_4$ and concentrated *in vacuo*. The crude product was further purified by running it over a plug of SiO_2 with DCM and collected as a yellow oil (75% yield).

TLC (DCM) Rf = 0.63.

^{1}H NMR (400 MHz, $CDCl_3$) δ 4.11 (q, *J* = 7.1 Hz, 2H), 3.56 (dq, *J* = 8.4, 6.4 Hz, 1H), 3.22 – 3.05 (m, 2H), 2.45 (ddd, *J* = 12.9, 12.0, 6.6 Hz, 1H), 2.29 (td, *J* = 7.5, 2.7 Hz, 2H), 1.89 (dq, *J* = 12.6, 6.9 Hz, 1H), 1.66 (dtdd, *J* = 16.7, 11.1, 7.9, 6.1 Hz, 2H), 1.46 (dddd, *J* = 15.7, 10.4, 5.1, 2.9 Hz, 1H), 1.24 (t, *J* = 7.1 Hz, 3H).

^{13}C NMR (101 MHz, CDC_3) δ 173.51, 60.28, 56.28, 40.20, 38.47, 34.59, 34.10, 28.75, 24.67, 14.22.

MS (LC-ESI) calculated for $C_{10}H_{19}O_2S_2$ [M+H] 235.08265. Found

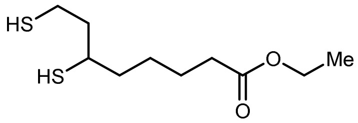

Ethyl 5,7-dimercaptoheptanoate (16). In a round bottom flask, equipped with a stirring bar was added, **15** (1.17 g, 5 mmol) in EtOH (50 mL) and $NaHCO_3$ in H_2O (0.25 M, 50 mL). To this mixture was added, $NaBH_4$ (0.4 g, 10 mmol, 2 equiv.) portion wise and the mixture was stirred for 3 hour at room temperature. The crude was then taken up into EtOAc and extracted with 1 M HCl. The combined organic layers were dried over $MgSO_4$ and concentrated *in vacuo* to give a colorless oil as the title compound (98% yield).

TLC (DCM) Rf = 0.61.

^{1}H NMR (400 MHz, $CDCl_3$) δ 4.11 (q, *J* = 7.2 Hz, 2H), 2.90 (ddt, *J* = 12.1, 7.4, 3.8 Hz, 1H), 2.79 – 2.58 (m, 2H), 2.30 (t, *J* = 7.3 Hz, 2H), 1.89 (dtd, *J* = 13.9, 7.8, 4.4 Hz, 1H), 1.74 (dddd, *J* = 13.9, 8.9, 5.7, 2.3 Hz, 1H), 1.68 – 1.58 (m, 2H), 1.56 – 1.38 (m, 1H), 1.33 (t, *J* = 8.0 Hz, 1H), 1.28 (d, *J* = 7.6 Hz, 1H), 1.24 (t, *J* = 7.1 Hz, 3H).

^{13}C NMR (101 MHz, $CDCl_3$) δ 173.55, 60.27, 42.73, 39.30, 38.72, 34.15, 26.51, 24.58, 22.28, 14.27.

MS (LC-ESI) calculated for $C_{10}H_{21}O_2S_2$ [M+H] 237.098. Found 259.150.

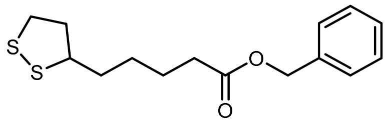

Benzyl 5-(1,2-dithiolan-3-yl)pentanoate (17). A flask equipped with a stirring bar was charged with lipoic acid (1.03 g, 5 mmol, 1 equiv.) in DCM (20 mL). The solution was cooled down to 0 °C and dicyclohexylcarbodiimide (DCC) was added (2.1 g, 10 mmol, 2 equiv.) and benzyl alcohol (2 mL). The mixture was stirred at 0 C for 15 min, then allowed to warm up to room temperature and stirred for another 18 hours. Hereafter, the reaction mixture was quenched with 1 M HCl and extracted with EtOAc. The combined organic layers were dried over $MgSO_4$ and concentrated *in vacuo*. The crude product was further purified by running it over a plug of SiO_2 with DCM and collected as a yellow oil (82% yield).

TLC (20% EtOAc in Hexane) Rf = 0.50.

^{1}H NMR (400 MHz, $CDCl_3$) δ 7.41 – 7.27 (m, 5H), 5.11 (s, 2H), 3.54 (dq, *J* = 8.3, 6.4 Hz, 1H), 3.22 – 3.04 (m, 2H), 2.43 (dtd, *J* = 12.9, 6.6, 5.4 Hz, 1H), 2.37 (t, *J* = 7.4 Hz, 2H), 1.88 (dq, *J* = 12.8, 6.9 Hz, 1H), 1.77 – 1.58 (m, 4H), 1.56 – 1.36 (m, 2H).

^{13}C NMR (101 MHz, $CDCl_3$) δ 173.39, 136.13, 128.97, 128.62, 128.38, 128.04, 66.29, 56.55, 56.26, 40.42, 38.58, 34.68, 34.49, 34.18, 28.82, 24.78.

MS (LC-ESI) calculated for $C_{15}H_{21}O_2S_2$ [M+H] 297.098. Found 319.150.

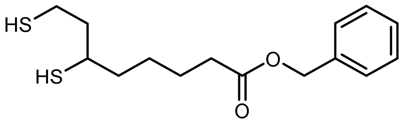

Benzyl 6,8-dimercaptooctanoate (18). In a round bottom flask, equipped with a stirring bar was added, **15** (1.49 g, 5 mmol) in EtOH (50 mL) and $NaHCO_3$ in H_2O (0.25 M, 50 mL). To this mixture was added, $NaBH_4$ (0.4 g, 10 mmol, 2 equiv.) portion wise and the mixture was stirred for 3 hour at room temperature. The crude was then taken up into EtOAc and extracted with 1 M HCl. The combined organic layers were dried over $MgSO_4$ and concentrated *in vacuo* to give a colorless oil as the title compound (92% yield) and used directly.

TLC (20% EtOAc in Hexane) Rf = 0.48

^{1}H NMR (400 MHz, $CDCl_3$) δ 7.40 – 7.25 (m, 5H), 5.10 (s, 2H), 3.54 (dq, *J* = 8.0, 6.9 Hz, 1H), 3.25 – 3.05 (m, 2H), 2.45 (dtd, *J* = 12.9, 6.9, 5.4 Hz, 1H), 2.35 (t, *J* = 7.4 Hz, 2H), 1.85 (dq, *J* = 12.9, 6.9 Hz, 1H), 1.75 – 1.55 (m, 4H), 1.58 – 1.32 (m, 2H), 1.34 (t, *J* = 8.0 Hz, 1H), 1.29 (d, *J* = 8.0 Hz, 1H),

^{13}C NMR (101 MHz, $CDCl_3$) δ 173.41, 136.17, 129.05, 128.82, 128.58, 127.12, 65.17, 56.55, 56.26, 41.01, 37.78, 35.78, 34.49, 34.25, 28.82, 25.81.

MS (LC-ESI) calculated for $C_{15}H_{23}O_2S_2$ [M+H] 299.11395, found 321.150 [M+Na].

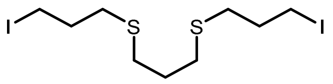

1,3-bis((3-iodopropyl)thio)propane (19). Following a literature procedure.[12] A mixture of trimethylene sulfide (10.70 g, 61.4 mmol), 1,3-diiodopropane (9.11 g, 30.8mmol), potassium iodide (5.10 g, 30.7 mmol), and powdered potassium carbonate (200 mg, 1.5 mmol)), in MeCN (150 mL) was stirred under argon in a sealed, foil-wrapped flask for 3 days at 65 ºC followed by 8 days at 45 ºC. The reaction mixture was diluted with methanol (120 mL), treated with aqueous ammonia (1M, 20 mL), and heated briefly to boiling. After this the crude mixture was concentrated to dryness. This material was stirred for 2 hours with aqueous citric acid (1 M, 100 mL) and cyclohexane (200 mL). The organic layer was separated, dried with $MgSO_4$, concentrated under reduced pressure, and rotated under high vacuum overnight to completely remove cyclohexane, giving the product **10** as a yellow solid which was used in the next step without further purification (92% yield).

^{1}H NMR (400 MHz, $CDCl_3$) δ 3.29 (td, J = 6.7, 1.6 Hz, 1H), 2.62 (td, J = 7.2, 2.4 Hz, 3H), 2.12 – 1.99 (m, 1H), 1.86 (ddd, J = 9.4, 6.0, 2.4 Hz, 1H).

^{13}C NMR (101 MHz, $CDCl_3$) δ 32.83, 32.48, 30.90, 29.26, 5.17.

Note: decomposition to an insoluble solid can occur within 7 days if not stored under an inert atmosphere (Argon), and refrigerated (-18 ºC). Moreover, 1,3-bis((3-iodopropyl)thio)propane is assumed to be photosensitive and should be handled and stored in the dark as much as practical.

Ring-closure screening

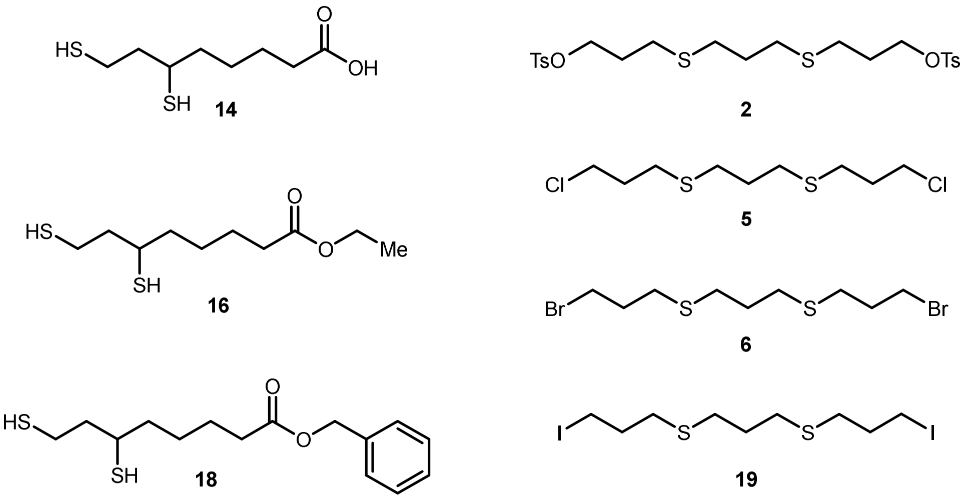

Figure S2. *Compound overview used in the macrocyclization screening between DHLA-derivatives (**14**, **16** and **18**) and electrophiles (**2**, **5**, **6** and **19**).*

Screening of different coupling partners and conditions

Conditions: Typical procedure for the macrocyclisation between dihydrolipoic acid and 1,11-dihalogen-4,8-dithiaundecane, adapted from a literature procedure.[12] A three horizontal-necked round-bottomed flask was filled with dry DMF (150 mL) and Cs_2CO_3 (12.2 g, 37 mmol, 2.2 eq.). The solution was heated to 80 ºC and kept under argon. The *bis*-Thiol (10 mmol) and bis-halogen electrophile (10 mmol) were dissolved in DMF (200 mL) and both added into an addition funnel, which was placed onto the three horizontal-necked round-bottomed flask. This mixture was added slowly (via extreme slow addition; 2-3 drops per minute, or portion wise in 10-minute intervals) to the heated reaction mixture over 6 hours under vigorously stirring. After addition, the reaction was stirred for another 6 hours. The reaction was followed by TLC analysis (2-20% EtOAc in Hexane). After this, the mixture was cooled down and the reaction mixture was filtered and concentrated under reduced pressure to a yield an off-white resedue. This residue was taken up into DCM (250 mL) and then filtered through a 4 cm diameter column containing sequential beds of sand (2 cm), Celite (2 cm) and SiO_2 (4 cm). The filter column was washed with an additional 250 mL of DCM, and the combined filtrates were concentrated under reduced pressure. The crude mixture was triturated with 5% acetone in hexane (100 mL) and further dried under reduced pressure. Further purification was performed by flash chromatography on SiO_2.

Table S2. Overview of screening.

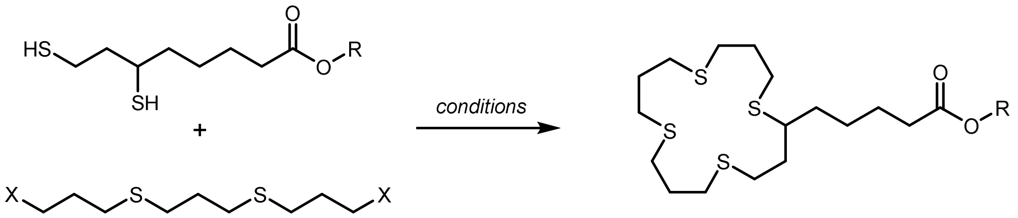

Entry[a]	R	X	Notes	Yield
1	H	I	no reaction observed	0%
2	Et	I	Reaction yielded a complex mixture	<2%[b]
3	Bn	I		15%[c]
4	Bn	Br	Low yield	3%
5	Bn	Cl		Trace
6	Bn	OTs		Trace

[a]See reagents and conditions stated above. [b]Reaction resulted in a complex mixture. [c]Instability of compound 19 resulted in low yield.

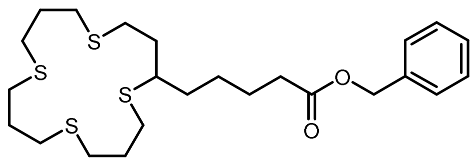

Benzyl 5-(1,5,9,13-tetrathiacyclohexadecan-2-yl)pentanoate (21).

TLC (10% EtOAc in Hexane). Rf = 0.74.

^{1}H NMR (400 MHz, cdcl$_3$) δ 7.39 – 7.30 (m, 5H), 5.11 (s, 2H), 3.65 (t, *J* = 6.3 Hz, 3H), 3.54 (dq, *J* = 8.2, 6.4 Hz, 1H), 3.21 – 3.04 (m, 2H), 2.64 (dt, *J* = 17.0, 7.1 Hz, 7H), 2.43 (ddd, *J* = 12.7, 6.5, 5.4 Hz, 1H), 2.37 (t, *J* = 7.5 Hz, 2H), 2.07 – 1.99 (m, 3H), 1.92 – 1.82 (m, 3H), 1.75 – 1.58 (m, 5H), 1.56 – 1.36 (m, 2H), 1.25 (s, 1H).

^{13}C NMR (101 MHz, cdcl$_3$) δ 173.36, 136.12, 128.95, 128.60, 128.36, 128.01, 66.26, 56.53, 56.24, 43.59, 43.57, 43.56, 40.39, 38.57, 34.66, 34.46, 34.16, 32.28, 30.95, 29.28, 29.10, 28.80, 28.59, 24.76.

MS (LC-ESI) calculated for $C_{24}H_{39}O_2S_4$ [M+H] 487.183, found 509.150 [M+Na].

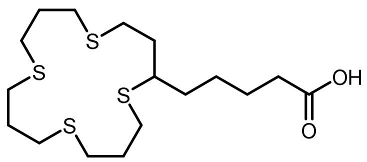

5-(1,5,9,13-tetrathiacyclohexadecan-2-yl)pentanoic acid (22). A two-neck round bottom flask was equipped with a stirring bar and charged with benzyl 5-(1,5,9,13-tetrathiacyclohexadecan-2-yl)pentanoate 21 (48 mg, 0.1 mmol), Pd/C (10 wt. %, 2 mg, 1 mol%) in EtOAc/EtOH (1:1, v/v, 2 mL). The flask was sealed with rubber septa and the mixture was degassed by evacuating the atmosphere with argon. After this, a balloon with hydrogen gas (H_2) was used to hydrogenate the mixture by addition through a long needle into the reaction mixture, under stirring for 3 hours. The reaction was briefly monitored by TLC analysis (10% EtOAc in hexane). After completion, the mixture was diluted with EtOAc and purged with argon. Hereafter the crude was filtered over Celite to remove excess residue, and washed with EtOAc. The collected mixture was concentrated and further purified over a short SiO_2 column to afford the title compound as a white solid (89% yield).

TLC (10% EtOAc in Hexane): Rf. = 0.12-0.22

^{1}H NMR (400 MHz, cdcl$_3$) δ 3.60 (t, *J* = 6.3 Hz, 3H), 3.51 (ddd, *J* = 10.2, 7.3, 4.2 Hz, 2H), 3.16 – 3.01 (m, 2H), 2.59 (dt, *J* = 17.0, 7.1 Hz, 6H), 2.40 (dtd, *J* = 13.0, 6.6, 5.4 Hz, 1H), 2.25 (t, *J* = 7.4 Hz, 2H), 1.98 (p, *J* = 6.7 Hz, 3H), 1.90 – 1.77 (m, 3H), 1.68 – 1.54 (m, 4H), 1.47 – 1.37 (m, 2H), 1.21 – 1.17 (m, 2H).

^{13}C NMR (101 MHz, cdcl$_3$) δ 176.36, 56.54, 56.23, 43.79, 43.48, 43.17, 40.48, 40.32, 39.98, 38.45, 34.60, 33.80, 32.16, 31.29, 30.82, 29.15, 28.97, 28.75, 24.61.

MS (LC-ESI) Calculated for $C_{17}H_{33}O_2S_4$ [M+H] 397.136, found 419,150 [M+Na].

Bio-conjugation of 9 and 13 with Octreotide

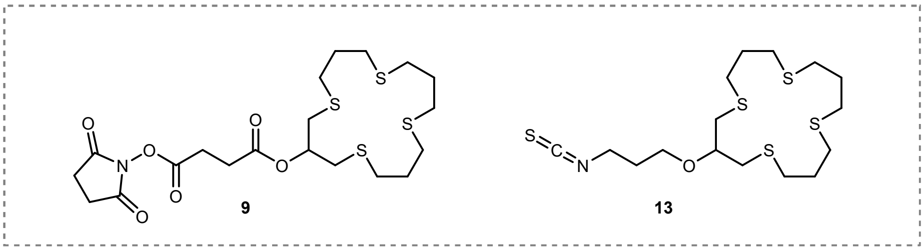

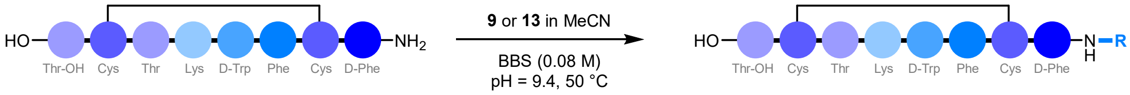

A stock solution of octreotide (2 mg, 1962 nmol) was prepared with borate buffer (2.0 mL, 0.08 M, pH = 9.4). From the octreotide stock solution was taken 200 µL (196 nmol) of solution and dispensed into an HPLC vial equipped with a stirring bar. To this vial was added freshly prepared NHS or NCS-conjugate (**9** or **13**) in MeCN (200 µL, 1.25 mmol, ~6.5 equiv.). The vial was sealed and then stirred at 50 ºC for 3.5 hours. The pH was periodically measured with a pH paper; pH should stay around 9.0, and can be adjusted by adding additional borate buffer (0.08 M, pH = 9.4). After conjugation, the mixture was directly analyzed with MALDI-TOF Mass Spectrometry.

**Entry**		**Calculated**	**Detected (m/z)**
1	Control w/o conjugate	1018.44 [M]	1042.29 $[M+H+Na]^{2+}$
2	w/ [16]aneS_4-NHS **9**	1412.52 [M]	1436.68 $[M+H+Na]^{2+}$
3	w/ [16]aneS_4-NCS **13**	1429.53 [M]	1452.72 $[M+H+Na]^{2+}$

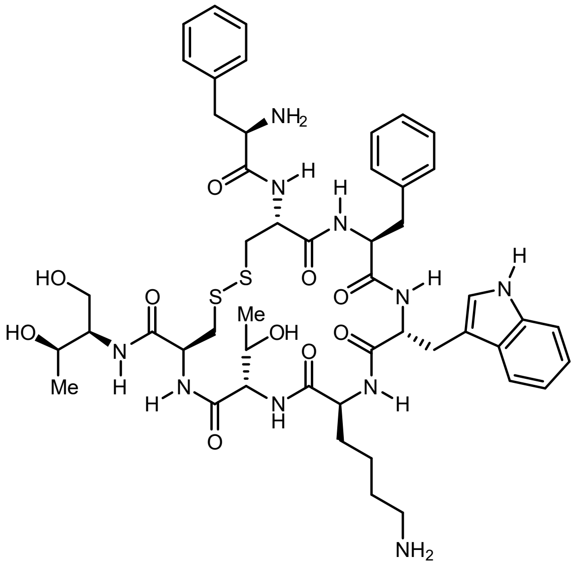

Chemical Formula: $C_{49}H_{66}N_{10}O_{10}S_2$
Exact Mass: 1018,44

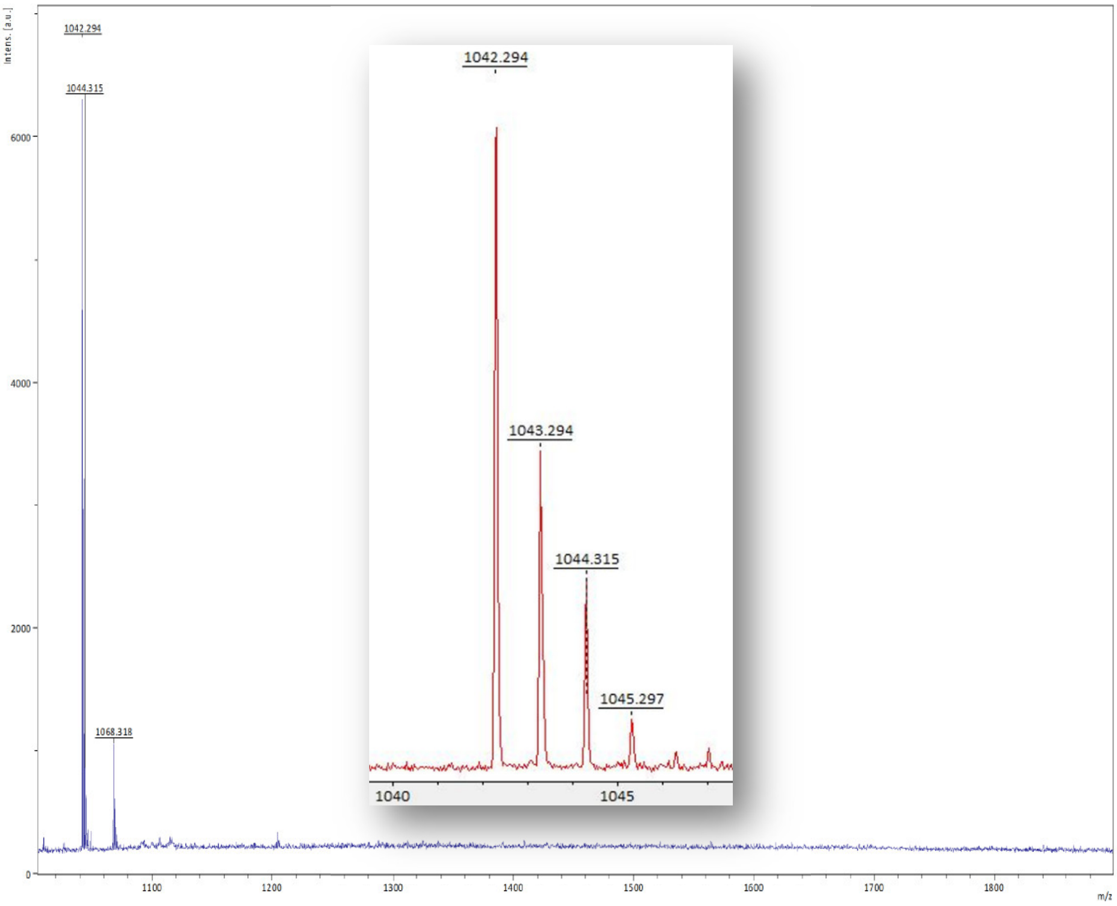

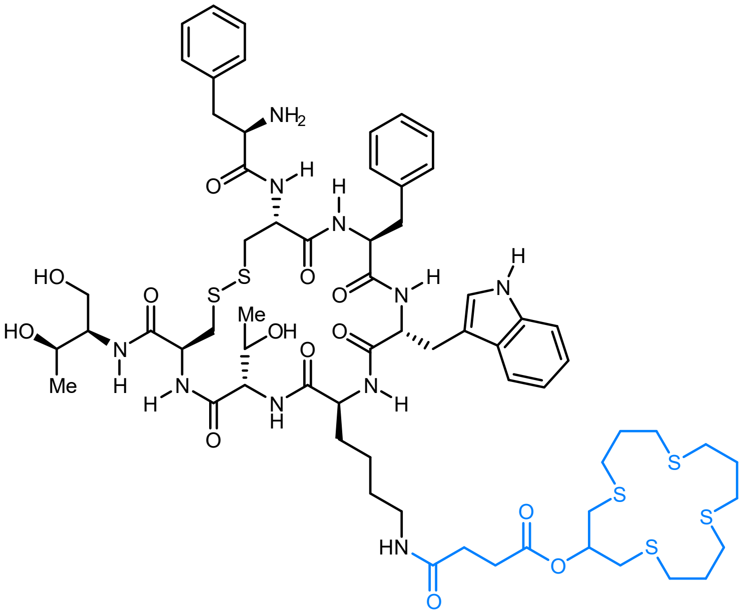

Chemical Formula: $C_{65}H_{92}N_{10}O_{13}S_6$
Exact Mass: 1412,52

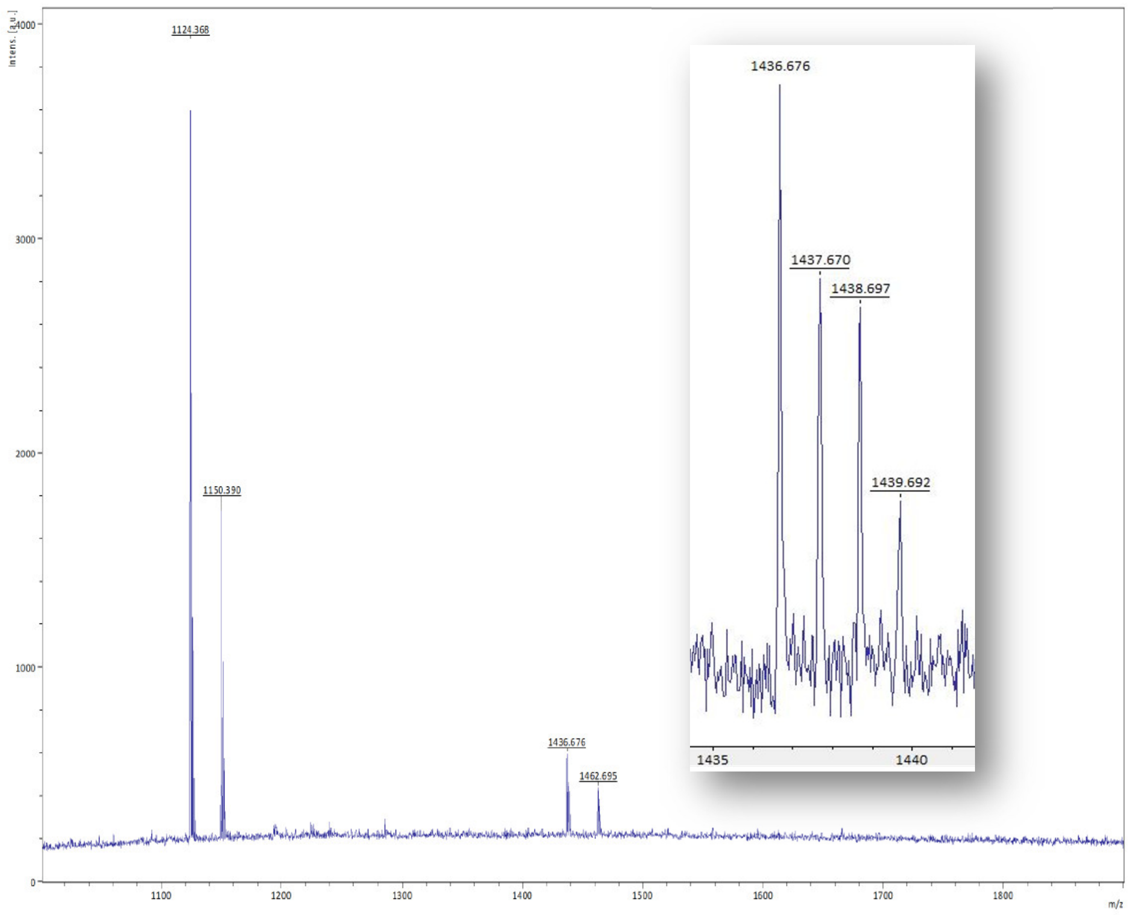

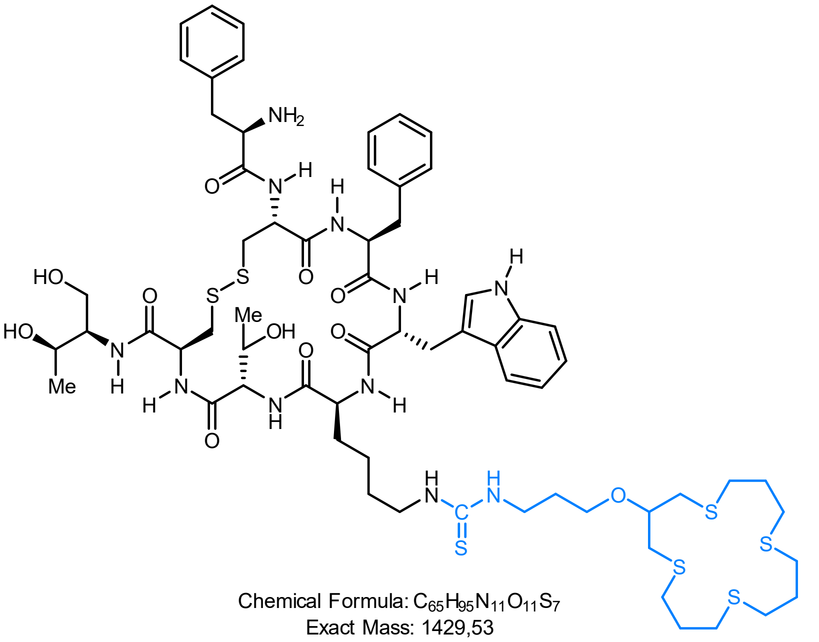

Chemical Formula: $C_{65}H_{95}N_{11}O_{11}S_7$
Exact Mass: 1429,53

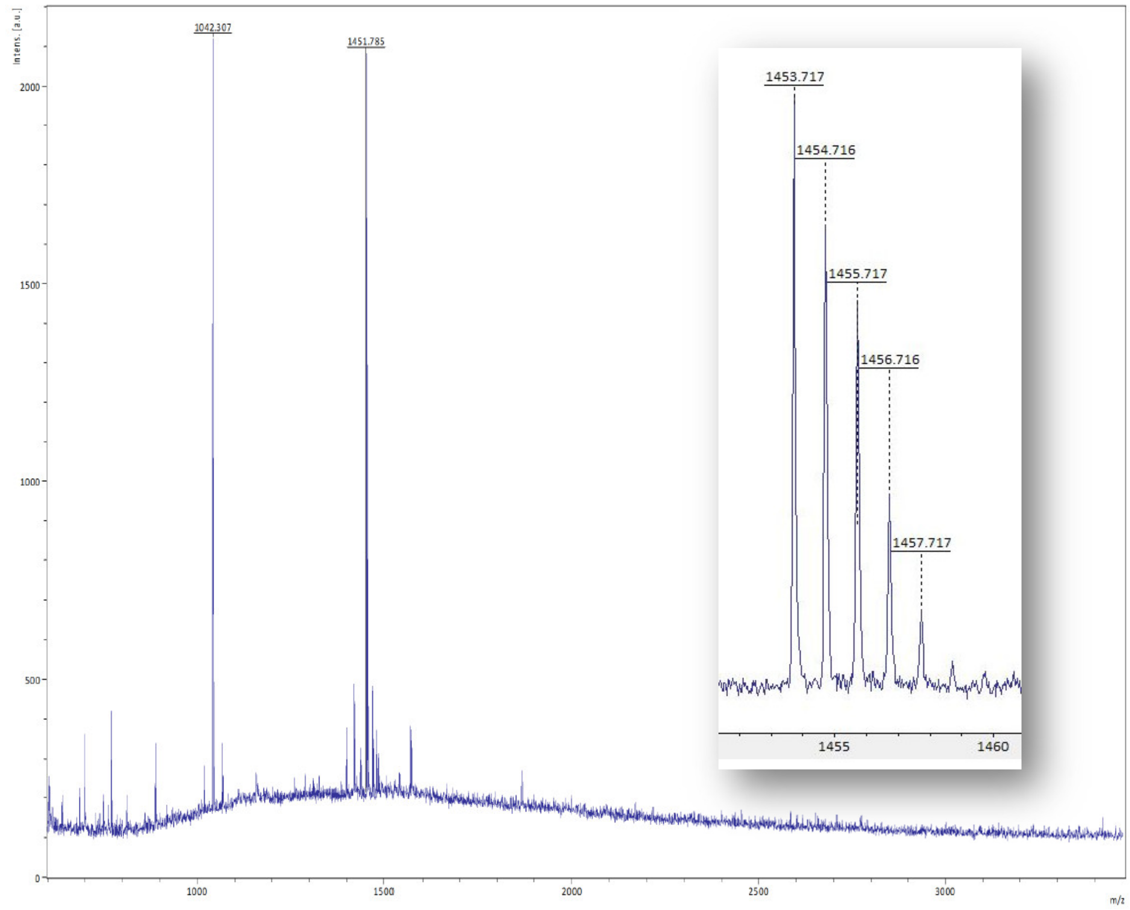

References

[1] *J. Chem. Dalton Trans.* **1998**, 2185-2189.
[2] *J. Chem. Dalton Trans.* **1992**, 2803-2806.
[3] *J. Chem. Dalton Trans.* **1997**, 1639-1644.
[4] *J. Chem. Soc. Chem. Commun.* **1989**, 1774-1775.
[5] *Angew. Chem.* **1987**, *99*(11), 1182-1183.
[6] *Inorganica Chimica Acta* **1995**, *231*, 95-101.
[7] *J. Organomet. Chem.* **1994**, *473*, 225-241.
[8] *Inorg. Chem.* **2005**, *44*, 5696-5705.
[9] *Dalton Trans.* **2006**, 3534-3543.
[10] *Tetrahedron Letters* **2010**, *51*, 5364-5367.
[11] *J. Org. Chem.* **2018**, *83*, 12432-12439.
[12] *J. Am. Chem. Soc.* **2013**, *135*, 18549-18559.
[13] *Eur. J. Med. Chem.* **2019**, *165*, 80-92.
[14] *J. Org. Chem.* **2016**, *81*, 3443−3446

NMR spectra

- Compound **1**, page S40.
- Compound **2**, page S41.
- Compound **3**, page S42.
- Compound **4**, page S43.
- Compound **5**, page S54.
- Compound **6**, page S53.
- Compound **7**, page S44.
- Compound **8**, page S45.
- Compound **9**, page S49.
- Compound **10**, page S48.
- Compound **11**, page S46.
- Compound **12**, page S47.
- Compound **13**, page S50.
- Compound **17**, page S55.
- Compound **19**, page S52.
- Compound **21**, page S56.
- Compound **22**, page S57.
- Compound **23**, page S51.

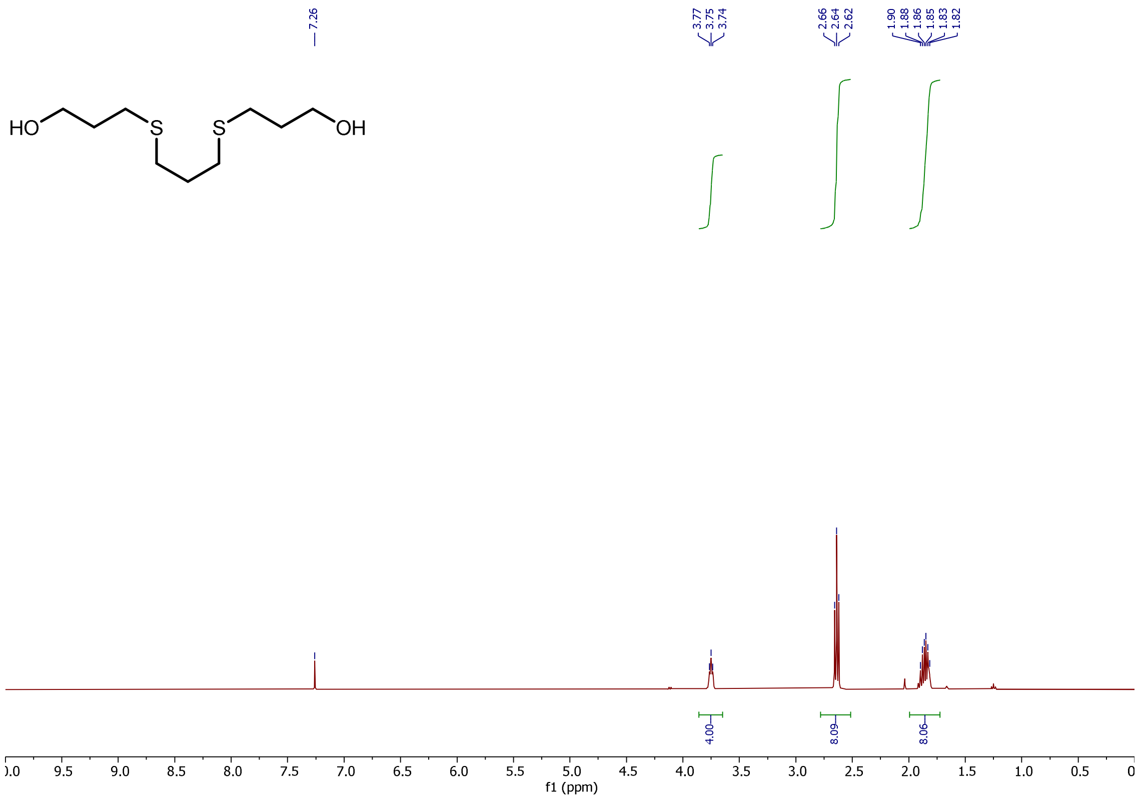

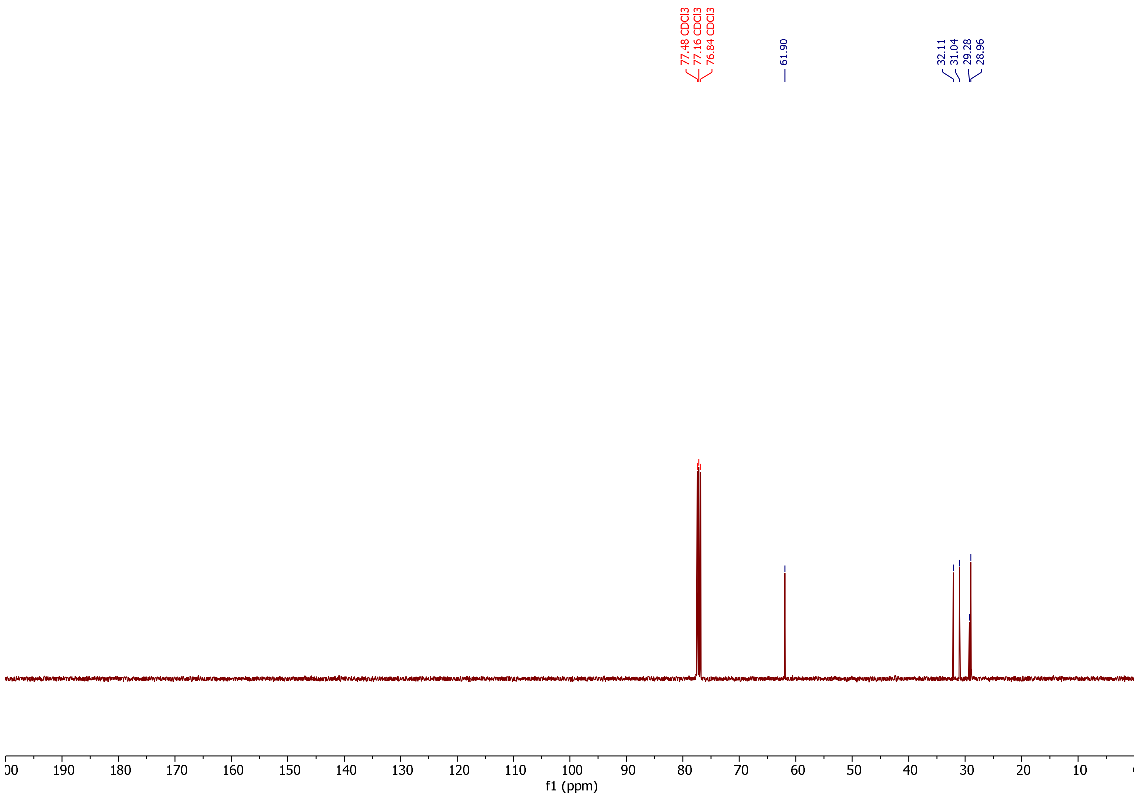

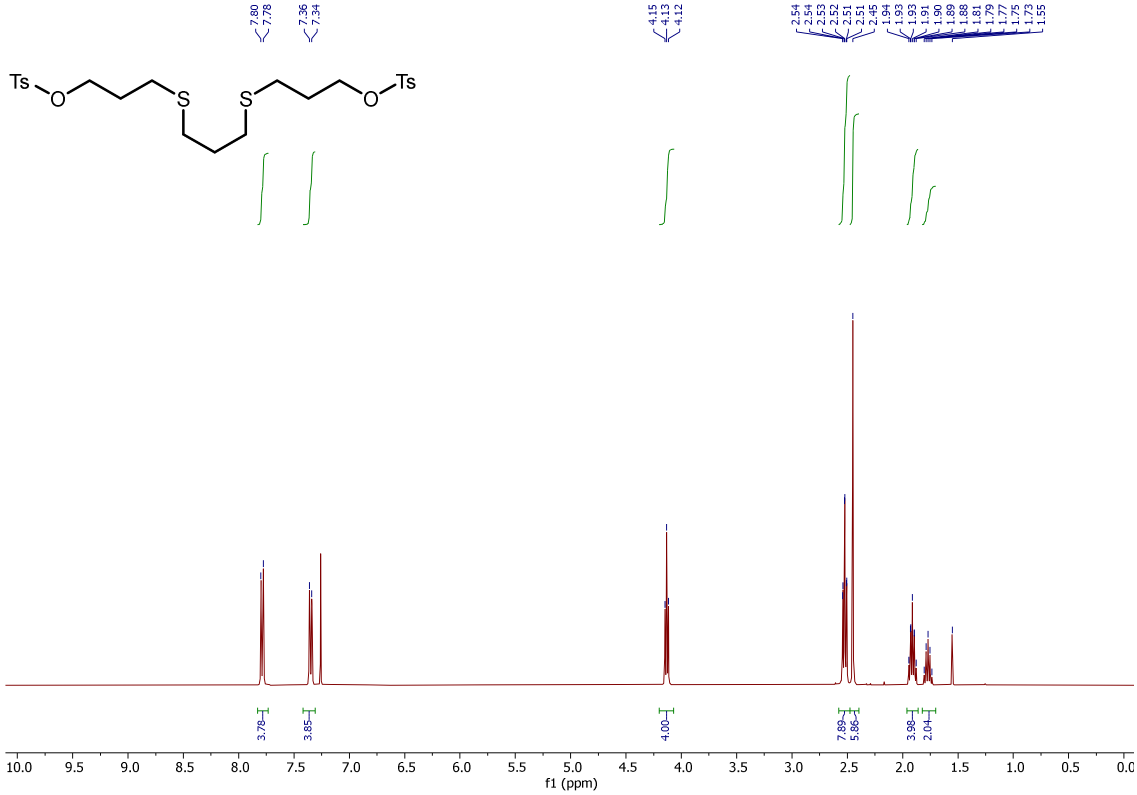
7.80
7.78
7.36
7.34
4.15
4.13
4.12
2.54
2.54
2.53
2.52
2.51
2.51
2.45
1.94
1.93
1.93
1.91
1.90
1.89
1.88
1.81
1.79
1.77
1.75
1.73
1.55
Ts
O
S
S
O
Ts
3.78
3.85
4.00
7.89
5.86
3.98
2.04
10.0 9.5 9.0 8.5 8.0 7.5 7.0 6.5 6.0 5.5 5.0 4.5 4.0 3.5 3.0 2.5 2.0 1.5 1.0 0.5 0.0
f1 (ppm)

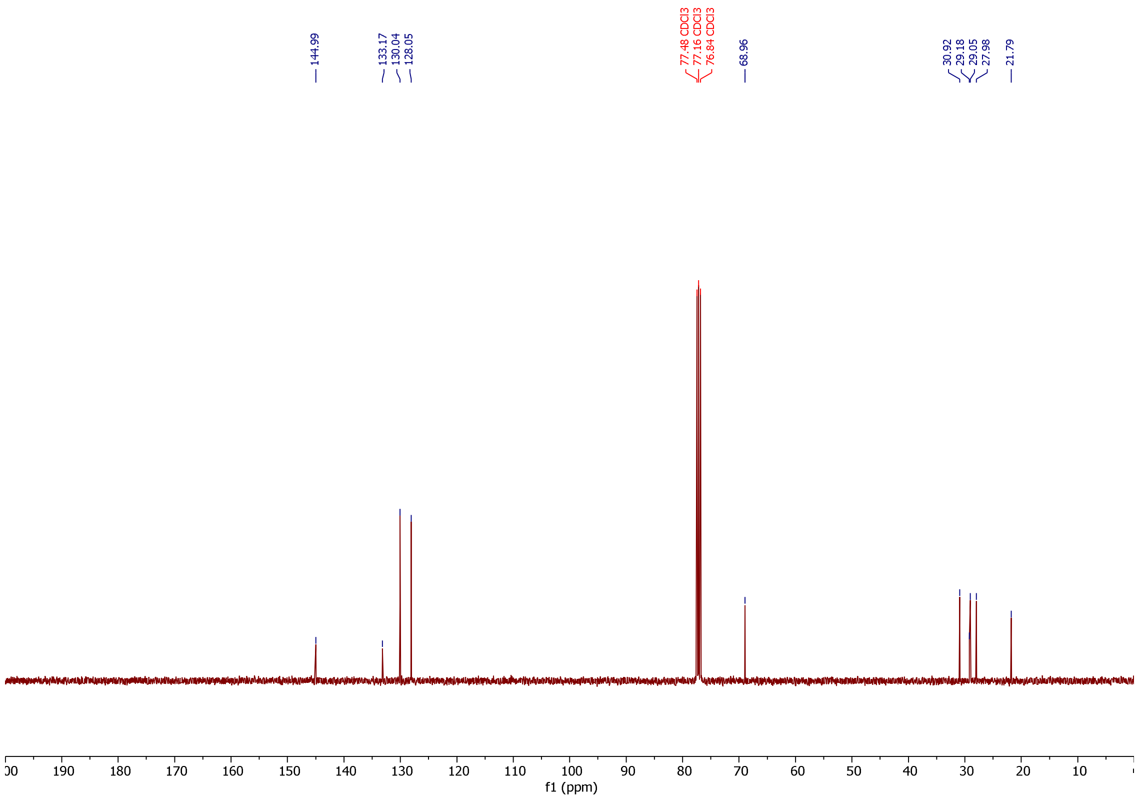
144.99
133.17
130.04
128.05
77.48 CDCl3
77.16 CDCl3
76.84 CDCl3
68.96
30.92
29.18
29.05
27.98
21.79
190 180 170 160 150 140 130 120 110 100 90 80 70 60 50 40 30 20 10
f1 (ppm)

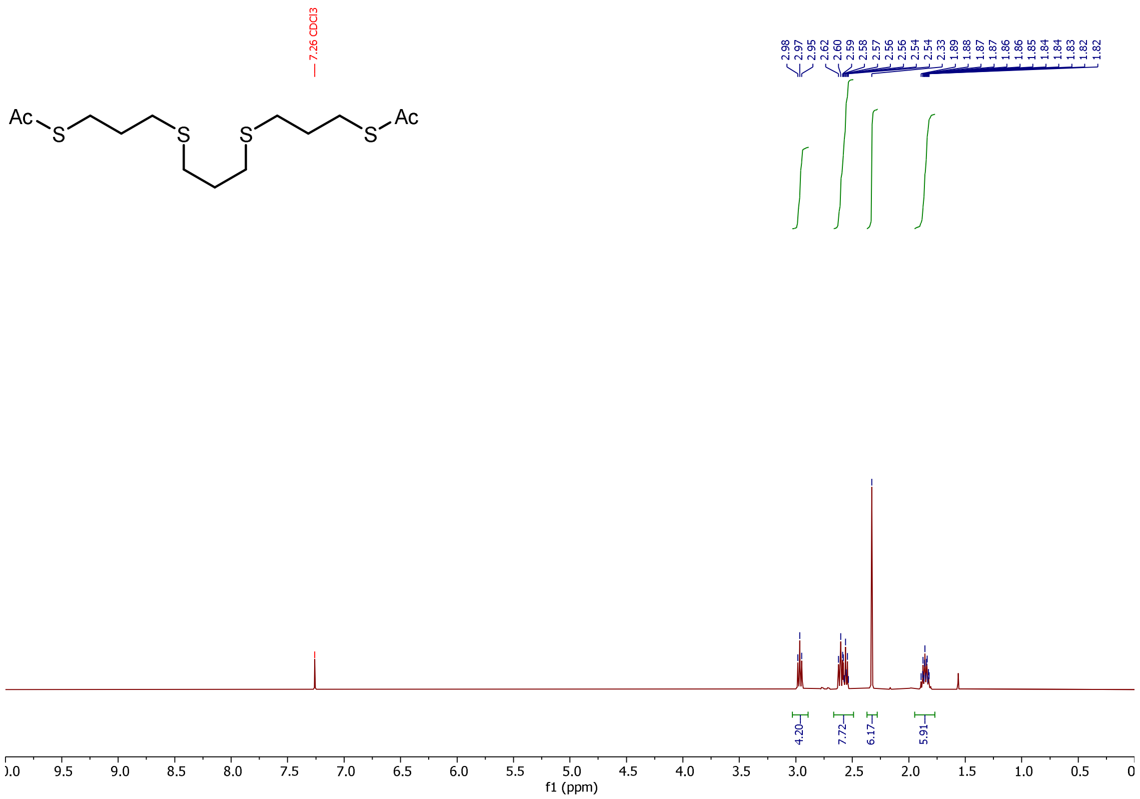
Ac
S
S
S
S
Ac
7.26 CDCl3
2.98
2.97
2.95
2.62
2.60
2.59
2.58
2.57
2.56
2.54
2.54
2.33
1.89
1.88
1.87
1.87
1.86
1.86
1.85
1.84
1.84
1.83
1.82
1.82
4.20
7.72
6.17
5.91
f1 (ppm)

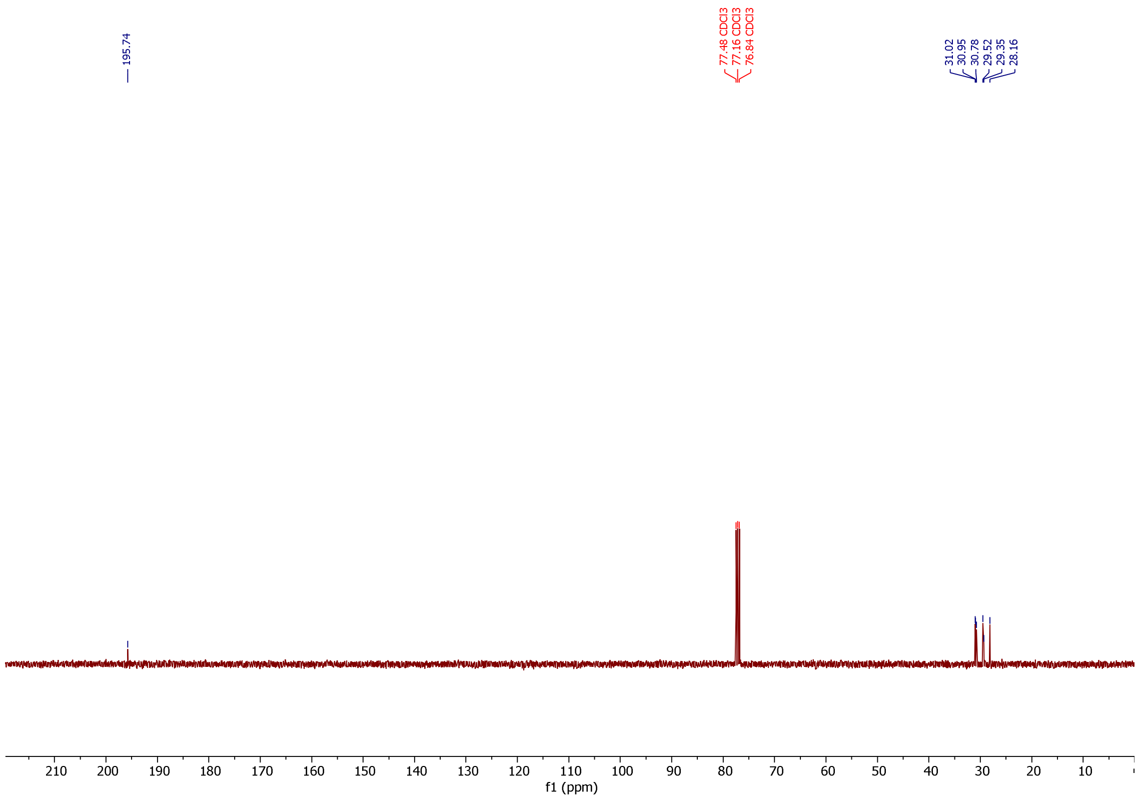
195.74
77.48 CDCl3
77.16 CDCl3
76.84 CDCl3
31.02
30.95
30.78
29.52
29.35
28.16
f1 (ppm)

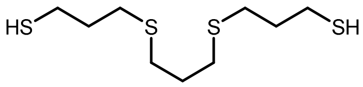
HS
S
S
SH

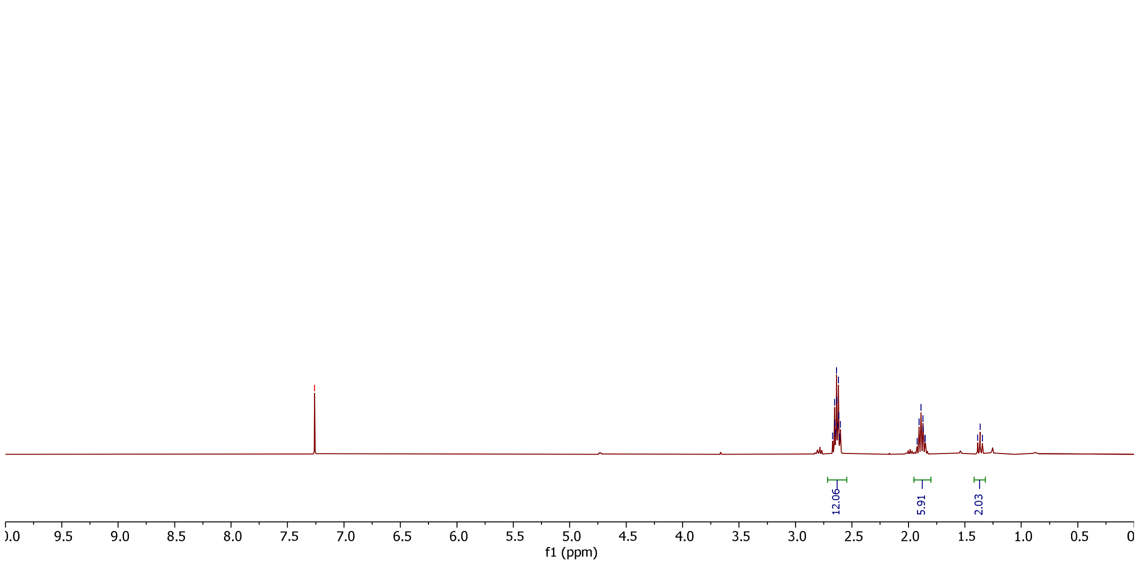
7.26 CDCl3
2.67
2.65
2.65
2.64
2.64
2.63
2.63
2.62
2.62
2.61
2.60
1.92
1.91
1.89
1.87
1.87
1.85
1.85
1.39
1.37
1.35
12.06
5.91
2.03
f1 (ppm)

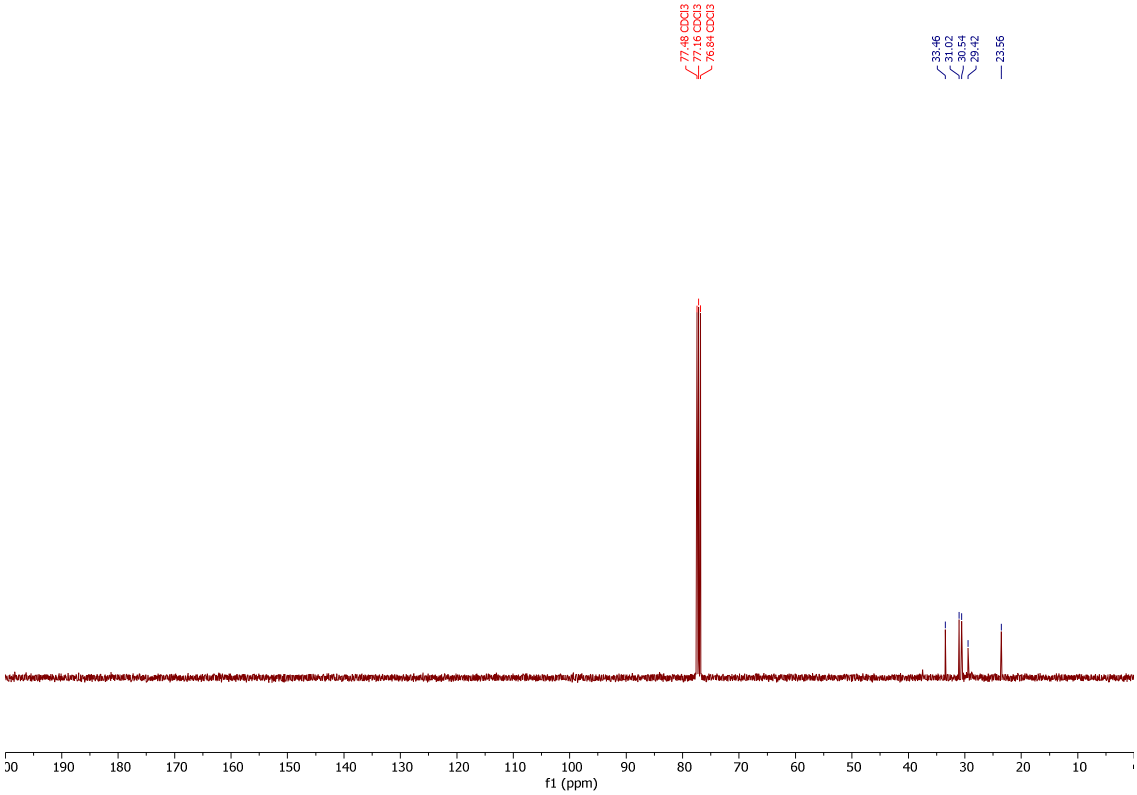
77.48 CDCl3
77.16 CDCl3
76.84 CDCl3
33.46
31.02
30.54
29.42
23.56
f1 (ppm)

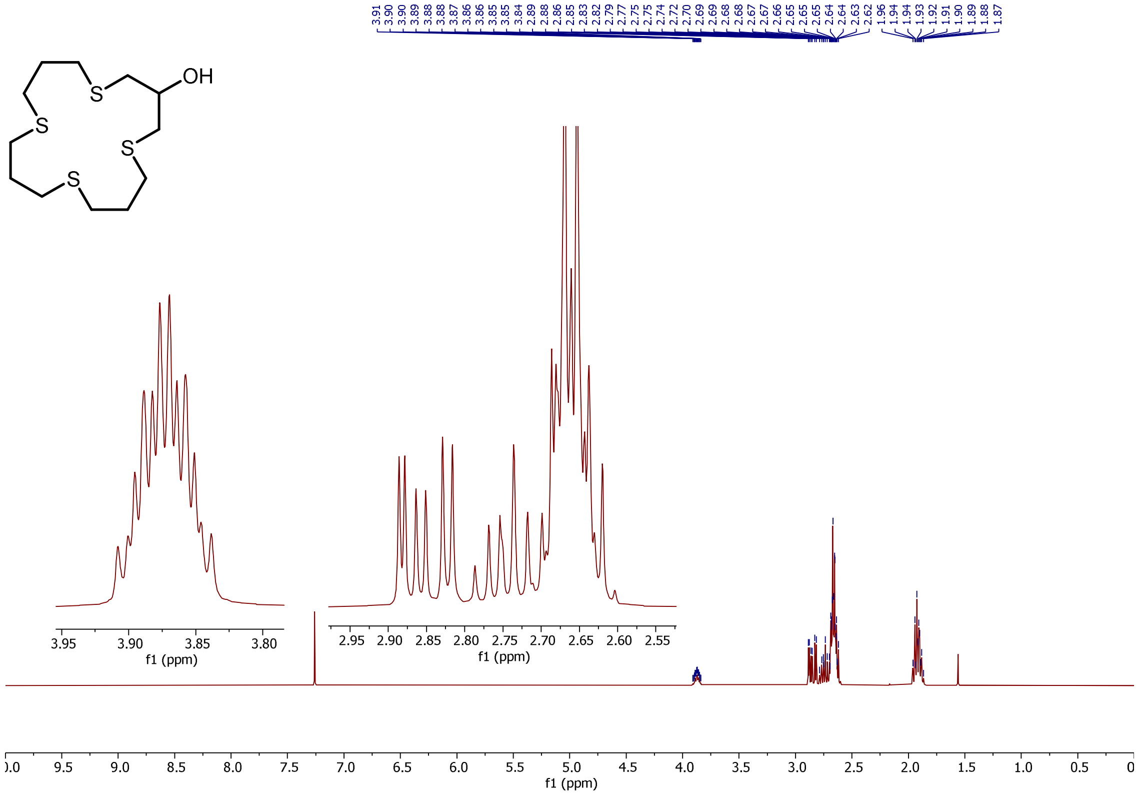
OH
S
S
S
S
3.91 3.90 3.90 3.89 3.88 3.88 3.87 3.86 3.86 3.85 3.85 3.84 2.89 2.88 2.86 2.85 2.83 2.82 2.79 2.77 2.75 2.75 2.74 2.72 2.70 2.69 2.69 2.68 2.68 2.67 2.67 2.66 2.65 2.65 2.65 2.64 2.64 2.63 2.62 1.96 1.94 1.94 1.93 1.92 1.91 1.90 1.89 1.88 1.87
f1 (ppm)
f1 (ppm)
f1 (ppm)

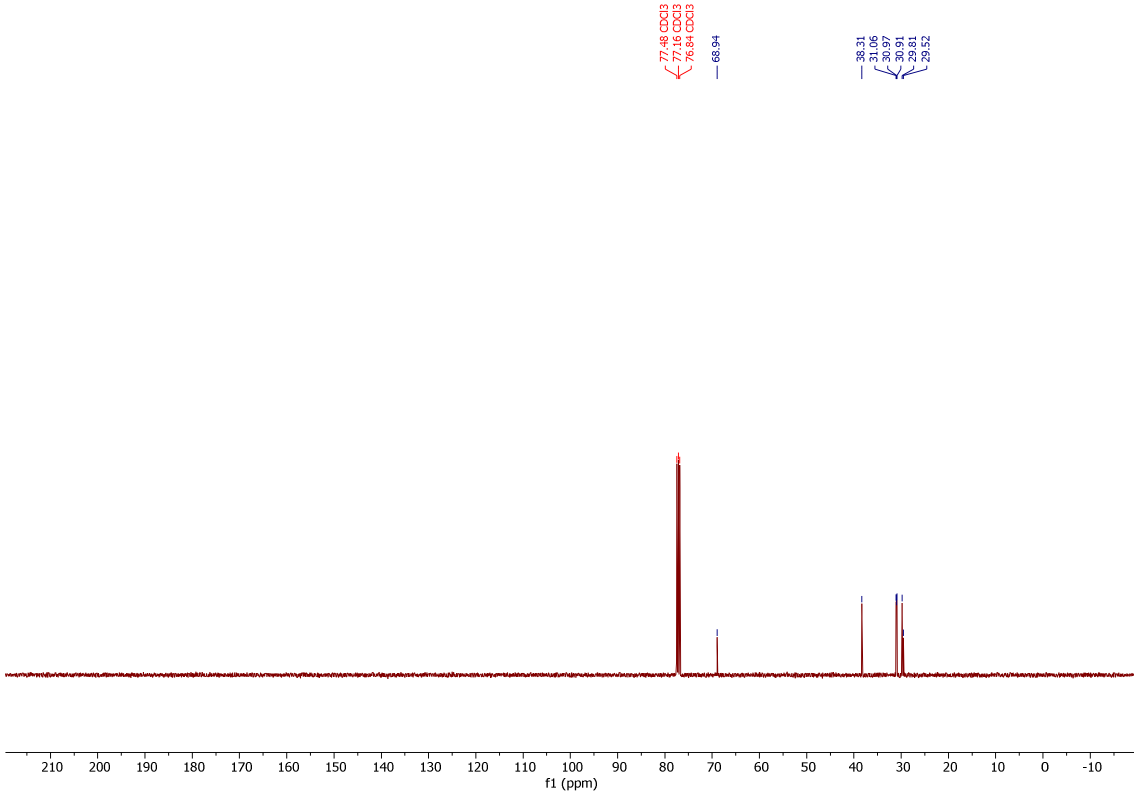
77.48 CDCl3
77.16 CDCl3
76.84 CDCl3
68.94
38.31
31.06
30.97
30.91
29.81
29.52
f1 (ppm)

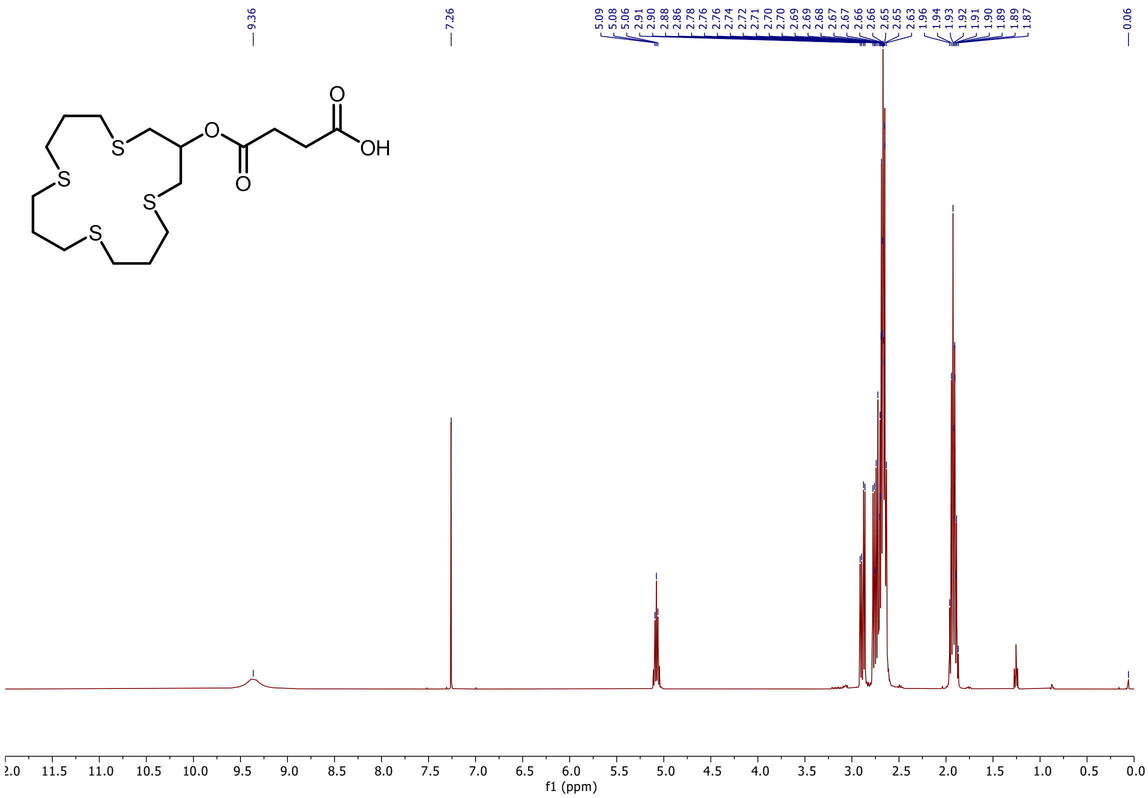
9.36
7.26
5.09 5.08 5.06 2.91 2.90 2.88 2.86 2.78 2.76 2.76 2.74 2.72 2.71 2.70 2.69 2.69 2.68 2.67 2.67 2.66 2.66 2.65 2.65 2.63 1.96 1.94 1.93 1.92 1.91 1.90 1.89 1.87
0.06
S
S
S
S
O
O
O
OH
[illegible].0 11.5 11.0 10.5 10.0 9.5 9.0 8.5 8.0 7.5 7.0 6.5 6.0 5.5 5.0 4.5 4.0 3.5 3.0 2.5 2.0 1.5 1.0 0.5 0.0
f1 (ppm)

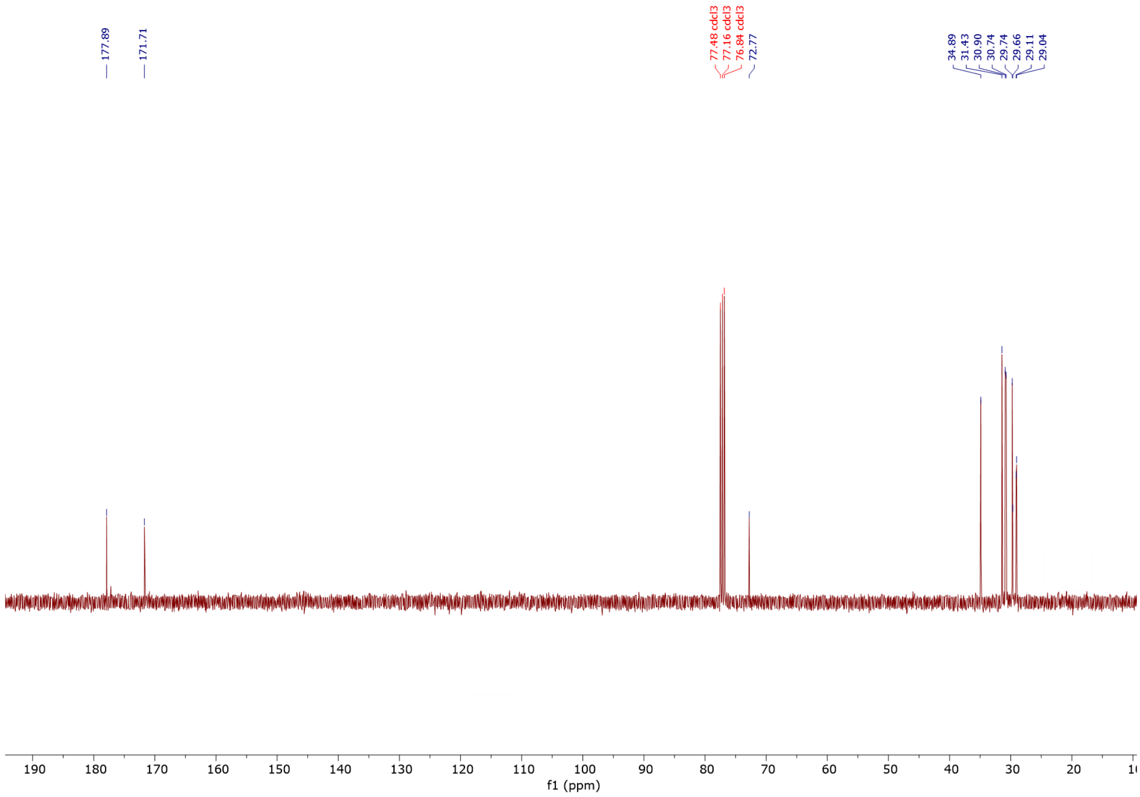
177.89
171.71
77.48 cdcl3
77.16 cdcl3
76.84 cdcl3
72.77
34.89 31.43 30.90 30.74 29.74 29.66 29.11 29.04
190 180 170 160 150 140 130 120 110 100 90 80 70 60 50 40 30 20
f1 (ppm)

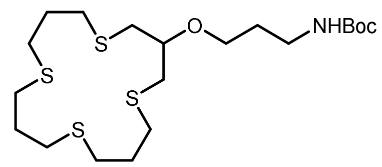
S
S
S
S
O
NHBoc

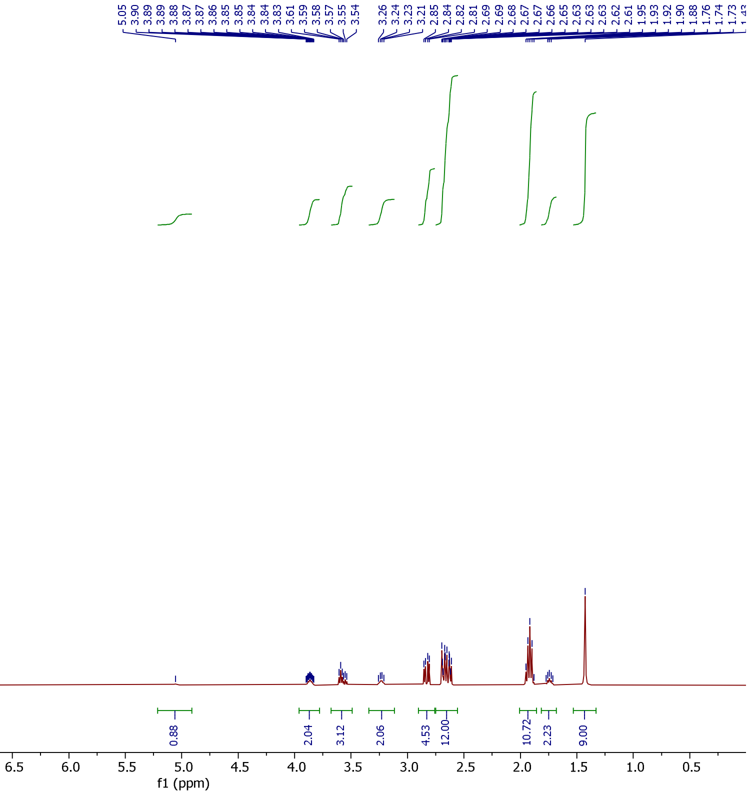
7.26
5.05 3.90 3.89 3.89 3.88 3.87 3.87 3.86 3.85 3.85 3.84 3.84 3.83 3.61 3.59 3.58 3.57 3.55 3.54
3.26 3.24 3.23 3.21 2.85 2.84 2.82 2.81 2.69 2.69 2.68 2.67 2.67 2.66 2.65 2.63 2.63 2.63 2.62 2.61 1.95 1.93 1.92 1.90 1.88 1.76 1.74 1.73
0.88
2.04
3.12
2.06
4.53
12.00
10.72
2.23
9.00
9.5 9.0 8.5 8.0 7.5 7.0 6.5 6.0 5.5 5.0 4.5 4.0 3.5 3.0 2.5 2.0 1.5 1.0 0.5
f1 (ppm)

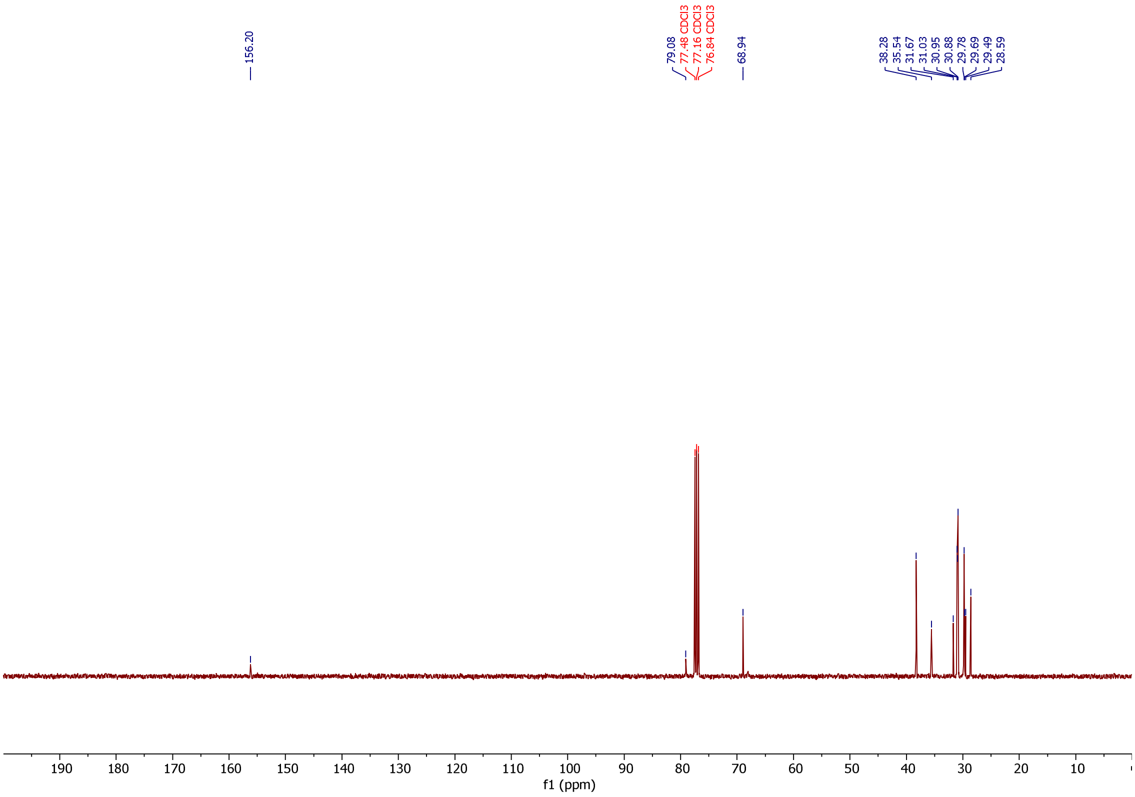
156.20
79.08
77.48 CDCl3
77.16 CDCl3
76.84 CDCl3
68.94
38.28 35.54 31.67 31.03 30.95 30.88 29.78 29.69 29.49 28.59
190 180 170 160 150 140 130 120 110 100 90 80 70 60 50 40 30 20 10
f1 (ppm)

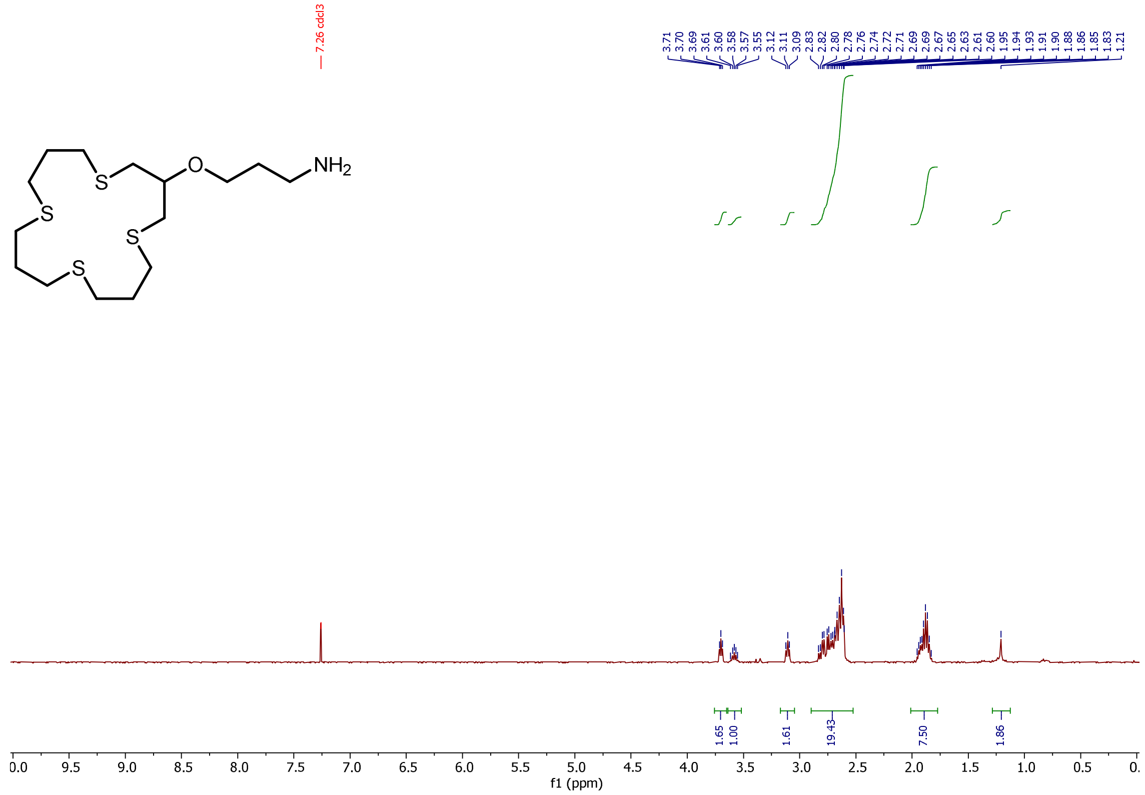
S
S
S
S
O
NH2
7.26 cdcl3
3.71
3.70
3.69
3.61
3.60
3.58
3.57
3.55
3.12
3.11
3.09
2.83
2.82
2.80
2.78
2.76
2.74
2.72
2.71
2.69
2.69
2.67
2.65
2.63
2.61
2.60
1.95
1.94
1.93
1.91
1.90
1.88
1.86
1.85
1.83
1.21
1.65
1.00
1.61
19.43
7.50
1.86
f1 (ppm)

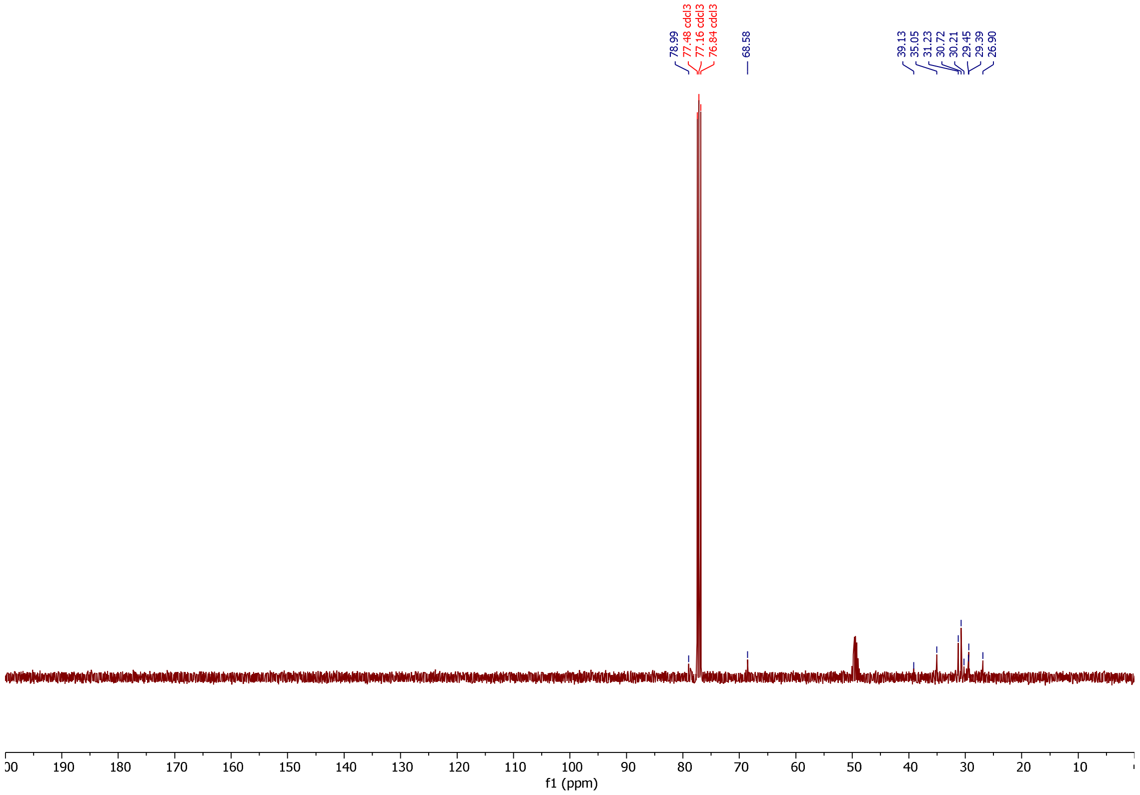
78.99
77.48 cdcl3
77.16 cdcl3
76.84 cdcl3
68.58
39.13
35.05
31.23
30.72
30.21
29.45
29.39
26.90
f1 (ppm)

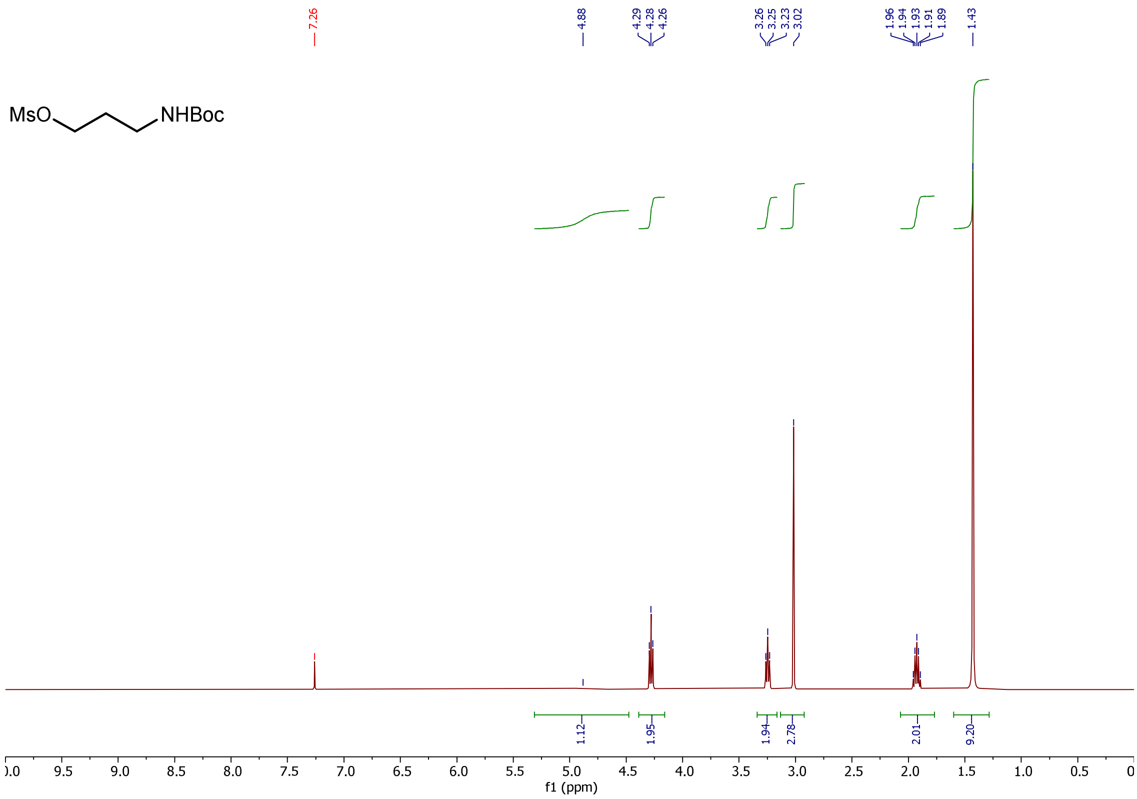
MsO
NHBoc
7.26
4.88
4.29
4.28
4.26
3.26
3.25
3.23
3.02
1.96
1.94
1.93
1.91
1.89
1.43
1.12
1.95
1.94
2.78
2.01
9.20
f1 (ppm)

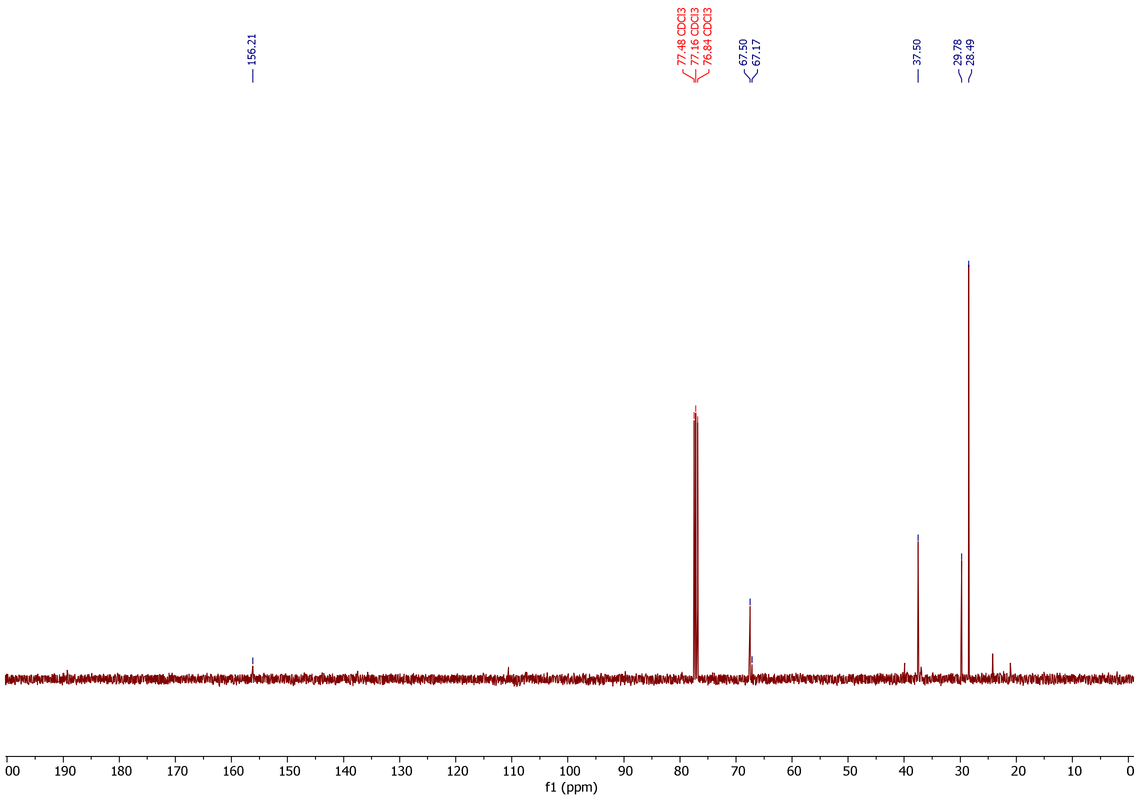
156.21
77.48 CDCl3
77.16 CDCl3
76.84 CDCl3
67.50
67.17
37.50
29.78
28.49
f1 (ppm)

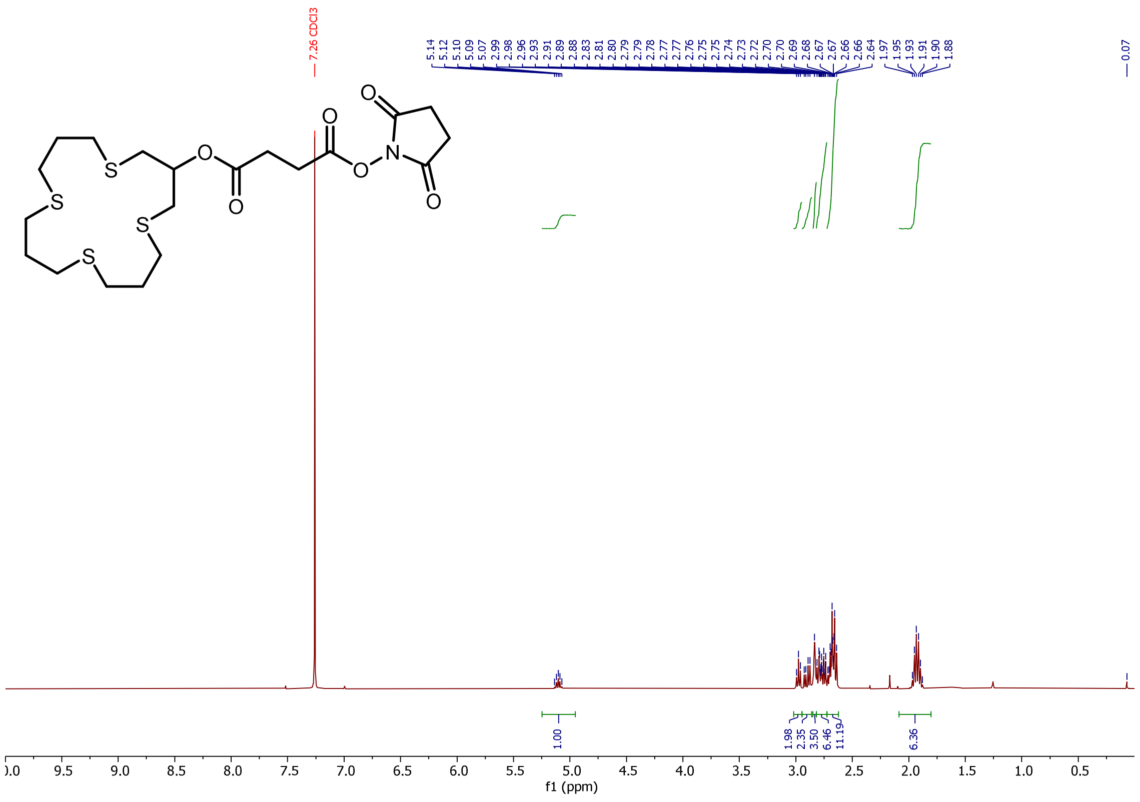

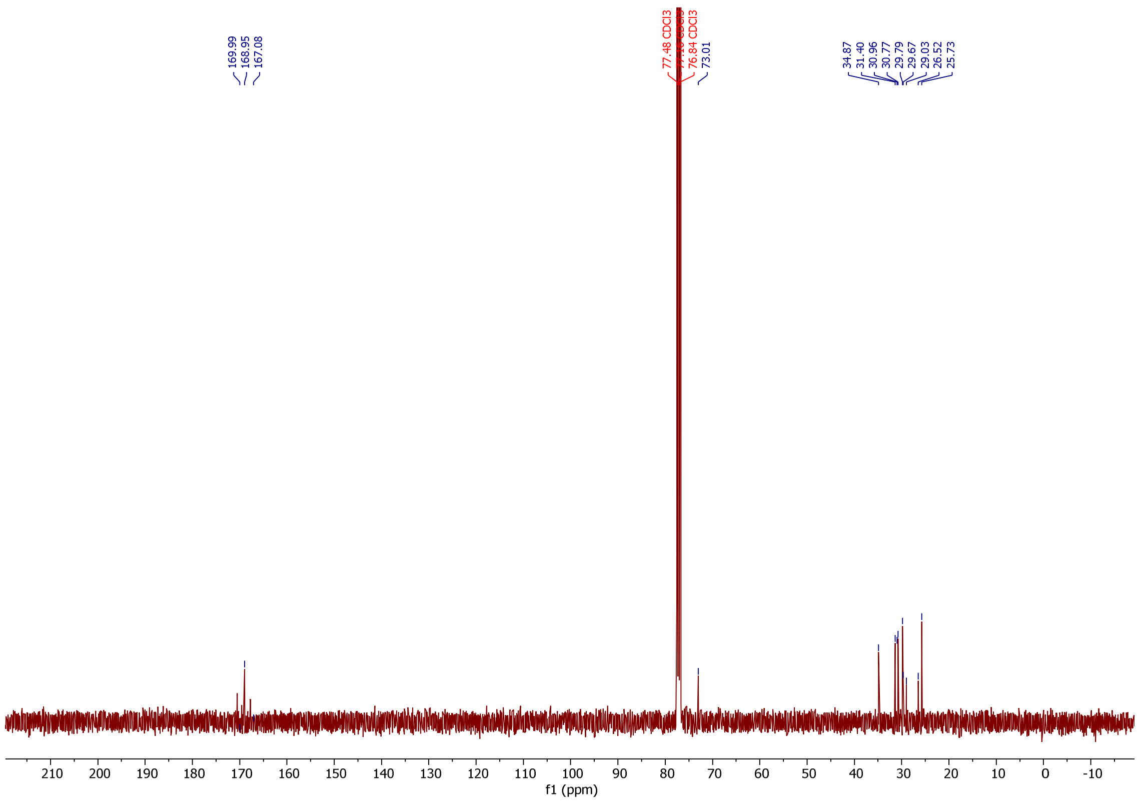

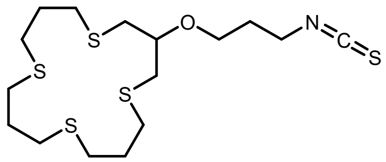
S
O
N
C
S
S
S
S

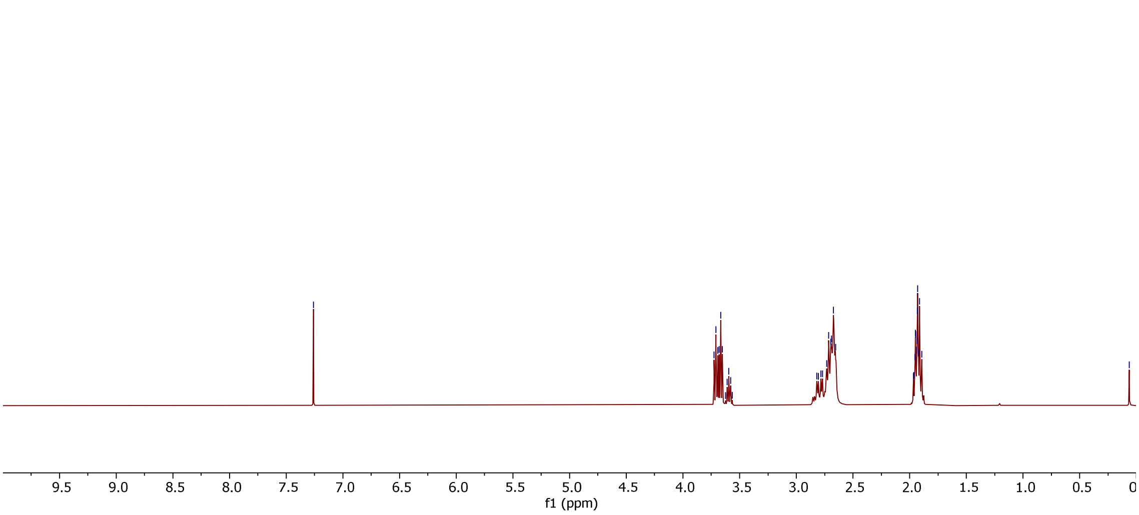
7.26
3.72
3.71
3.69
3.68
3.67
3.65
3.62
3.61
3.59
3.58
3.57
2.82
2.80
2.78
2.77
2.73
2.71
2.70
2.69
2.67
2.65
1.97
1.96
1.95
1.95
1.95
1.94
1.94
1.93
1.93
1.92
1.91
1.89
0.06
9.5 9.0 8.5 8.0 7.5 7.0 6.5 6.0 5.5 5.0 4.5 4.0 3.5 3.0 2.5 2.0 1.5 1.0 0.5 0
f1 (ppm)

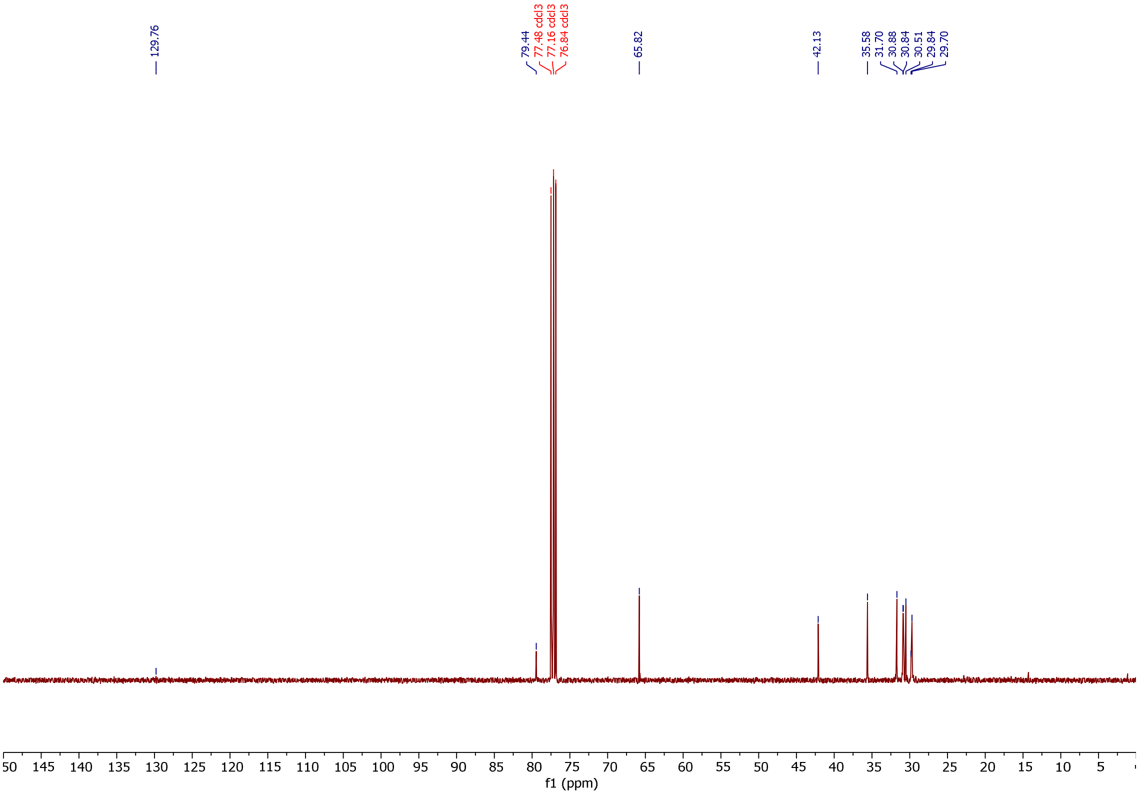
129.76
79.44
77.48 cdcl3
77.16 cdcl3
76.84 cdcl3
65.82
42.13
35.58
31.70
30.88
30.84
30.51
29.84
29.70
150 145 140 135 130 125 120 115 110 105 100 95 90 85 80 75 70 65 60 55 50 45 40 35 30 25 20 15 10 5
f1 (ppm)

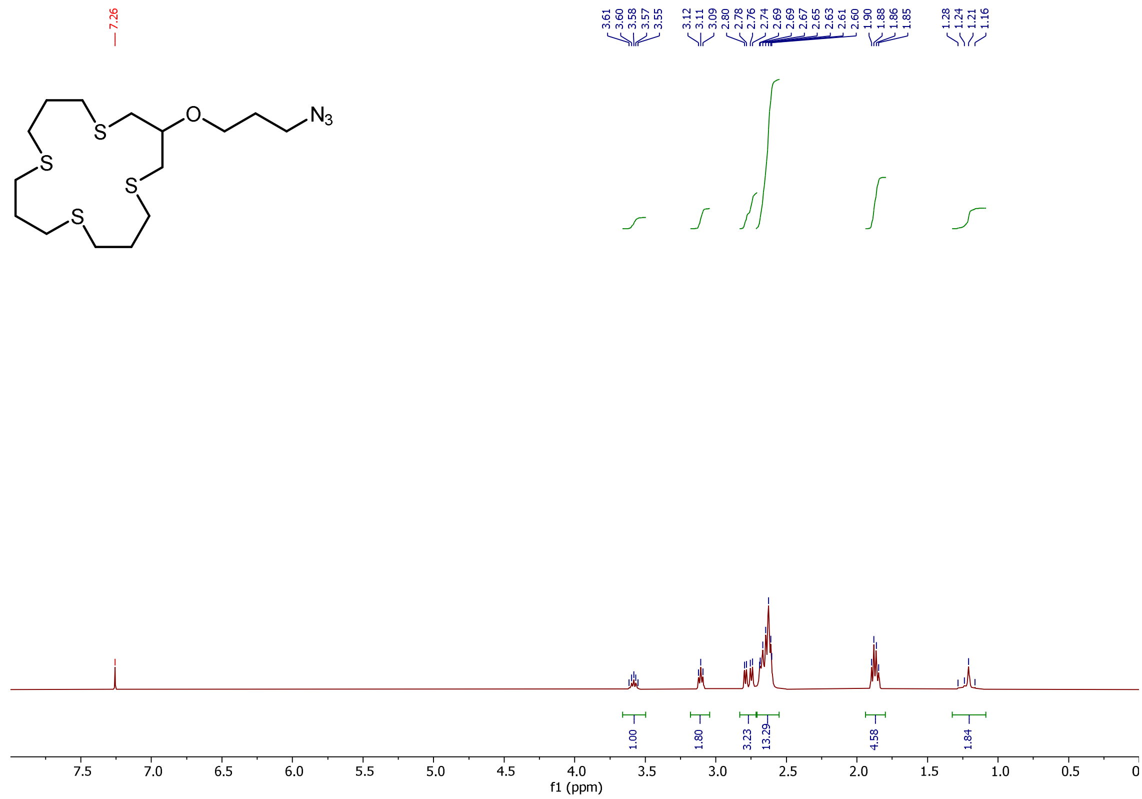
7.26
3.61 3.60 3.58 3.57 3.55 3.12 3.11 3.09 2.80 2.78 2.76 2.74 2.69 2.69 2.67 2.65 2.63 2.61 2.60 1.90 1.88 1.86 1.85 1.28 1.24 1.21 1.16
O
S
S
S
S
N_3
1.00 1.80 3.23 13.29 4.58 1.84
f1 (ppm)

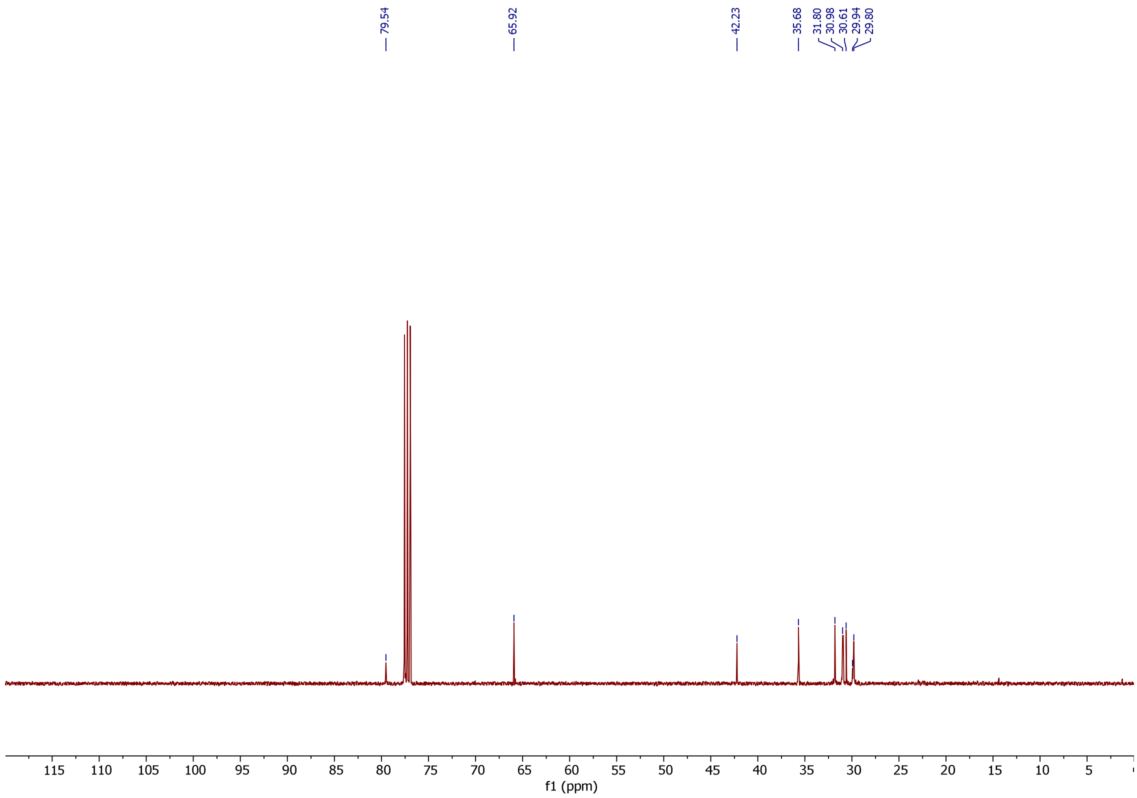
79.54 65.92 42.23 35.68 31.80 30.98 30.61 29.94 29.80
f1 (ppm)

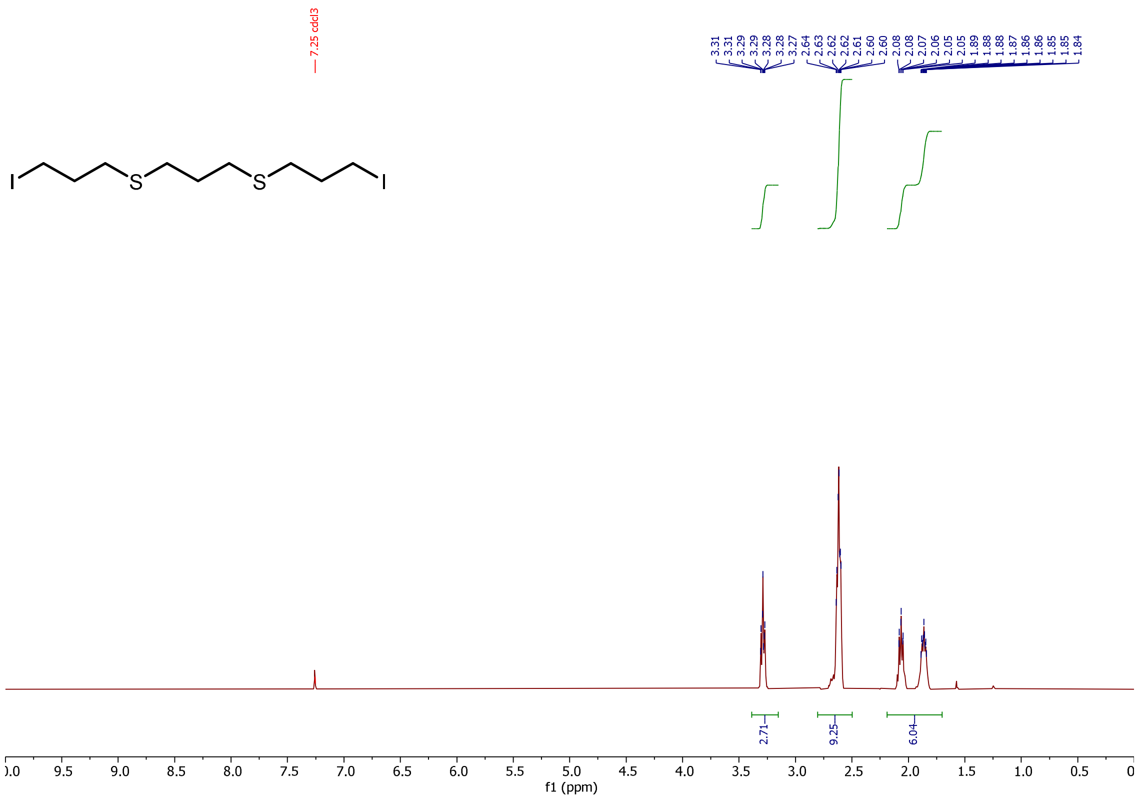
7.25 cdcl3
3.31
3.31
3.29
3.28
3.28
3.27
2.64
2.63
2.62
2.62
2.61
2.60
2.60
2.08
2.08
2.07
2.06
2.05
2.05
1.89
1.88
1.88
1.87
1.86
1.86
1.85
1.85
1.84
2.71
9.25
6.04
f1 (ppm)

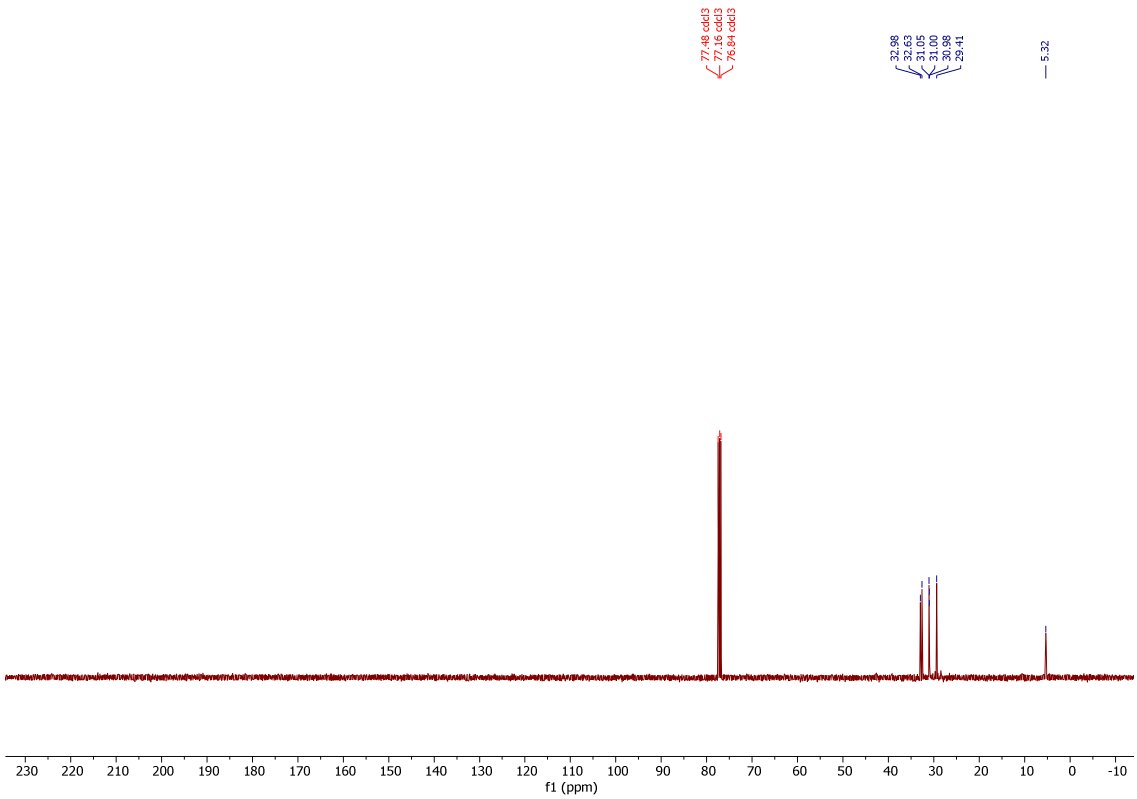
77.48 cdcl3
77.16 cdcl3
76.84 cdcl3
32.98
32.63
31.05
31.00
30.98
29.41
5.32
f1 (ppm)

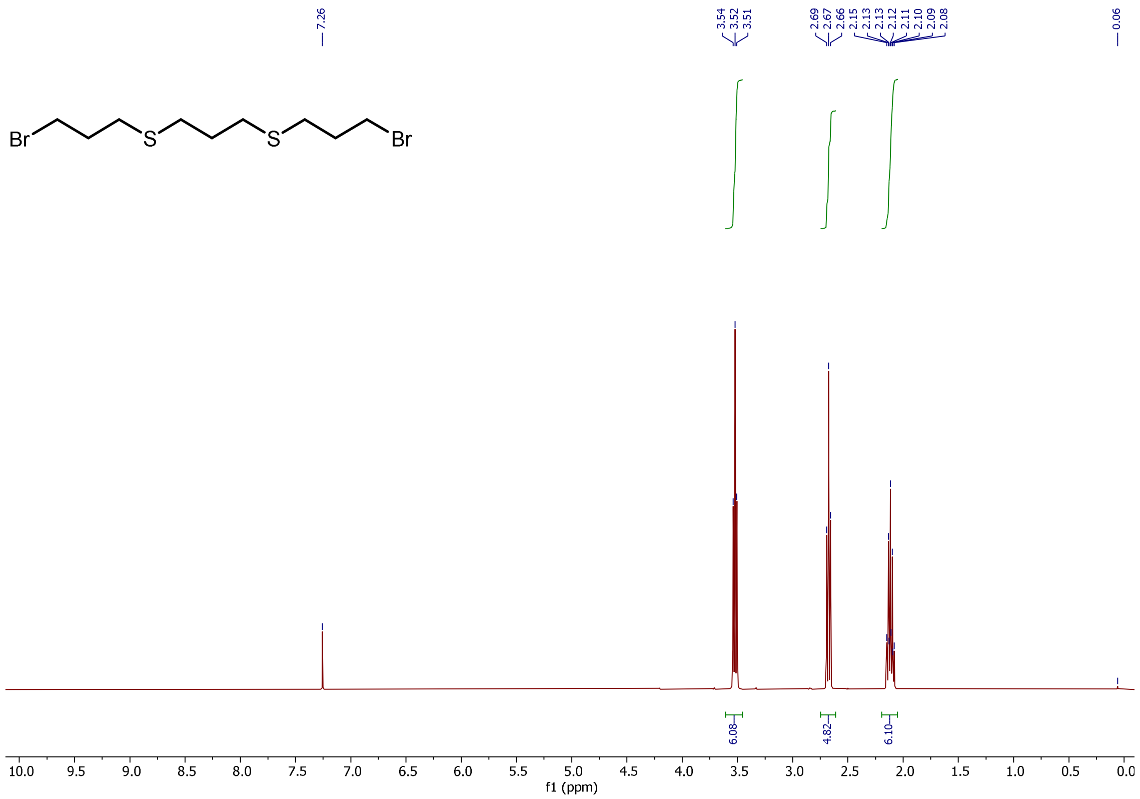
Br
S
S
Br
7.26
3.54
3.52
3.51
2.69
2.67
2.66
2.15
2.13
2.13
2.12
2.11
2.10
2.09
2.08
0.06
6.08
4.82
6.10
f1 (ppm)

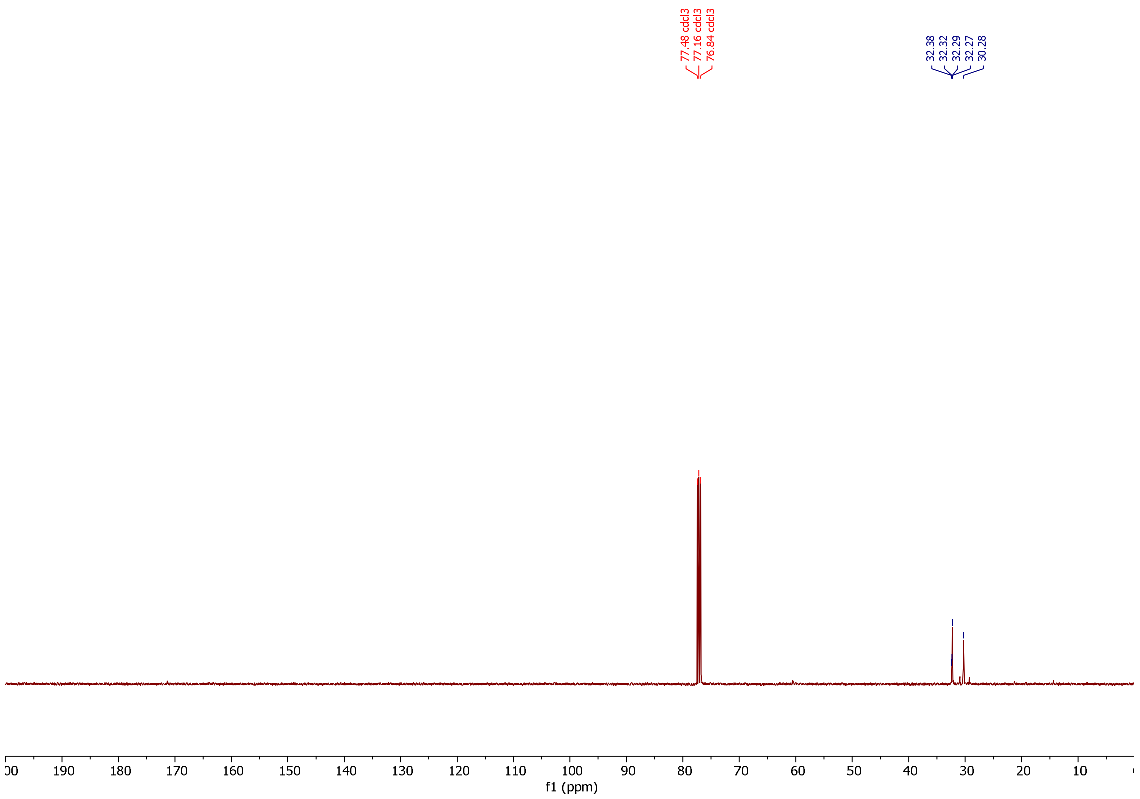
77.48 cdcl3
77.16 cdcl3
76.84 cdcl3
32.38
32.32
32.29
32.27
30.28
f1 (ppm)

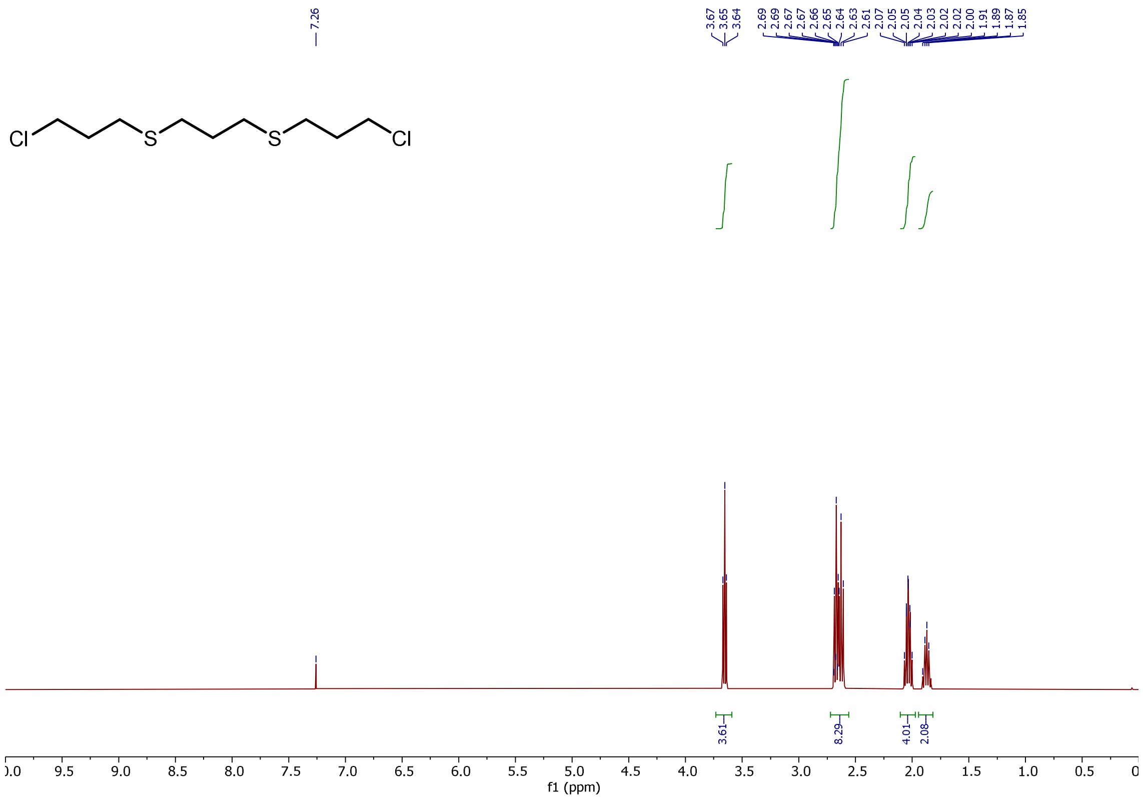
7.26
3.67
3.65
3.64
2.69
2.69
2.67
2.67
2.66
2.65
2.64
2.63
2.61
2.07
2.05
2.05
2.04
2.03
2.02
2.02
2.00
1.91
1.89
1.87
1.85
Cl
S
S
Cl
3.61
8.29
4.01
2.08
f1 (ppm)

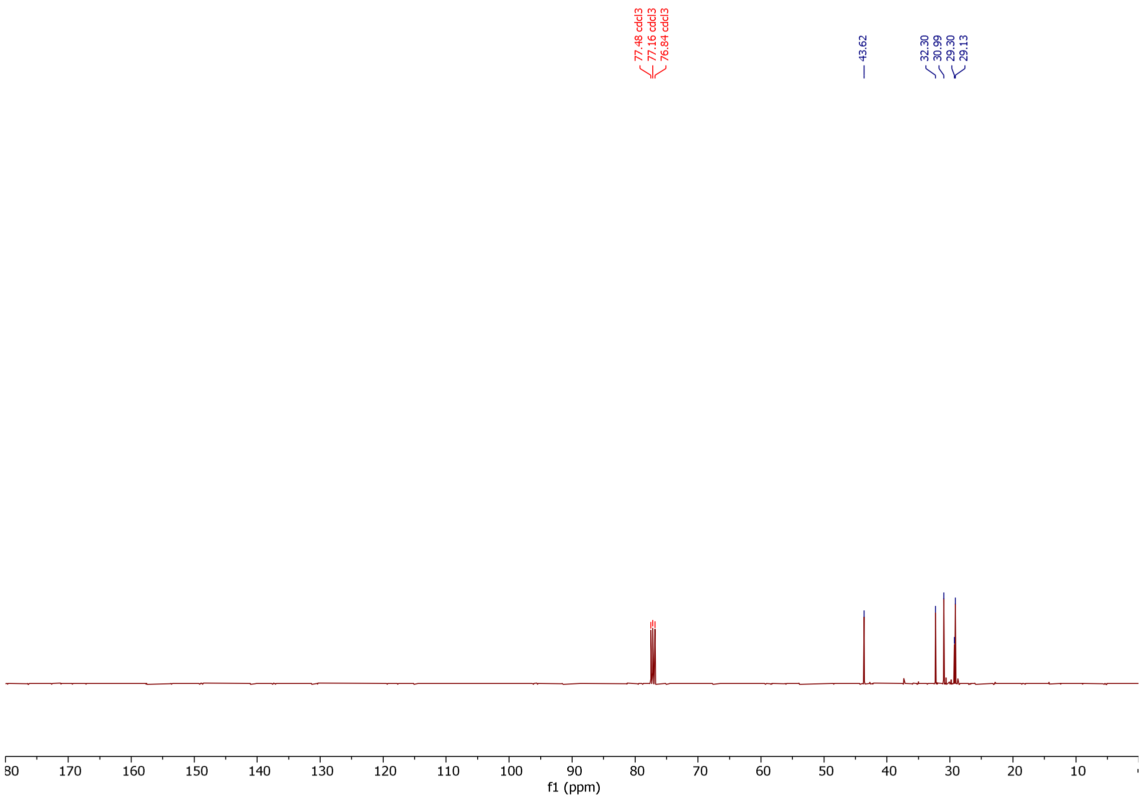
77.48 cdcl3
77.16 cdcl3
76.84 cdcl3
43.62
32.30
30.99
29.30
29.13
f1 (ppm)

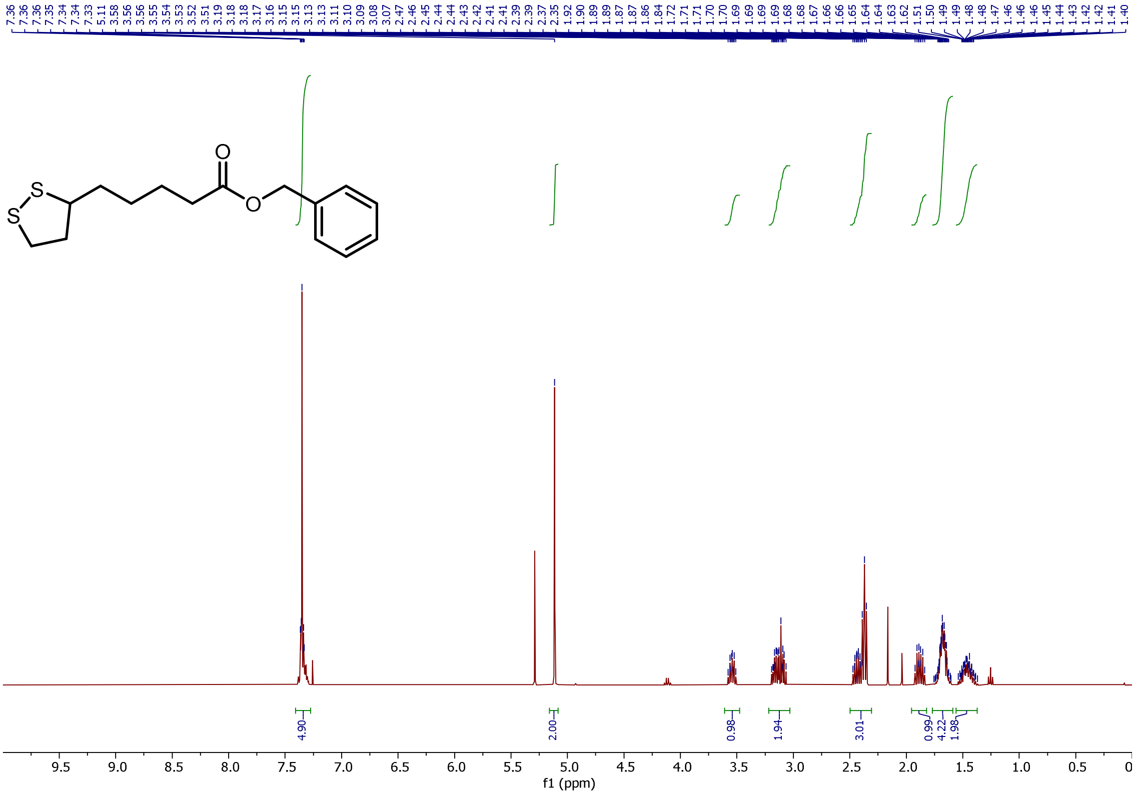
7.36 7.36 7.36 7.35 7.34 7.34 7.33 5.11 3.58 3.56 3.56 3.55 3.54 3.53 3.52 3.51 3.19 3.18 3.18 3.17 3.16 3.15 3.15 3.13 3.13 3.11 3.10 3.09 3.08 3.07 2.47 2.46 2.45 2.44 2.44 2.43 2.42 2.41 2.41 2.39 2.39 2.37 2.35 1.92 1.90 1.89 1.89 1.87 1.87 1.86 1.84 1.72 1.71 1.71 1.70 1.70 1.69 1.69 1.69 1.69 1.68 1.68 1.67 1.66 1.66 1.65 1.64 1.64 1.63 1.62 1.51 1.50 1.49 1.49 1.48 1.47 1.46 1.46 1.45 1.44 1.43 1.42 1.42 1.41 1.40
S
S
O
O
4.90
2.00
0.98
1.94
3.01
0.99
4.22
1.98
f1 (ppm)

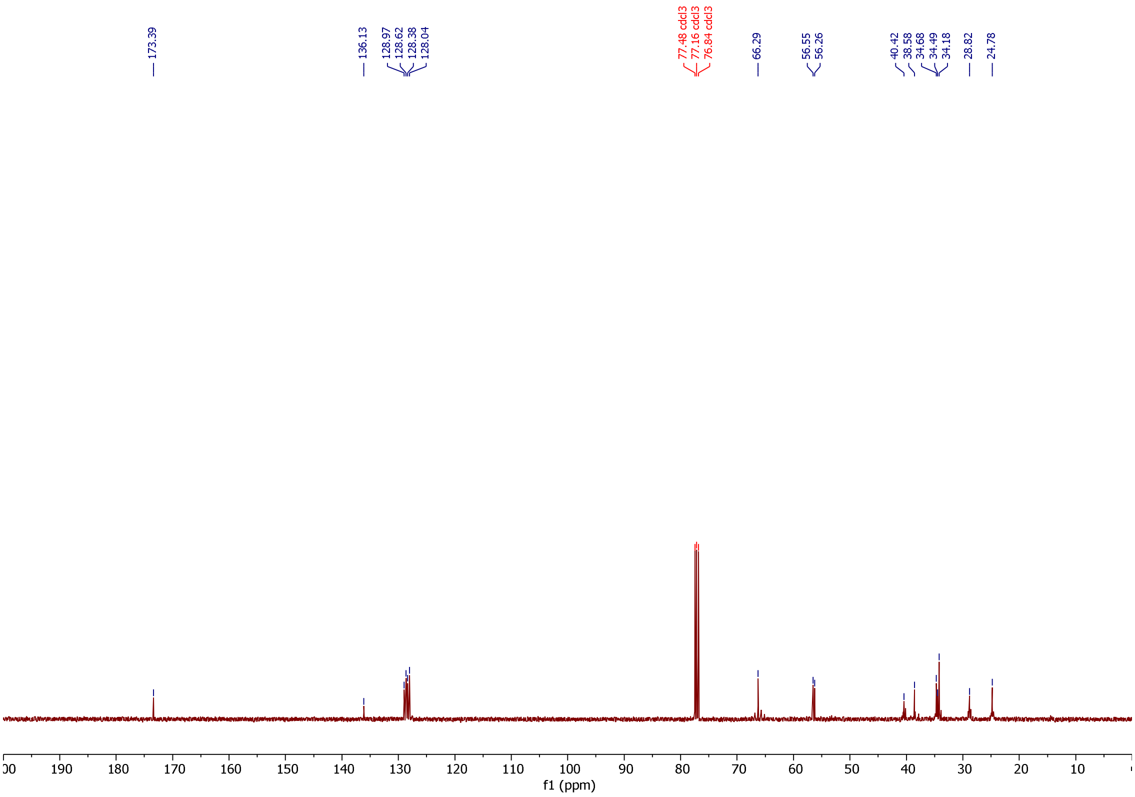
173.39
136.13 128.97 128.62 128.38 128.04
77.48 cdcl3
77.16 cdcl3
76.84 cdcl3
66.29
56.55 56.26
40.42 38.58 34.68 34.49 34.18
28.82
24.78
f1 (ppm)

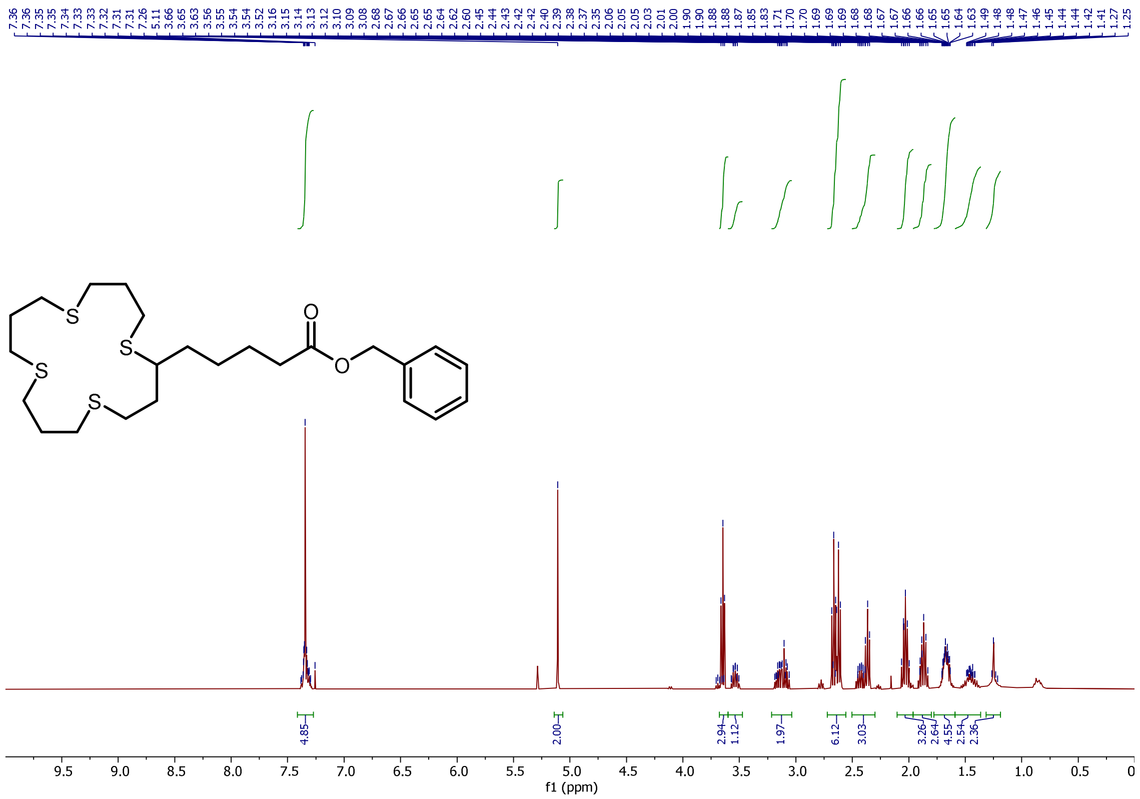

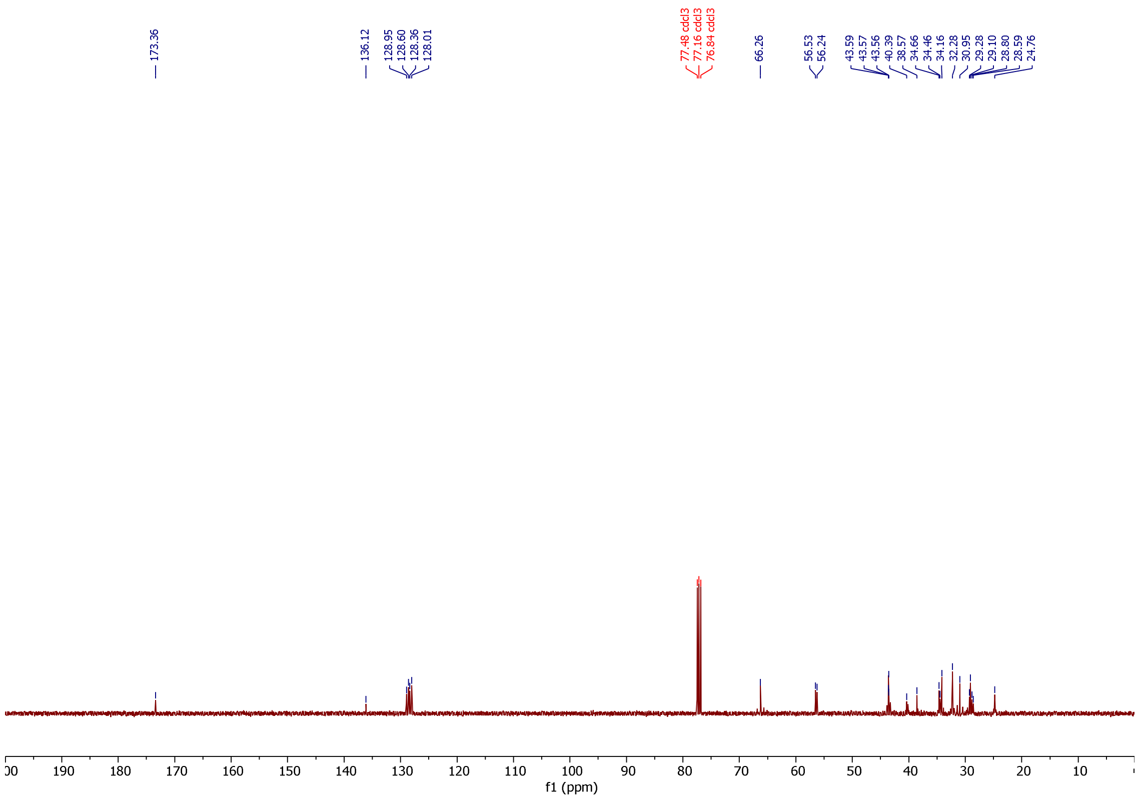

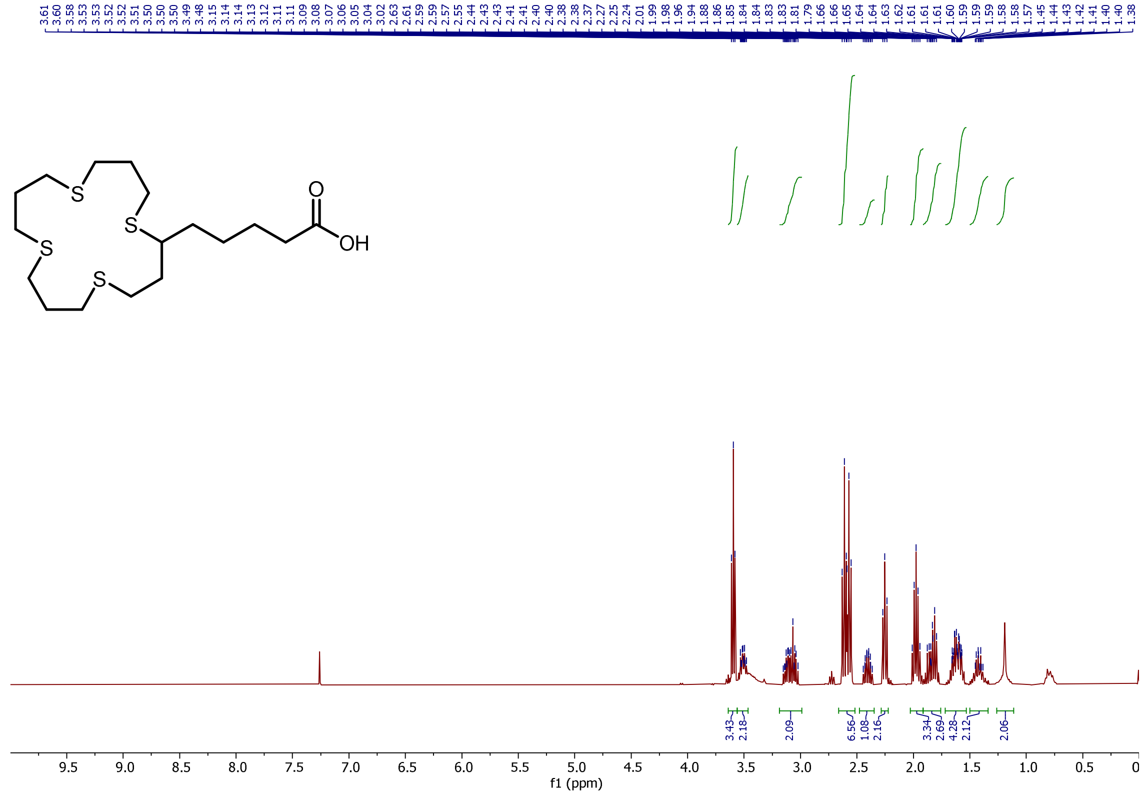
S
S
S
S
O
OH
3.61
3.60
3.58
3.53
3.53
3.52
3.51
3.50
3.50
3.49
3.48
3.15
3.14
3.13
3.12
3.11
3.09
3.08
3.07
3.06
3.05
3.04
3.02
2.63
2.61
2.59
2.59
2.57
2.55
2.44
2.43
2.43
2.41
2.40
2.38
2.38
2.37
2.27
2.25
2.24
2.01
1.99
1.98
1.96
1.94
1.88
1.86
1.85
1.84
1.84
1.83
1.83
1.81
1.79
1.66
1.65
1.64
1.64
1.63
1.62
1.61
1.61
1.61
1.60
1.59
1.59
1.58
1.58
1.57
1.45
1.44
1.43
1.42
1.41
1.40
1.40
1.38
3.43
2.18
2.09
6.56
1.08
2.16
3.34
2.69
4.28
2.12
2.06
9.5 9.0 8.5 8.0 7.5 7.0 6.5 6.0 5.5 5.0 4.5 4.0 3.5 3.0 2.5 2.0 1.5 1.0 0.5 0
f1 (ppm)

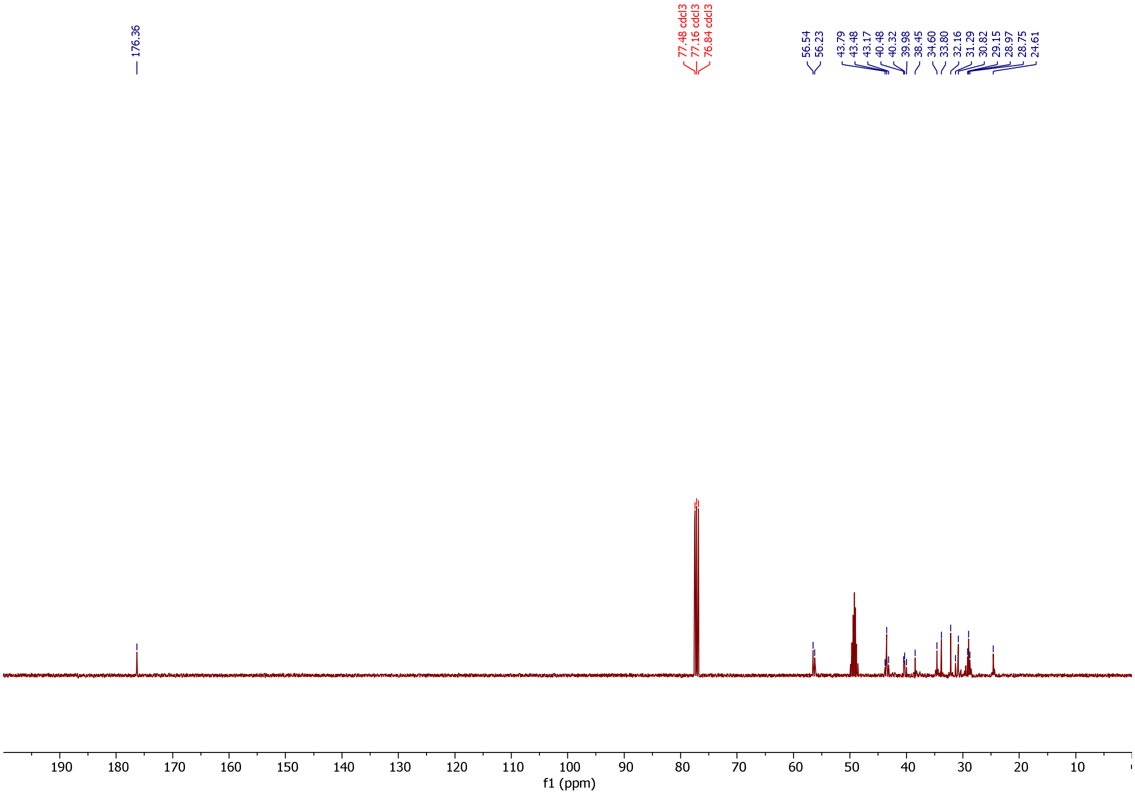
176.36
77.48 cdcl3
77.16 cdcl3
76.84 cdcl3
56.54
56.23
43.79
43.48
43.17
40.48
40.32
39.98
38.45
34.60
33.80
32.16
31.29
30.82
29.15
28.97
28.75
24.61
190 180 170 160 150 140 130 120 110 100 90 80 70 60 50 40 30 20 10
f1 (ppm)
